# The Novel Chiral 2(5*H*)-Furanone Sulfones Possessing Terpene Moiety: Synthesis and Biological Activity

**DOI:** 10.3390/molecules28062543

**Published:** 2023-03-10

**Authors:** Alsu M. Khabibrakhmanova, Roza G. Faizova, Olga A. Lodochnikova, Regina R. Zamalieva, Liliya Z. Latypova, Elena Y. Trizna, Andrey G. Porfiryev, Katsunori Tanaka, Oskar A. Sachenkov, Airat R. Kayumov, Almira R. Kurbangalieva

**Affiliations:** 1 Biofunctional Chemistry Laboratory, A. Butlerov Institute of Chemistry, Kazan Federal University, 18 Kremlyovskaya Street, 420008 Kazan, Russia; 2 Arbuzov Institute of Organic and Physical Chemistry, FRC Kazan Scientific Center of RAS, 8 Arbuzov Street, 420088 Kazan, Russia; 3 Institute of Fundamental Medicine and Biology, Kazan Federal University, 18 Kremlyovskaya Street, 420008 Kazan, Russia; 4 Biofunctional Synthetic Chemistry Laboratory, RIKEN Cluster for Pioneering Research, 2-1 Hirosawa, Wako-shi 351-0198, Japan; 5 Department of Chemical Science and Engineering, School of Materials and Chemical Technology, Tokyo Institute of Technology, 2-12-1 O-okayama, Meguro-ku, Tokyo 152-8552, Japan; 6 N.I. Lobachevsky Institute of Mathematics and Mechanics, Kazan Federal University, 18 Kremlyovskaya Street, 420008 Kazan, Russia

**Keywords:** 2(5*H*)-furanone, lactone, sulfone, stereoisomer, antimicrobial activity, synergism with antimicrobials, wound healing, X-ray diffraction analysis

## Abstract

Over the past decades, 2(5*H*)-furanone derivatives have been extensively studied because of their promising ability to prevent the biofilm formation by various pathogenic bacteria. Here, we report the synthesis of a series of optically active sulfur-containing 2(5*H*)-furanone derivatives and characterize their biological activity. Novel thioethers were obtained by an interaction of stereochemically pure 5-(*l*)-menthyloxy- or 5-(*l*)-bornyloxy-2(5*H*)-furanones with aromatic thiols under basic conditions. Subsequent thioethers oxidation by an excess of hydrogen peroxide in acetic acid resulted in the formation of the corresponding chiral 2(5*H*)-furanone sulfones. The structure of synthesized compounds was confirmed by IR and NMR spectroscopy, HRMS, and single crystal X-ray diffraction. The leading compound, **26**, possessing the sulfonyl group and *l*-borneol moiety, exhibited the prominent activity against *Staphylococcus aureus* and *Bacillus subtilis* with MICs of 8 μg/mL. Furthermore, at concentrations of 0.4–0.5 μg/mL, the sulfone **26** increased two-fold the efficacy of aminoglycosides gentamicin and amikacin against *S. aureus*. The treatment of the model-infected skin wound in the rat with a combination of gentamicin and sulfone **26** speeded up the bacterial decontamination and improved the healing of the wound. The presented results provide valuable new insights into the chemistry of 2(5*H*)-furanone derivatives and associated biological activities.

## 1. Introduction

Sulfur-containing compounds are very diverse and widespread in the environment: they are naturally produced by both plants and animals, as well as by microorganisms. These compounds are becoming increasingly important as the significant role of sulfur is shown for various biological processes, chemical synthesis, and new materials. Natural and synthetic organosulfur substances are widely used in organic synthesis, medicinal chemistry, pharmacology, materials science, agriculture, technology, etc. [1,2,3,4,5,6,7,8,9,10,11]. Thus, the protective effects of *Alliums* are largely associated with the presence of such organosulfur compounds as diallyl sulfide and diallyl disulfide [12,13,14,15], that in turn leads to reduced susceptibility to cancer of people who consume large amounts of *Allium* vegetables (onions, garlic, etc.). The C–S bond is also present in the molecules of several drugs that are used to treat Alzheimer’s, Parkinson’s diseases, and HIV [3,4]. Moreover, organosulfur compounds are usually used in herbal medicine, as flavoring agents and preservatives in the food industry, and also as ligands and spacers in the preparation of metal coordination complexes and organometallic framework structures [15,16,17,18,19].

**Figure 1:**
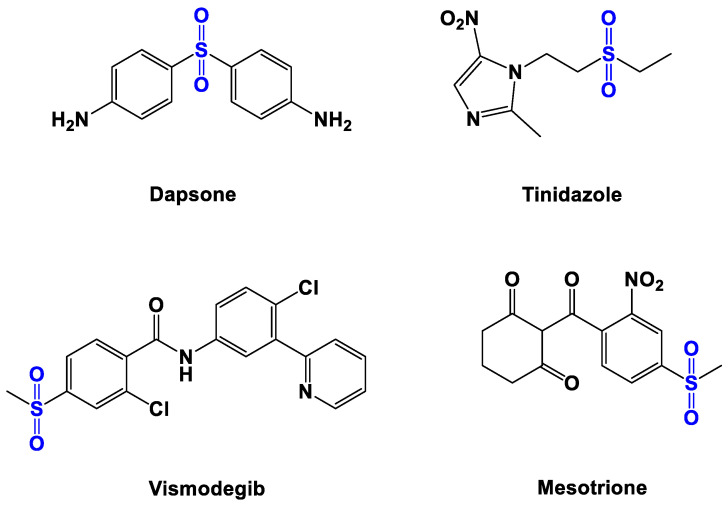
Sulfone containing scaffolds in pharmaceutical molecules.

The oxidation products of organic sulfides (thioethers)–sulfones are particularly attractive. Sulfonyl-containing intermediates are widely used in the synthesis of natural and other biologically active substances (drugs, agrochemicals) because of their availability, versatility, easy chemical modification, high reactivity, and the possibility of an easy removal at a desired stage [20,21,22,23]. The sulfonyl group does not have its own asymmetry, but due to its unique bulk and electronic properties, it is able to exercise regio- and stereocontrol in reactions. Among sulfones, highly effective insect repellents, herbicides for a number of crops, solvents, polymers, and pharmaceuticals were found. Sulfonyl-containing compounds also exhibit significant antimicrobial, antifungal, antimalarial, anti-inflammatory, and anti-cancer activity, etc. [1,2,3,4,5,22,24,25,26,27]. The several drugs possessing the SO_2_ group are used to treat leprosy, dermatitis herpetiformis, and tuberculosis. For example, a diarylsulfone derivative known as Dapsone is used in the treatment of infectious diseases (leprosy and malaria) and skin and non-communicable inflammatory diseases, and it is included in the WHO Model Lists of Essential Medicines [4,28,29] (Figure 1). Another bioactive sulfonyl-containing compound, Tinidazole, is an antimicrobial and antiprotozoal agent. This antibiotic is a prodrug that was approved by the FDA in 2004 [1,30]. Vismodegib (Erivedge^®^) is an effective and generally well-tolerated medication for patients with basal cell carcinoma (BCC) [1,31], while mesotrione is a selective herbicide for the control of certain weeds [2].

The combination of a sulfonyl group and an unsaturated γ-lactone moiety is an attractive approach to increase or diversify the biological activity exhibited. Among 2(5*H*)-furanone derivatives, substances with a wide spectrum of biological activity have been identified, including anti-inflammatory, antitumor, antimicrobial, antifungal, antioxidant, anticonvulsant, analgesic, antituberculosis, antiulcer, anti-HIV activity, etc. [32,33,34,35,36]. For example, several heterocycles possessing an unsaturated lactone ring and a sulfur-containing fragment, including those with chiral monoterpene alcohol residue, are described in the literature as compounds exhibiting a pronounced inhibitory effect on breast cancer cell lines MCF-7 [37] and cervical cancer cell lines HeLa [38,39], as well as human hepatocellular carcinoma SMMC-7721 [39,40].

The rapid development and spread of antibiotic resistance require the development of new antimicrobial drugs and therapeutic approaches [41,42]. The 2(5*H*)-furanone derivatives have been actively studied in the last two decades as potential antimicrobial- and biofilm-preventing agents [43,44]. Thus, many natural (isolated from the red algae *Delisea pulchra*) and synthetic 2(5*H*)-furanone derivatives have been reported to repress the formation of biofilms by various Gram-negative and Gram-positive bacteria, such as *Staphylococcus aureus*, *Staphylococcus epidermidis*, *Bacillus subtilis*, *Bacillus cereus*, *Escherichia coli*, and *Pseudomonas aeruginosa*, etc. [45,46,47,48,49,50,51,52,53,54,55,56,57,58,59]. However, the most important factor limiting the clinical use of halogenated furanones as antimicrobials is their toxicity to eukaryotic cells and low stability in aqueous solutions [36,47,50]. Therefore, the search and study of derivatives with high activity and low toxicity is still challenging.

We have recently shown that chiral sulfone based on 2(5*H*)-furanone and *l*-menthol (3-chloro-5(*S*)*-*[(1*R,*2*S,*5*R*)*-*2-isopropyl-5-methylcyclohexyloxy]*-*4-[4-methylphenylsulfo-nyl]-2(5*H*)*-*furanone, denoted as **F105**) has antimicrobial activity against planktonic and biofilm-embedded methicillin-resistant and susceptible *S. aureus* [60,61]. Minimal inhibitory and bactericidal concentration values (MIC and MBC) of furanone **F105** were 10 and 40 μg/mL, respectively, suggesting that **F105** has biocidal properties. In addition, we found a synergy of compound **F105** with aminoglycosides (amikacin, gentamicin, and kanamycin) and benzalkonium chloride against planktonic *S. aureus* and demonstrated their attractive activity toward the biofilm-embedded bacteria. This makes it possible to use the furanone **F105** for the development of complex topical agents for combined anti-staphylococcal biofilm-therapies [60]. The 2(5*H*)-furanone derivative **F105** also showed moderate antifungal activity against some strains of *Candida albicans*, as well as synergism with typical antifungal agents such as fluconazole and terbinafine reducing the MIC of the latter by four-fold [62].

Here, we report the synthesis and characterize novel structural analogs of **F105**, namely chiral sulfanyl and sulfonyl derivatives based on 5-(*l*)-menthyloxy- and 5-(*l*)-bornyloxy-2(5*H*)-furanones. Special interest is devoted to the evaluation of antimicrobial activity of the synthesized sulfur-containing compounds against different types of Gram-negative and Gram-positive bacteria, as well as to the effect of the combined use of 2(5*H*)-furanone derivatives with antibiotics both in vitro and in vivo.

## 2. Results and Discussion

### 2.1. Synthesis of Thioethers of 5-Menthyloxy- and 5-Bornyloxyfuranones

**Scheme 1:**
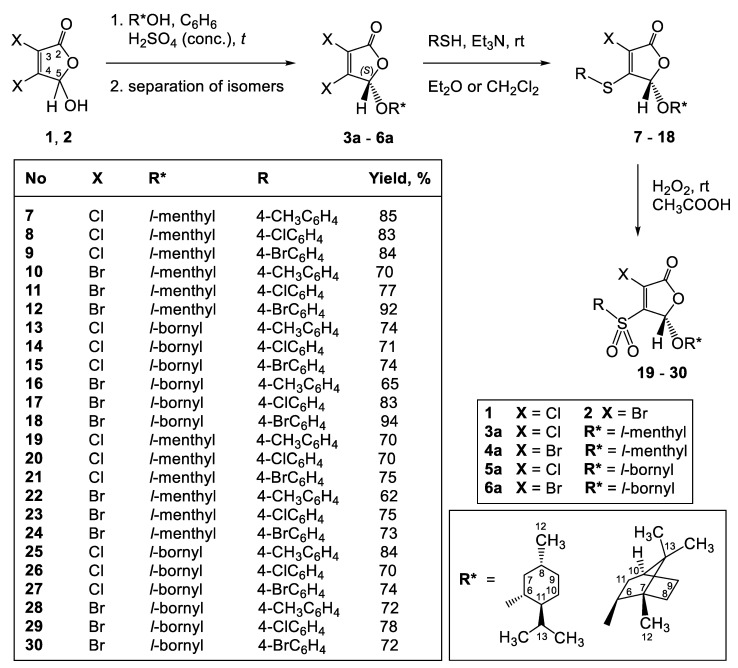
Synthesis of chiral thioethers **7**–**18** and sulfones **19**–**30** of 2(5*H*)-furanone.

**Figure 2:**
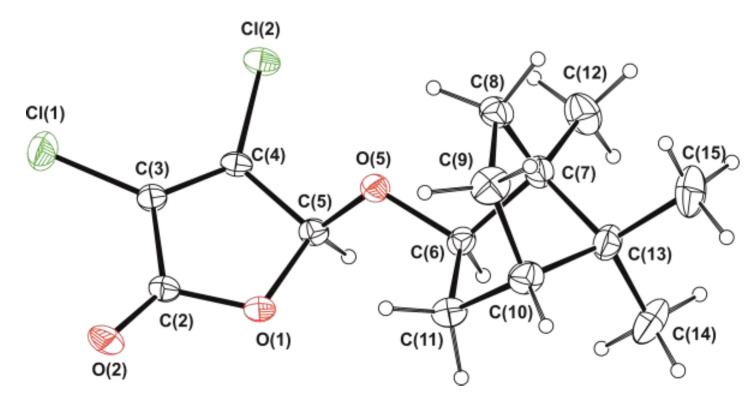
Molecular structure of 5-bornyloxy-2(5*H*)-furanone **5a** in the crystal.

In the initial step, 3,4-dihalo-2(5*H*)-furanones **3**–**6** were obtained from acid-catalyzed reactions of commercially available mucochloric **1** and mucobromic **2** acids with *l*-menthol and *l*-borneol [56,60,63,64,65,66] (Scheme 1). Compounds **3**–**6** were firstly synthesized as 1:1 mixtures of diastereomers that differ in the configuration of the C-5 atom of the γ-lactone ring. The partial recrystallization from hexane allowed for the isolating of pure less soluble (*S*)-stereoisomers **3a**–**6a**. In addition to the previously described crystal structure of 5-menthyloxy-2(5*H*)-furanone **3a** [67], we obtained X-ray diffraction data for the crystalline sample of stereoisomerically pure 5-bornyloxy-2(5*H*)-furanone **5a** and established the (*S*)-configuration of the chiral center C-5 (Figure 2).

At the next stage, aromatic thiols moieties were introduced into the molecules of (*S*)-stereoisomers **3a**–**6a** via thiolation reactions carried out under basic conditions (Scheme 1). Reactions with three different thiols were carried out at room temperature in the presence of triethylamine using the equimolar ratio of compounds **3a**–**6a**, arylthiol, and a base. The thiolation of 5-alkoxy-3,4-dihalo-2(5*H*)-furanones in the presence of base is known to proceed in a highly regioselective manner to afford 4-thiosubstituted products [68,69,70,71,72]. As a result, a series of optically pure 4-arylsulfanyl derivatives of 2(5*H*)-furanone **7**–**18** was isolated as colorless solids in good yields and characterized by HRMS, IR, and NMR spectroscopy.

The IR spectra of thioethers **7**–**18** exhibit characteristic absorption bands of C–H stretching vibrations at 2800–3000 cm^−1^, the stretching vibrations of the carbonyl group at 1755–1787 cm^−1,^ and the aromatic ring at 1474–1600 cm^−1^.

In the ^1^H NMR spectra of thioethers **7**–**18**, there were a singlet for the methine proton at carbon atom C-5 of the lactone ring (5.68–5.83 ppm) and a multiplet in the range of 7.2–7.6 ppm, characteristic for the aromatic protons. The signals of the menthol residue protons appeared as three doublets of three methyl groups in the range of 0.61–0.68, 0.83–0.87, and 0.89–0.91 ppm, a septet of doublets (1.83–1.95 ppm) arising from the methine proton of the isopropyl group, and a doublet of doublet of doublets (3.42–3.48 ppm), corresponding to the proton at the C-6. The signals of other protons of menthol moiety in the ^1^H NMR spectra of compounds **7**–**12** are presented as complex multiplets in the range of 0.7–2.2 ppm. The methylene protons at carbon atoms C-7, C-9, and C-10 of the menthol fragment are diastereotopic, and in the ^1^H NMR spectrum, each of the CH_2_ groups is observed as two individual multiplets in different regions. For example, a multiplet in the range of δ 0.74–1.13 ppm was assigned to one of the diastereotopic proton H_A_ connected to C-7, another multiplet in the range of δ 2.02–2.20 ppm was observed for another diastereotopic proton H_B_. Thus, in the ^1^H NMR spectra of compounds **7**–**12**, the magnetic nonequivalence of the diastereotopic methylene protons A and B at carbon atom C-7 is Δδ_AB_~1.1 ppm.

In the ^1^H NMR spectra of thioethers **13**–**18**, the protons of the borneol fragment are represented by three singlets caused by three methyl groups (δ 0.51–0.63, 0.72–0.79, 0.78–0.82 ppm), complex multiplets that originated from a proton at the C-10, and the diastereotopic methylene protons at carbon atoms C-8, C-9, and C-11 in the range of δ 1.0–2.3 ppm. In addition, the characteristic signal corresponding to the methine proton at the C-6 is observed at 3.5–3.8 ppm. The full assignments of the ^1^H and ^13^C NMR spectra of novel compounds **7**–**18** were made by 2D NMR techniques (^1^H–^1^H COSY, ^1^H–^13^C HSQC).

### 2.2. Synthesis of Sulfones of 5-Menthyloxy- and 5-Bornyloxyfuranones

The next stage of the synthesis required oxidation of thioethers **7**–**18** to the corresponding sulfonyl derivatives. We have previously reported the selective preparation of sulfones and sulfoxides on the basis of different mono- and dithioderivatives of 2(5*H*)-furanone [73,74,75]. A solution of hydrogen peroxide in acetic acid–a simple and highly efficient oxidizing system, was applied for the synthesis of optically active sulfones **19**–**30** from furanone thioethers **7**–**18** (Scheme 1). Reactions with an excess of H_2_O_2_ performed at room temperature resulted in the formation of desired products **19**–**30** as colorless solids.

The structure of the obtained sulfones **19**–**30** was confirmed by IR and NMR spectroscopy, while the chemical composition was proved by HRMS. The comparison of the IR spectra of thioethers **7**–**18** and oxidation products **19**–**30** showed the appearance of strong narrow peaks characteristic of the stretching vibrations of the SO_2_ group in two regions: 1342–1350 cm^−1^–antisymmetric vibrations, 1151–1163 cm^−1^–symmetric vibrations. Although the ^1^H and ^13^C{^1^H} NMR spectra of thioethers **7**–**18** and sulfones **19**–**30** have the same number of signals, a downfield shift is observed for all protons signals in the ^1^H NMR spectra of products **19**–**30**. This trend is clearly visible on the displacement of the NMR signal for the methine proton at carbon atom C-5 of the five-membered ring to the lower field (Δδ 0.4–0.5 ppm).

**Figure 3:**
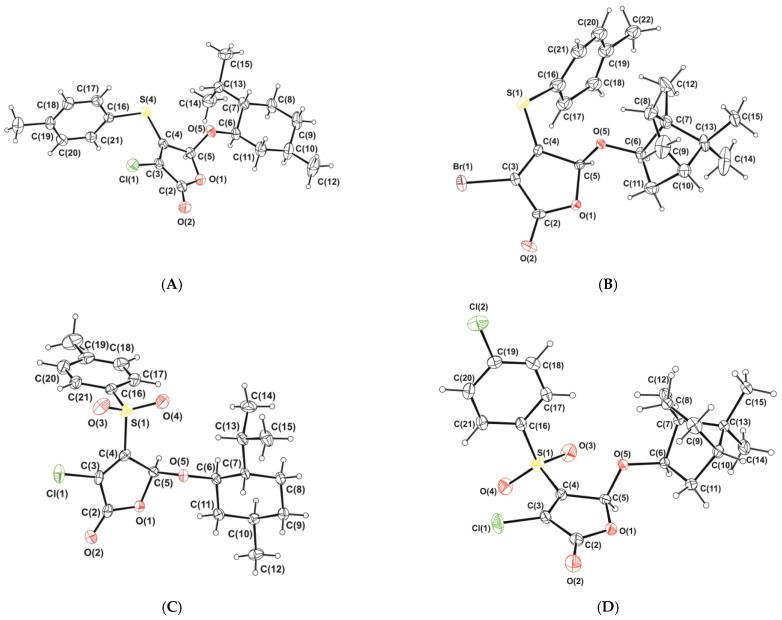
Molecular structure of thioethers **7** (**A**) and **16** (**B**), and sulfones **19** (**C**) and **26** (**D**) in the crystal.

The molecular structures of thioethers **7**, **8**, **11**, **16**, and **18** and sulfones **19**, **20**, **24**, **26**–**28**, and **30** were characterized by single crystal X-ray diffraction (Figure 3). The structures of all compounds were solved in the chiral space groups, which are characteristic of crystals of enantiopure compounds: monoclinic *P*2_1_ (compounds **8**, **11**, and **18**), trigonal *P*3_2_ (compound **16**), and orthorhombic *P*2_1_2_1_2_1_ (compounds **7**, **19**, **20**, **24**, **26**–**28**, and **30**). The absolute configuration of chiral centers has been determined from the anomalous scattering of heavy atoms (sulfur and halogens). In all of the studied molecules, both the 5-menthyloxy- and 5-bornyloxy-2(5*H*)-furanones, the C-5 carbon atom of the lactone ring has an (*S*)-configuration.

### 2.3. Antimicrobial Activity of 2(5H)-Furanone Derivatives

The antimicrobial properties of 2(5*H*)-furanone derivatives **7**–**30** were evaluated on various Gram-negative and Gram-positive bacteria. Table 1 shows the MICs of synthesized compounds in comparison with their structural analog **19** and reference antimicrobials vancomycin, benzalkonium chloride, and miramistin. The activity of all compounds was comparable to **19**, denoted as **F105**, and reported previously to repress the growth of *S. aureus* and prevent the biofilm formation by this bacterium [60,61]. Neither of novel compounds synthesized were able to repress the growth of Gram-negative bacteria. The thioethers **7**–**18** were also inactive against Gram-positive bacteria (MICs > 64 μg/mL, Table 1).

The antimicrobial activity of sulfones **20**–**30** on Gram-positive bacteria was comparable with that of **19** and less than reference antiseptics. The most active compound, **26**, containing the sulfonyl group and *l*-borneol moiety, inhibited the growth of *S. aureus* and *B. subtilis* with an MIC value of 8 μg/mL.

Since natural derivatives of 2(5*H*)-furanone were reported as biofilm-repressing agents [45,48], the ability of compounds **7**–**30** to repress the biofilm formation by various bacteria was evaluated (Table 2). All compounds inhibited the biofilm formation by Gram-positive bacteria. The biofilm-preventing concentrations (BPC) of **7**–**30** against *S. aureus*, *B. subtilis,* and *S. epidermidis* corresponded to the respective MIC values or slightly exceeded them. Therefore, we propose that the biofilm-preventing activity of compounds **7**–**30** is a consequence of bacterial cell growth repression rather than a specific targeting of biofilm formation pathways.

### 2.4. Evaluation of the Biological Activity of **26** In Vitro and In Vivo

#### 2.4.1. Bactericidal Activity of **26** against Gram-Positive Pathogens

*S. aureus* accounts for a vast majority of bacterial skin infections in humans, and an increasing resistance to existing antibiotics challenges the development of new treatment strategies [76]. Since novel sulfones of 2(5*H*)-furanone **20**–**30** exhibited antibacterial activity against *S. aureus*, we proposed that these compounds could potentially be relevant in treatment of *S. aureus*-associated skin infections. Owing to the most promising antibacterial activity of compound **26**, shown above, we further performed in-depth studies for this selected sulfone. While the pH of skin might differ from the conventional laboratory conditions of bacteria growth, we controlled a role of pH on antimicrobial activity of **26**.

We first determined the MBC of **26** against a set of various Gram-positive pathogens, including *S. aureus*, *S. epidermidis*, *Micrococcus luteus,* and *B. cereus* (Table 3). Sulfone **26** showed bactericidal activity against these bacteria with MBCs ranging from 8 to 32 μg/mL at neutral pH values (close to 7.0). However, when the pH value was decreased to 6, close to the natural pH of the skin, the MBC values substantially reduced and ranged from 2 to 8 μg/mL. With the pH increase to the alkaline range, the efficiency of sulfone **26** decreased.

#### 2.4.2. Analysis of Synergistic Interactions of **26** and Aminoglycoside Antibiotics

Combinations of antimicrobial agents are often used to increase overall antimicrobial treatment efficacy and reduce the risks of developing the antibiotic resistance. Sulfone **19** possessing *l*-menthol and sulfonyl moieties was previously shown to exhibit a synergy with amikacin, gentamicin, kanamycin, and benzalkonium chloride [60]. Therefore, the antimicrobial efficacy of sulfone **26**, in combination with aminoglycoside antibiotics (kanamycin, gentamicin and amikacin) against *S. aureus,* was further studied by the checkerboard assay. We observed the synergistic effect when **26** was combined with either gentamicin or amikacin with fractional inhibitory concentration index (FICI) values of 0.33 ± 0.16 and 0.33 ± 0.04, respectively (Table 4). Interestingly, the lower effect in combination of **26** and kanamycin was observed with FICI value of 0.44 ± 0.17.

**Figure 4:**
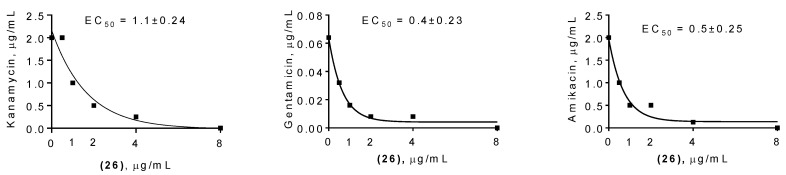
Antibacterial activity of **26** when combined with aminoglycoside antibiotics against *S. aureus*. The EC_50_ of **26** was calculated as the concentration of the antimicrobial agent that reduced two-fold MIC of an antibiotic and presented in µg/mL; mean ± SD from five calculations is shown.

Further analysis of the checkerboard assay was performed by plotting the MICs of **26** over the MICs of an aminoglycoside antibiotic (Figure 4). We calculated the EC_50_ of **26** that resulted in a two-fold decrease of MIC of solely antibiotic (Table 4). The EC_50_ values of compound **26** when combined with kanamycin, gentamicin, and amikacin were 1.1 ± 0.24 μg/mL, 0.4 ± 0.23 μg/mL and 0.5 ± 0.25 μg/mL, respectively.

#### 2.4.3. The Cytotoxicity of **26**

**Figure 5:**
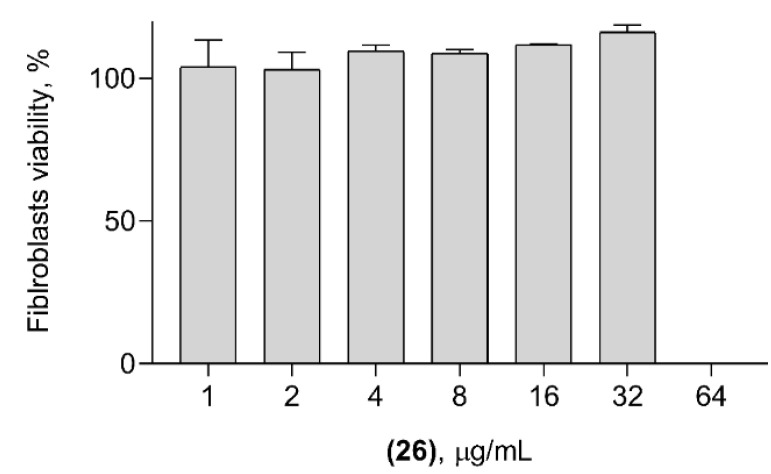
Cytotoxicity of **26** on human fibroblasts. The viability of non-treated cells has been considered as 100%; the mean ± SD from five calculations is shown.

The cytotoxic concentration of **26** on human cells in order to assess their selectivity against bacteria was further determined in the MTT assay. The sulfone **26** at a concentration of 32 μg/mL remains completely safe for human fibroblasts, while the next dilution at 64 μg/mL significantly inhibits the growth of the cells (Figure 5). The obtained cytotoxicity data are relatively similar to the data obtained earlier for the compound **19** [60], suggesting that the compound is suitable for the topical application, while systemic use could be problematic.

#### 2.4.4. Assessment of Resistance Development by *S. aureus* to **26**

**Figure 6:**
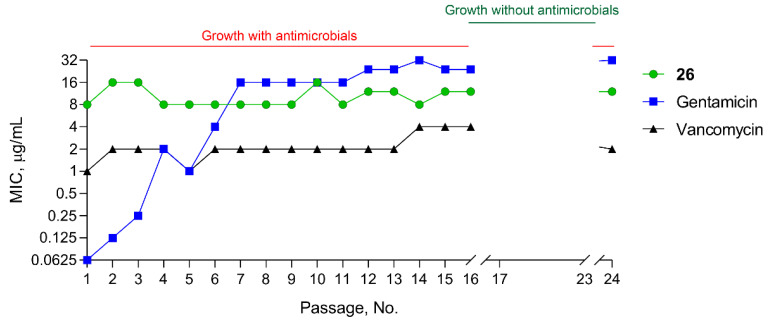
Development of *S. aureus* resistance to compound **26** (green line), gentamicin (blue line), or vancomycin (black line). Sequential passages 1 to 16 were performed in the presence of antimicrobial agents at sublethal concentrations, followed by seven passages without treatment, and the MIC was determined after each passage. The median values from five independent experiments are shown.

We previously showed a low risk of the resistance development to 2(5*H*)-furanone derivative by *B. cereus* [56], suggesting these compounds are an attractive alternative approach for the treatment of resistant bacterial strains. Therefore, we next aimed to investigate whether *S. aureus* can develop resistance to **26** compared to gentamicin and vancomycin. After 16 passages of growth in the presence of antimicrobial agents at sublethal concentrations, *S. aureus* did not significantly change susceptibility to compound **26** (Figure 6). Similarly, *S. aureus* did not develop a resistance to vancomycin with a minor increase of MIC from 1 to 2 µg/mL. Surprisingly, the MIC of gentamicin against *S. aureus* increased by more than 100-fold by the end of the experiment since a significant increase in MIC values was observed during the first seven passages from 0.06 to 16 µg/mL.

#### 2.4.5. Evaluation of Antimicrobial Activity of **26** Combined with Gentamicin on a Rat Skin Infection Model

**Figure 7:**
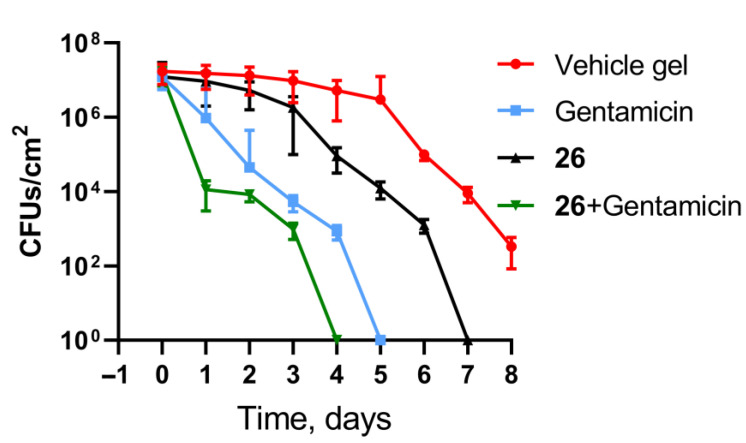
Count of *S. aureus* CFUs from the swabs of infected skin wounds of rats before and after treatment. Treatment started after 24 h of infection at day 0 of the timeline. The CFUs were counted by drop plate assay of serial dilution of bacteria from cotton swabs. The median ± IQR from five independent experiments is shown.

Owing to a low risk of resistance development and the small concentrations of **26** required for increasing the efficacy of gentamicin, we were interested in whether **26** can improve the antimicrobial activity of gentamicin on an in vivo model of an infected skin wound in a rat as described in [77]. The wounds were formed in the dorsal region of rats and infected with liquid *S. aureus* culture for one day. The 24 h application of bacterial suspension resulted in 10^7^ CFUs/cm^2,^ suggesting the infection development. Every day, the wounds were treated with a gel containing a combination of **26** and gentamicin. The solely vehicle gel or gels containing either **26** or gentamicin alone served as controls. In addition, the wounds were washed with cotton swabs to further analyze remaining *S. aureus* colony forming units (CFU). The fastest bacterial decontamination of the rat wounds was observed after four days of treatment with gentamicin combined with **26** (Figure 7). A treatment with either gentamicin or **26** alone cleared the wound from bacteria after five and seven days, respectively. A vehicle gel decreased bacterial number in the wound to a much lesser extent.

**Figure 8:**
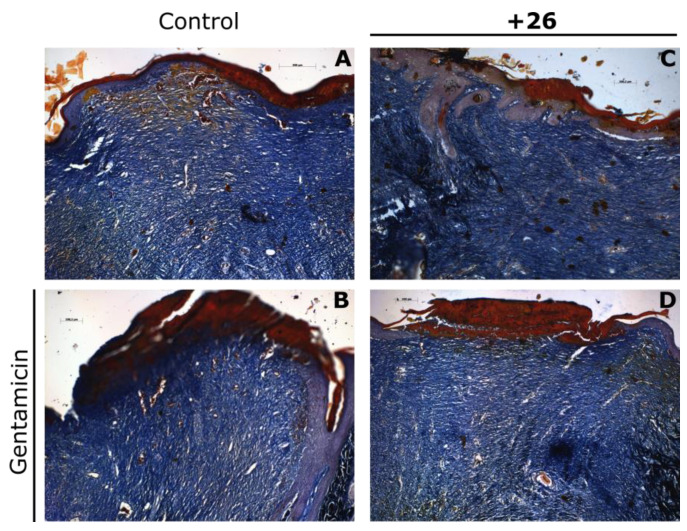
Histological analysis of rat skin areas subjected to infection and treated for 15 days with vehicle gel (**A**), gentamicin (**B**), **26** (**C**), and **26** combined with gentamicin (**D**). Scale bar is 100 µm. Samples were fixed in 10% formalin and collagen fibers were stained according to Mallory’s protocol.

One of the main signs of wound healing is the formation of collagen in the wound area, which contributes to the tissue recovery process. During wound healing, active proliferation of epithelial cells along the periphery of the formed wound and their subsequent migration towards the wound are triggered. An important role of collagen in the re-epithelialization of damaged tissue is its positive effect on the attachment and reproduction of epithelial cells. In addition, collagen is able to adhere to platelet cells, which leads to their binding to collagen molecules. This, in turn, initiates the process of isolating various factors that promote wound healing. For a detailed characterization of tissue repair, collagen fibers were stained according to the Mallory, after which the dispersion and density of collagen were analyzed (Figure 8).

**Figure 9:**
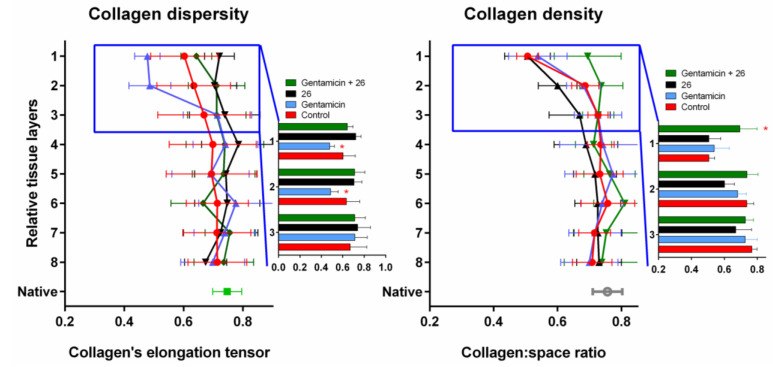
The collagen dispersity and collagen density in rat skin areas subjected to infection and treated for 15 days with **26** combined with gentamicin, solely gentamicin, solely **26**, or vehicle gel as indicated. Parameters corresponding to tissue unaffected in surgery are marked as Native. Relative tissue layers correspond to horizontal layers of the grid used for images analysis where the first layer corresponds to the top of the recovered tissue. Mean ± SD from five calculations is shown. Asterisks show significant difference with wounds treated with vehicle gel.

To assess the quality of dermal repair, the orientation and density of collagen fibers were analyzed as described previously in [77,78]. To assess the direction of collagen orientation, histological images were aligned using a 2D Cartesian grid. The average intersection length (MIL) was calculated for each element [79]; a suitable ellipse was then determined and eigenvalues and eigenvectors were calculated. The collagen elongation was evaluated using the aspect ratio of the eigenvalues. Therefore, we analyzed the distribution of the collagen elongation tensor over the relative layers of the restored tissue after 15 days of treatment (Figure 9).

In wounds treated with gentamicin only, the elongation tensor was lower in the upper layers of the wound (blue line in the graph), while in other cases, collagen recovery was roughly similar throughout all layers. Of note, the collagen’s elongation tensor has a tendency to be higher in **26**-treated samples, assuming the randomicity of fibers directions distribution compared to the control. Apparently, the combined action of **26** and gentamicin leads to faster recovering of tissues with a structure close to native ones. Moreover, the highest density of collagen in the upper layers of the tissue was observed in tissues treated with the combination of **26** and gentamicin, but the mechanism of this reduction remains unclear. Probably, the faster clearing of the wound from pathogenic microflora, as of day 4, also contributed to the rapid healing of the wound. Taken together, our data clearly show that the treatment, with the combination of **26** and gentamicin, improves bacterial decontamination and wound healing.

## 3. Materials and Methods

### 3.1. General Information

3,4-Dichloro-5-hydroxyfuran-2(5*H*)-one (mucochloric acid, **1**) (Vekton, Russia) was recrystallized from water, mp 127 °C. 3,4-Dibromo-5-hydroxyfuran-2(5*H*)-one (mucobromic acid, **2**), (1*R*,2*S*,5*R*)-(–)-menthol (*l*-menthol), (1*S*,2*R*,4*S*)-(–)-borneol (*l*-borneol), 4-chlorothiophenol (Acros Organics), 4-bromothiophenol and 4-methylthiophenol (Alfa Aesar) were used as received without further purification. All solvents were purified and distilled by standard procedures. Analytical thin layer chromatography (TLC) was carried out on Sorbfil PTLC-AF-A-UF plates using dichloromethane as the eluent and UV light (254 nm) as the visualizing agent. Silica gel 60A (Acros Organics, 70–230 mesh, 0.060–0.200 mm) was used for open column chromatography. The melting points were measured on an OptiMelt Stanford Research Systems MPA100 automated melting point apparatus and were not corrected. Optical rotations were measured on a Perkin–Elmer model 341 polarimeter at λ 589 nm and at 20 °C in chloroform (concentration *c* is given as g/100 mL). IR spectra were recorded on a Bruker Tensor-27 spectrometer fitted with a Pike MIRacle ATR accessory (diamond/ZnSe crystal plate). IR spectra were recorded of solids with characteristic absorption wavenumbers (*ν*_max_) reported in cm^−1^. NMR spectra were measured on a Bruker Avance III 400 spectrometer at 400.17 MHz (^1^H) and 100.62 MHz (^13^C) at 20 °C in the deuterated chloroform. The chemical shifts (δ) are expressed in parts per million (ppm) and are calibrated using residual undeuterated solvent peak as an internal reference (CDCl_3_: δ_H_ 7.26, δ_C_ 77.16). All coupling constants (*J*) are reported in Hertz (Hz), and multiplicities are indicated as: s (singlet), d (doublet), ddd (doublet of doublets of doublets), septd (septet of doublets), and m (multiplet). High-resolution mass spectra (HRMS) were obtained by electrospray ionisation (ESI) with positive (+) ion detection on a Bruker micrOTOF–QIII quadrupole time-of-flight mass spectrometer.

The X-ray diffraction data for the single crystals of compounds **5a**, **7**, **8**, **11**, **16**, **18**, **19**, **24**, **26**–**28**, and **30** were collected on a Bruker Smart Apex II CCD diffractometer (ω-scan mode) using graphite-monochromated Mo*Kα* (0.71073 Å) radiation at 296(2) K (**5a**, **7**, **16**, **18**, **19**, **24**, **26**–**28**, and **30**) and at 150 K (**8**, **11**). The structures were solved by the intrinsic phasing method using the SHELXT-2018/2 program [80] and refined by full-matrix least-squares on F^2^ using the SHELXL-2018/3 program [81]. Calculations were mainly performed using the WinGX-2014.1 suite of programs [82]. Non-hydrogen atoms were refined anisotropically. Hydrogen atoms were inserted at the calculated positions and refined as riding atoms. The absolute structure of the crystals and absolute configuration were determined on the basis of the Flack parameter [83,84]. All the compounds studied have no unusual bond lengths and angles. The crystal data, data collection, and structure refinement details are summarized in Table S1 (see the Supplementary Materials).

The crystallographic data for 5a, 7, 8, 11, 16, 18, 19, 24, 26–28, and 30 have been deposited in the Cambridge Crystallographic Data Centre as supplementary publication numbers CCDC 2206717, 2206714, 2206713, 2206722, 2206720, 2206716, 2206715, 2206724, 2206721, 2206723, 2206718, and 2206719, respectively. These data can be obtained free of charge via www.ccdc.cam.ac.uk/data_request/cif (accessed on 12 September 2022), or by emailing data_request@ccdc.cam.ac.uk, or by contacting The Cambridge Crystallographic Data Centre, 12 Union Road, Cambridge CB2 1EZ, UK; fax: +44-1223-336033. 

### 3.2. Chemical Synthesis

5(*S*)-3,4-Dichloro-5-[(1*R*,2*S*,5*R*)-2-isopropyl-5-methylcyclohexyloxy]-2(5*H*)-furanone (**3a**) [60,63], 5(*S*)-3,4-dibromo-5-[(1*R*,2*S*,5*R*)-2-isopropyl-5-methylcyclohexyloxy]-2(5*H*)-furanone (**4a**) [64], 5(*S*)-3,4-dichloro-5-[(1*S*,2*R*,4*S*)-1,7,7-trimethylbicyclo[2.2.1]heptan-2-yloxy]-2(5*H*)-furanone (**5a**) [56,65], 5(*S*)-3,4-dibromo-5-[(1*S*,2*R*,4*S*)-1,7,7-trimethylbicyclo[2.2.1]heptan-2-yloxy]-2(5*H*)-furanone (**6a**) [66], 5(*S*)-3-chloro-5-[(1*R*,2*S*,5*R)*-2-isopropyl-5-methylcyclohexyloxy]-4-[4-methylphenylsulfanyl]-2(5*H*)-furanone (**7**) [60], 5(*S*)-3-chloro-4-[(4-chlorophenyl)sulfanyl]-5-[(1*S*,2*R*,4*S*)-1,7,7-trimethylbicyclo[2.2.1]heptan-2-yloxy]-2(5*H*)-furanone (**14**) [85], 5(*S*)-3-chloro-5-[(1*R*,2*S*,5*R*)-2-isopropyl-5-methylcyclohexyloxy]-4-[4-methylphenylsulfonyl]-2(5*H*)-furanone (**19**) [60], and 5(*S*)-3-chloro-4-[(4-chlorophenyl)sulfonyl]-5-[(1*S*,2*R*,4*S*)-1,7,7-trimethylbicyclo[2.2.1]heptan-2-yloxy]-2(5*H*)-furanone (**26**) [85] were synthesized according to the known methods.

#### 3.2.1. General Procedure for the Synthesis of Thioethers **8**–**18**

To a solution of furanone **3a** (0.40 g, 1.3 mmol) in diethyl ether (10 mL) with intense stirring was added dropwise a solution of 4-chlorothiophenol (0.19 g, 1.3 mmol) in diethyl ether (5 mL), and a solution of triethylamine (0.18 mL, 1.3 mmol) in diethyl ether (2 mL). The reaction mixture was stirred at room temperature for 2 h (monitored by TLC), while precipitated triethylamine hydrochloride was filtered out and washed with diethyl ether. The combined filtrates were evaporated to dryness and the obtained solid residue was recrystallized from hexane to afford thioether **8**.

**5(*S*)-3-Chloro-4-[4-chlorophenylsulfanyl]-5-[(1*R*,2*S*,5*R*)-2-isopropyl-5-methylcyclohexyloxy]-2(5*H*)-furanone (8)**: colorless crystals, 0.45 g (83% yield); mp 157 °C; *R*_f_ 0.57 (CH_2_Cl_2_); [α] –103.9 (*c* 1.0, CHCl_3_); IR (ATR) ν_max_ 2960, 2949, 2923, 2881, 2871, 2854 (C–H), 1771 (C=O), 1598, 1482 (C=C aromatic ring) cm^−1^; ^1^H NMR (CDCl_3_, 400 MHz, ppm) *δ* 7.47, 7.39 (4H, AA′BB′, ^3^*J*_AB_ = ^3^*J*_A‘B’_ = 8.2 Hz, ^4^*J*_AA′_ = ^4^*J*_BB′_ = 2.3 Hz, ^5^*J*_AB′_ = ^5^*J*_A′B_ = 0.4 Hz, Ar-H), 5.83 (1H, s, H-5), 3.48 (1H, ddd, ^3^*J* = 10.7, 4.4 Hz, H-6), 2.19–2.09 (1H, m, H-7), 1.93 (1H, septd, ^3^*J* = 7.0, 2.5 Hz, H-13), 1.69–1.60 (2H, m, H-9, H-10), 1.46–1.20 (2H, m, H-11, H-8), 1.13–0.75 (3H, m, H-7, H-9, H-10), 0.91 (3H, d, ^3^*J* = 6.5 Hz, H-12), 0.87 (3H, d, ^3^*J* = 7.0 Hz, C*H*_3_ (*i*Pr)), 0.68 (3H, d, ^3^*J* = 7.0 Hz, C*H*_3_ (*i*Pr)); ^13^C{^1^H} NMR (CDCl_3_, 100 MHz, ppm) *δ* 165.0 (C-2), 152.8 (C-4), 136.7, 135.6, 129.8, 124.8, 121.3 (C-3, Ar-C), 102.0 (C-5), 83.3 (C-6), 48.1 (C-11), 42.4 (C-7), 34.0 (C-9), 31.8 (C-8), 25.2 (C-13), 22.9 (C-10), 22.2 (C-12), 21.2, 16.0 (CH_3_ (*i*Pr)); HRMS (ESI) *m/z* 437.0713 (calcd for C_20_H_24_Cl_2_NaO_3_S, 437.0715).

**5(*S*)-4-[4-Bromophenylsulfanyl]-3-chloro-5-[(1*R*,2*S*,5*R*)-2-isopropyl-5-methylcyclohexyloxy]-2(5*H*)-furanone (9)**: colorless crystals, 0.50 g (84% yield); mp 161 ^°^C; *R*_f_ 0.53 (CH_2_Cl_2_); [α] –42.4 (*c* 1.0, CHCl_3_); IR (ATR) ν_max_ 2954, 2943, 2918, 2875, 2865, 2848 (C–H), 1772 (C=O), 1594, 1475 (C=C aromatic ring) cm^−1^; ^1^H NMR (CDCl_3_, 400 MHz, ppm) *δ* 7.54, 7.39 (4H, AA′BB′, ^3^*J*_AB_ = ^3^*J*_A‘B’_ = 8.2 Hz, ^4^*J*_AA′_ = ^4^*J*_BB′_ = 2.2 Hz, ^5^*J*_AB′_ = ^5^*J*_A′B_ = 0.3 Hz, Ar-H), 5.83 (1H, s, H-5), 3.48 (1H, ddd, ^3^*J* = 10.7, 4.4 Hz, H-6), 2.20–2.08 (1H, m, H-7), 1.93 (1H, septd, ^3^*J* = 7.0, 2.6 Hz, H-13), 1.70–1.59 (2H, m, H-9, H-10), 1.44–1.32 (1H, m, H-8), 1.32–1.20 (1H, m, H-11), 1.12–0.74 (3H, m, H-7, H-9, H-10), 0.91 (3H, d, ^3^*J* = 6.5 Hz, H-12), 0.87 (3H, d, ^3^*J* = 7.0 Hz, C*H*_3_ (*i*Pr)), 0.68 (3H, d, ^3^*J* = 7.0 Hz, C*H*_3_ (*i*Pr)); ^13^C{^1^H} NMR (CDCl_3_, 100 MHz, ppm) *δ* 165.0 (C-2), 152.7 (C-4), 135.7, 132.8, 125.6, 124.8, 121.5 (C-3, Ar-C), 101.9 (C-5), 83.3 (C-6), 48.2 (C-11), 42.4 (C-7), 34.1 (C-9), 31.8 (C-8), 25.2 (C-13), 22.9 (C-10), 22.2 (C-12), 21.2, 16.0 (CH_3_ (*i*Pr)); HRMS (ESI) *m/z* 481.0208 (calcd for C_20_H_24_BrClNaO_3_S, 481.0210).

**5(*S*)-3-Bromo-4-[4-methylphenylsulfanyl]-5-[(1*R*,2*S*,5*R*)-2-isopropyl-5-methylcyclohexyloxy]-2(5*H*)-furanone (10)**: colorless crystals, 0.40 g (70% yield); mp 108–110 °C; *R*_f_ 0.57 (CH_2_Cl_2_); [α] –116.0 (*c* 1.0, CHCl_3_); IR (ATR) ν_max_ 2957, 2948, 2927, 2877, 2870, 2860 (C–H), 1760 (C=O), 1587, 1497 (C=C aromatic ring) cm^−1^; ^1^H NMR (CDCl_3_, 400 MHz, ppm) *δ* 7.41, 7.20 (4H, AA′BB′, *N* = ^3^*J*_AB_ + ^5^*J*_AB′_ = 8.2 Hz, Ar-H), 5.80 (1H, s, H-5), 3.42 (1H, ddd, ^3^*J* = 10.7, 4.3 Hz, H-6), 2.39 (3H, s, C*H*_3_), 2.11–2.02 (1H, m, H-7), 1.84 (1H, septd, ^3^*J* = 7.0, 2.4 Hz, H-13), 1.66–1.57 (2H, m, H-9, H-10), 1.41–1.29 (1H, m, H-8), 1.29–1.19 (1H, m, H-11), 1.09–0.74 (3H, m, H-7, H-9, H-10), 0.89 (3H, d, ^3^*J* = 6.5 Hz, H-12), 0.83 (3H, d, ^3^*J* = 7.0 Hz, C*H*_3_ (*i*Pr)), 0.61 (3H, d, ^3^*J* = 7.0 Hz, C*H*_3_ (*i*Pr)); ^13^C{^1^H} NMR (CDCl_3_, 100 MHz, ppm) *δ* 165.9 (C-2), 158.7 (C-4), 140.6, 134.6, 130.4, 122.9 (Ar-C), 109.2 (C-3), 102.7 (C-5), 82.6 (C-6), 48.2 (C-11), 42.3 (C-7), 34.1 (C-9), 31.8 (C-8), 25.0 (C-13), 22.9 (C-10), 22.2 (C-12), 21.5 (CH_3_), 21.3, 16.0 (CH_3_ (*i*Pr)); HRMS (ESI) *m/z* 461.0762 (calcd for C_21_H_27_BrNaO_3_S, 461.0756).

**5(*S*)-3-Bromo-4-[4-chlorophenylsulfanyl]-5-[(1*R*,2*S*,5*R*)-2-isopropyl-5-methylcyclohexyloxy]-2(5*H*)-furanone (11)**: colorless crystals, 0.46 g (77% yield); mp 134–135 °C; *R*_f_ 0.58 (CH_2_Cl_2_); [α] –84.4 (*c* 1.0, CHCl_3_); IR (ATR) ν_max_ 2953, 2942, 2918, 2876, 2865, 2847 (C–H), 1769 (C=O), 1582, 1478 (C=C aromatic ring) cm^−1^; ^1^H NMR (CDCl_3_, 400 MHz, ppm) *δ* 7.47, 7.39 (4H, AA′BB′, ^3^*J*_AB_ = ^3^*J*_A‘B’_ = 8.2 Hz, ^4^*J*_AA′_ = ^4^*J*_BB′_ = 2.1 Hz, ^5^*J*_AB′_ = ^5^*J*_A′B_ = 0.3 Hz, Ar-H), 5.80 (1H, s, H-5), 3.45 (1H, ddd, ^3^*J* = 10.7, 4.4 Hz, H-6), 2.15–2.05 (1H, m, H-7), 1.84 (1H, septd, ^3^*J* = 7.0, 2.3 Hz, H-13), 1.68–1.58 (2H, m, H-9, H-10), 1.45–1.30 (1H, m, H-8), 1.30–1.20 (1H, m, H-11), 1.12–0.74 (3H, m, H-7, H-9, H-10), 0.90 (3H, d, ^3^*J* = 6.5 Hz, H-12), 0.85 (3H, d, ^3^*J* = 7.0 Hz, C*H*_3_ (*i*Pr)), 0.65 (3H, d, ^3^*J* = 7.0 Hz, C*H*_3_ (*i*Pr)); ^13^C{^1^H} NMR (CDCl_3_, 100 MHz, ppm) *δ* 165.5 (C-2), 157.2 (C-4), 136.7, 135.7, 129.9, 125.0 (Ar-C), 110.5 (C-3), 102.8 (C-5), 82.9 (C-6), 48.1 (C-11), 42.4 (C-7), 34.0 (C-9), 31.8 (C-8), 25.1 (C-13), 22.9 (C-10), 22.2 (C-12), 21.2, 16.0 (CH_3_ (*i*Pr)); HRMS (ESI) *m/z* 481.0215 (calcd for C_20_H_24_BrClNaO_3_S, 481.0210).

**5(*S*)-3-Bromo-4-[4-bromophenylsulfanyl]-5-[(1*R*,2*S*,5*R*)-2-isopropyl-5-methylcyclohexyloxy]-2(5*H*)-furanone (12)**: colorless crystals, 0.60 g (92% yield); mp 141–142 °C; *R*_f_ 0.54 (CH_2_Cl_2_); [α] –78.6 (*c* 1.0, CHCl_3_); IR (ATR) ν_max_ 2959, 2948, 2923, 2882, 2871, 2853 (C–H), 1772 (C=O), 1586, 1479 (C=C aromatic ring) cm^−1^; ^1^H NMR (CDCl_3_, 400 MHz, ppm) *δ* 7.54, 7.39 (4H, AA′BB′, ^3^*J*_AB_ = ^3^*J*_A‘B’_ = 8.2 Hz, ^4^*J*_AA′_ = ^4^*J*_BB′_ = 2.2 Hz, ^5^*J*_AB′_ = ^5^*J*_A′B_ = 0.2 Hz, Ar-H), 5.80 (1H, s, H-5), 3.45 (1H, ddd, ^3^*J* = 10.7, 4.4 Hz, H-6), 2.14–2.05 (1H, m, H-7), 1.83 (1H, septd, ^3^*J* = 7.0, 2.1 Hz, H-13), 1.68–1.58 (2H, m, H-9, H-10), 1.43–1.29 (1H, m, H-8), 1.29–1.19 (1H, m, H-11), 1.10–0.74 (3H, m, H-7, H-9, H-10), 0.90 (3H, d, ^3^*J* = 6.5 Hz, H-12), 0.85 (3H, d, ^3^*J* = 7.0 Hz, C*H*_3_ (*i*Pr)), 0.64 (3H, d, ^3^*J* = 7.0 Hz, C*H*_3_ (*i*Pr)); ^13^C{^1^H} NMR (CDCl_3_, 100 MHz, ppm) *δ* 165.5 (C-2), 157.1 (C-4), 135.8, 132.9, 125.7, 124.8 (Ar-C), 110.7 (C-3), 102.8 (C-5), 82.9 (C-6), 48.1 (C-11), 42.4 (C-7), 34.0 (C-9), 31.8 (C-8), 25.1 (C-13), 22.9 (C-10), 22.2 (C-12), 21.3, 16.0 (CH_3_ (*i*Pr)); HRMS (ESI) *m/z* 524.9710 (calcd for C_20_H_24_Br_2_NaO_3_S, 524.9705).

**5(*S*)-3-Chloro-4-[(4-methylphenyl)sulfanyl]-5-[(1*S*,2*R*,4*S*)-1,7,7-trimethylbicyclo[2.2.1]heptan-2-yloxy]-2(5*H*)-furanone (13)**: colorless crystals, 0.38 g (74% yield); mp 90 °C; *R*_f_ 0.60 (CH_2_Cl_2_); [α] –79.1 (*c* 1.0, CHCl_3_); IR (ATR) ν_max_ 2951, 2926, 2885, 2868 (C–H), 1769 (C=O), 1645 (C=C lactone), 1588, 1484 (C=C aromatic ring) cm^−1^; ^1^H NMR (CDCl_3_, 400 MHz, ppm) *δ* 7.44, 7.21 (4H, AA′BB′, *N* = ^3^*J*_AB_ + ^5^*J*_AB′_ = 7.6 Hz, Ar-H), 5.71 (1H, s, H-5), 3.66–3.59 (1H, m, H-6), 2.38 (3H, s, C*H*_3_), 2.20–2.09 (1H, m, H-11), 1.86–1.76 (1H, m, H-8 or H-9), 1.72–1.58 (2H, m, H-8 or H-9, H-10), 1.28–1.09 (3H, m, H-8, H-9, H-11), 0.80, 0.75 (6H, s, C*H*_3_ (*i*Pr)), 0.58 (3H, s, H-12); ^13^C{^1^H} NMR (CDCl_3_, 100 MHz, ppm) *δ* 165.2 (C-2), 155.2 (C-4), 140.8, 134.8, 130.5, 122.7, 119.1 (C-3, Ar-C), 102.1 (C-5), 87.8 (C-6), 49.5 (C-13), 47.7 (C-7), 44.9 (C-10), 36.7 (C-11), 28.1, 26.6 (C-8, C-9), 21.4 (CH_3_), 19.7, 18.8 (CH_3_ (*i*Pr)), 13.5 (C-12); HRMS (ESI) *m/z* 415.1106 (calcd for C_21_H_25_ClNaO_3_S, 415.1105).

**5(*S*)-4-[(4-Bromophenyl)sulfanyl]-3-chloro-5-[(1*S*,2*R*,4*S*)-1,7,7-trimethylbicyclo[2.2.1]heptan-2-yloxy]-2(5*H*)-furanone (15)**: colorless solid, 0.44 g (74% yield); mp 120 °C; *R*_f_ 0.62 (CH_2_Cl_2_); [α] –57.4 (*c* 1.0, CHCl_3_); IR (ATR) ν_max_ 2979, 2948, 2881 (C–H), 1775 (C=O), 1600, 1475 (C=C aromatic ring) cm^−1^; ^1^H NMR (CDCl_3_, 400 MHz, ppm) *δ* 7.55, 7.43 (4H, AA′BB′, *N* = ^3^*J*_AB_ + ^5^*J*_AB′_ = 8.4 Hz, Ar-H), 5.73 (1H, s, H-5), 3.77–3.68 (1H, m, H-6), 2.24–2.12 (1H, m, H-11), 1.85–1.74 (1H, m, H-8 or H-9), 1.74–1.59 (2H, m, H-8 or H-9, H-10), 1.29–1.11 (3H, m, H-8, H-9, H-11), 0.82, 0.79 (6H, s, C*H*_3_ (*i*Pr)), 0.62 (3H, s, H-12); ^13^C{^1^H} NMR (CDCl_3_, 100 MHz, ppm) *δ* 164.8 (C-2), 153.4 (C-4), 136.0, 133.0, 125.5, 125.0, 120.6 (C-3, Ar-C), 102.2 (C-5), 88.3 (C-6), 49.6 (C-13), 47.8 (C-7), 44.9 (C-10), 36.8 (C-11), 28.1, 26.6 (C-8, C-9), 19.7, 18.8 (CH_3_ (*i*Pr)), 13.6 (C-12); HRMS (ESI) *m/z* 479.0062 (calcd for C_20_H_22_BrClNaO_3_S, 479.0054).

**5(*S*)-3-Bromo-4-[(4-methylphenyl)sulfanyl]-5-[(1*S*,2*R*,4*S*)-1,7,7-trimethylbicyclo[2.2.1]heptan-2-yloxy]-2(5*H*)-furanone (16)**: colorless crystals, 0.29 g (65% yield); mp 122–123 °C; *R*_f_ 0.61 (CH_2_Cl_2_); [α] –85.0 (*c* 1.0, CHCl_3_); IR (ATR) ν_max_ 2980, 2951, 2880 (C–H), 1775 (C=O), 1588, 1493 (C=C aromatic ring) cm^−1^; ^1^H NMR (CDCl_3_, 400 MHz, ppm) *δ* 7.44, 7.21 (4H, AA′BB′, *N* = ^3^*J*_AB_ + ^5^*J*_AB′_ = 7.9 Hz, Ar-H), 5.68 (1H, s, H-5), 3.59–3.50 (1H, m, H-6), 2.37 (3H, s, C*H*_3_), 2.16–2.05 (1H, m, H-11), 1.83–1.72 (1H, m, H-8 or H-9), 1.70–1.55 (2H, m, H-8 or H-9, H-10), 1.27–1.16 (2H, m, H-8 or H-9, H-11), 1.16–1.05 (1H, m, H-8 or H-9), 0.78, 0.72 (6H, s, C*H*_3_ (*i*Pr)), 0.51 (3H, s, H-12); ^13^C{^1^H} NMR (CDCl_3_, 100 MHz, ppm) *δ* 165.6 (C-2), 159.8 (C-4), 140.8, 134.9, 130.6, 122.9 (Ar-C), 107.8 (C-3), 103.0 (C-5), 87.5 (C-6), 49.4 (C-13), 47.7 (C-7), 44.8 (C-10), 36.6 (C-11), 28.0, 26.5 (C-8, C-9), 21.4 (CH_3_), 19.7, 18.8 (CH_3_ (*i*Pr)), 13.4 (C-12); HRMS (ESI) *m/z* 459.0599 (calcd for C_21_H_25_BrNaO_3_S, 459.0600).

**5(*S*)-3-Bromo-4-[(4-chlorophenyl)sulfanyl]-5-[(1*S*,2*R*,4*S*)-1,7,7-trimethylbicyclo[2.2.1]heptan-2-yloxy]-2(5*H*)-furanone (17)**: colorless crystals, 0.38 g (83% yield); mp 149–150 °C; *R*_f_ 0.60 (CH_2_Cl_2_); [α] –56.3 (*c* 1.0, CHCl_3_); IR (ATR) ν_max_ 2978, 2949, 2880 (C–H), 1766 (C=O), 1586, 1477 (C=C aromatic ring) cm^−1^; ^1^H NMR (CDCl_3_, 400 MHz, ppm) *δ* 7.50, 7.40 (4H, AA′BB′, *N* = ^3^*J*_AB_ + ^5^*J*_AB′_ = 8.5 Hz, Ar-H), 5.70 (1H, s, H-5), 3.70–3.62 (1H, m, H-6), 2.22–2.09 (1H, m, H-11), 1.83–1.56 (3H, m, H-8, H-9, H-10), 1.28–1.08 (3H, m, H-8, H-9, H-11), 0.80, 0.76 (6H, s, C*H*_3_ (*i*Pr)), 0.56 (3H, s, H-12); ^13^C{^1^H} NMR (CDCl_3_, 100 MHz, ppm) *δ* 165.3 (C-2), 158.1 (C-4), 136.9, 136.0, 130.1, 125.0 (Ar-C), 109.4 (C-3), 103.1 (C-5), 88.0 (C-6), 49.5 (C-13), 47.8 (C-7), 44.9 (C-10), 36.8 (C-11), 28.1, 26.6 (C-8, C-9), 19.7, 18.8 (CH_3_ (*i*Pr)), 13.5 (C-12); HRMS (ESI) *m/z* 479.0056 (calcd for C_20_H_22_BrClNaO_3_S, 479.0054).

**5(*S*)-3-Bromo-4-[(4-bromophenyl)sulfanyl]-5-[(1*S*,2*R*,4*S*)-1,7,7-trimethylbicyclo[2.2.1]heptan-2-yloxy]-2(5*H*)-furanone (18)**: colorless crystals, 0.48 g (94% yield); mp 140–141 °C; *R*_f_ 0.63 (CH_2_Cl_2_); [α] –60.2 (*c* 1.0, CHCl_3_); IR (ATR) ν_max_ 2976, 2952, 2878 (C–H), 1787, 1755 (C=O), 1591, 1474 (C=C aromatic ring) cm^−1^; ^1^H NMR (CDCl_3_, 400 MHz, ppm) *δ* 7.55, 7.43 (4H, AA′BB′, *N* = ^3^*J*_AB_ + ^5^*J*_AB′_ = 8.5 Hz, Ar-H), 5.70 (1H, s, H-5), 3.69–3.61 (1H, m, H-6), 2.21–2.10 (1H, m, H-11), 1.81–1.56 (3H, m, H-8, H-9, H-10), 1.30–1.08 (3H, m, H-8, H-9, H-11), 0.80, 0.76 (6H, s, C*H*_3_ (*i*Pr)), 0.55 (3H, s, H-12); ^13^C{^1^H} NMR (CDCl_3_, 100 MHz, ppm) *δ* 165.3 (C-2), 158.0 (C-4), 136.1, 133.1, 125.6, 125.0 (Ar-C), 109.4 (C-3), 103.1 (C-5), 88.0 (C-6), 49.5 (C-13), 47.7 (C-7), 44.9 (C-10), 36.8 (C-11), 28.0, 26.6 (C-8, C-9), 19.7, 18.8 (CH_3_ (*i*Pr)), 13.5 (C-12); HRMS (ESI) *m/z* 522.9556 (calcd for C_20_H_22_Br_2_NaO_3_S, 522.9549).

#### 3.2.2. General Procedure for the Synthesis of Sulfones **20**–**30**

To the mixture of thioether **8** (0.37 g, 0.9 mmol) and glacial acetic acid (10 mL), 33% of hydrogen peroxide (0.90 mL, 9.0 mmol) was added under stirring, and the mixture was stirred for 5–7 days at room temperature (monitored by TLC). When the reaction was complete, the mixture was evaporated to dryness, and the white solid residue was recrystallized from hexane to give sulfone **20**.

**5(*S*)-3-Chloro-4-[4-chlorophenylsulfonyl]-5-[(1*R*,2*S*,5*R*)-2-isopropyl-5-methylcyclohexyloxy]-2(5*H*)-furanone (20)**: colorless crystals, 0.28 g (70% yield); mp 131 °C; *R*_f_ 0.48 (CH_2_Cl_2_); [α] +146.5 (*c* 1.0, CHCl_3_); IR (ATR) ν_max_ 2968, 2945, 2918, 2868 (C–H), 1792 (C=O), 1618 (C=C lactone), 1578, 1477 (C=C aromatic ring), 1347 (SO_2_ asym), 1158 (SO_2_ sym) cm^−1^; ^1^H NMR (CDCl_3_, 400 MHz, ppm) *δ* 7.96, 7.59 (4H, AA′BB′, ^3^*J*_AB_ = ^3^*J*_A‘B’_ = 8.5 Hz, ^4^*J*_AA_ = ^4^*J*_BB′_ = 2.0 Hz, ^5^*J*_AB_ = ^5^*J*_A′B_ = 0.2 Hz, Ar-H), 6.29 (1H, s, H-5), 3.71 (1H, ddd, ^3^*J* = 10.7, 4.5 Hz, H-6), 2.37 (1H, septd, ^3^*J* = 7.0, 2.2 Hz, H-13), 2.20–2.10 (1H, m, H-7), 1.77–1.60 (2H, m, H-9, H-10), 1.49–1.34 (1H, m, H-8), 1.34–1.21 (1H, m, H-11), 1.13–0.74 (3H, m, H-7, H-9, H-10), 0.96 (3H, d, ^3^*J* = 7.0 Hz, C*H*_3_ (*i*Pr)), 0.93 (3H, d, ^3^*J* = 6.5 Hz, H-12), 0.85 (3H, d, ^3^*J* = 7.0 Hz, C*H*_3_ (*i*Pr)); ^13^C{^1^H} NMR (CDCl_3_, 100 MHz, ppm) *δ* 162.9 (C-2), 150.8 (C-4), 142.5, 137.0, 133.8, 130.2, 130.0 (C-3, Ar-C), 101.6 (C-5), 83.9 (C-6), 48.4 (C-11), 42.3 (C-7), 33.9 (C-9), 31.8 (C-8), 24.8 (C-13), 22.8 (C-10), 22.2 (CH_3_ (*i*Pr)), 21.3 (C-12), 15.8 (CH_3_ (*i*Pr)); HRMS (ESI) *m/z* 469.0614 (calcd for C_20_H_24_Cl_2_NaO_5_S, 469.0619).

**5(*S*)-4-[4-Bromophenylsulfonyl]-3-chloro-5-[(1*R*,2*S*,5*R*)-2-isopropyl-5-methylcyclohexyloxy]-2(5*H*)-furanone (21)**: colorless crystals, 0.33 g (75% yield); mp 135–136 °C; *R*_f_ 0.43 (CH_2_Cl_2_); [α] +117.3 (*c* 1.0, CHCl_3_); IR (ATR) ν_max_ 2971, 2950, 2940, 2923, 2873, 2855 (C–H), 1795 (C=O), 1621 (C=C lactone), 1578 (C=C aromatic ring), 1350 (SO_2_ asym), 1161 (SO_2_ sym) cm^−1^; ^1^H NMR (CDCl_3_, 400 MHz, ppm) *δ* 7.88, 7.76 (4H, AA′BB′, ^3^*J*_AB_ = ^3^*J*_A‘B’_ = 8.5 Hz, ^4^*J*_AA′_ = ^4^*J*_BB′_ = 1.8 Hz, ^5^*J*_AB′_ = ^5^*J*_A′B_ = 0.2 Hz, Ar-H), 6.29 (1H, s, H-5), 3.71 (1H, ddd, ^3^*J* = 10.7, 4.5 Hz, H-6), 2.36 (1H, septd, ^3^*J* = 7.0, 2.2 Hz, H-13), 2.22–2.11 (1H, m, H-7), 1.78–1.62 (2H, m, H-9, H-10), 1.50–1.35 (1H, m, H-8), 1.35–1.22 (1H, m, H-11), 1.14–0.74 (3H, m, H-7, H-9, H-10), 0.96 (3H, d, ^3^*J* = 7.0 Hz, C*H*_3_ (*i*Pr)), 0.92 (3H, d, ^3^*J* = 6.5 Hz, H-12), 0.86 (3H, d, ^3^*J* = 7.0 Hz, C*H*_3_ (*i*Pr)); ^13^C{^1^H} NMR (CDCl_3_, 100 MHz, ppm) *δ* 162.9 (C-2), 150.8 (C-4), 137.6, 133.9, 133.0, 131.2, 130.2 (C-3, Ar-C), 101.6 (C-5), 83.9 (C-6), 48.5 (C-11), 42.3 (C-7), 33.9 (C-9), 31.8 (C-8), 24.8 (C-13), 22.8 (C-10), 22.2 (CH_3_ (*i*Pr)), 21.3 (C-12), 15.9 (CH_3_ (*i*Pr)); HRMS (ESI) *m/z* 513.0110 (calcd for C_20_H_24_BrClNaO_5_S, 513.0109).

**5(*S*)-3-Bromo-4-[4-methylphenylsulfonyl]-5-[(1*R*,2*S*,5*R*)-2-isopropyl-5-methylcyclohexyloxy]-2(5*H*)-furanone (22)**: colorless crystals, 0.26 g (62% yield); mp 108 ^°^C; *R*_f_ 0.45 (CH_2_Cl_2_); [α] +130.4 (*c* 1.0, CHCl_3_). IR (ATR) ν_max_ 2968, 2946, 2917, 2868, 2843 (C–H), 1785 (C=O), 1609 (C=C lactone), 1597 (C=C aromatic ring), 1345 (SO_2_ asym), 1157 (SO_2_ sym) cm^−1^; ^1^H NMR (CDCl_3_, 400 MHz, ppm) *δ* 7.92, 7.39 (4H, AA′BB′, *N* = ^3^*J*_AB_ + ^5^*J*_AB′_ = 8.3 Hz, Ar-H), 6.25 (1H, s, H-5), 3.71 (1H, ddd, ^3^*J* = 10.7, 4.4 Hz, H-6), 2.47 (3H, s, CH_3_), 2.42 (1H, septd, ^3^*J* = 7.0, 2.1 Hz, H-13), 2.22–2.08 (1H, m, H-7), 1.74–1.58 (2H, m, H-9, H-10), 1.48–1.33 (1H, m, H-8), 1.33–1.22 (1H, m, H-11), 1.12–0.73 (3H, m, H-7, H-9, H-10), 0.95 (3H, d, ^3^*J* = 7.0 Hz, C*H*_3_ (*i*Pr)), 0.91 (3H, d, ^3^*J* = 6.5 Hz, H-12), 0.85 (3H, d, ^3^*J* = 7.0 Hz, C*H*_3_ (*i*Pr)); ^13^C{^1^H} NMR (CDCl_3_, 100 MHz, ppm) *δ* 164.0 (C-2), 155.8 (C-4), 146.8, 135.4, 130.1, 129.0, 123.9 (C-3, Ar-C), 102.9 (C-5), 83.7 (C-6), 48.4 (C-11), 42.3 (C-7), 34.0 (C-9), 31.8 (C-8), 24.7 (C-13), 22.8 (C-10), 22.2 (CH_3_ (*i*Pr)), 22.0 (CH_3_), 21.3 (C-12), 15.8 (CH_3_ (*i*Pr)); HRMS (ESI) *m/z* 493.0659 (calcd for C_21_H_27_BrNaO_5_S, 493.0655).

**5(*S*)-3-Bromo-4-[4-chlorophenylsulfonyl]-5-[(1*R*,2*S*,5*R*)-2-isopropyl-5-methylcyclohexyloxy]-2(5*H*)-furanone (23)**: colorless crystals, 0.33 g (75% yield); mp 135 °C; *R*_f_ 0.52 (CH_2_Cl_2_); [α] +127.6 (*c* 1.0, CHCl_3_); IR (ATR) ν_max_ 2975, 2951, 2924, 2874, 2852 (C–H), 1790 (C=O), 1611 (C=C lactone), 1581, 1481 (C=C aromatic ring), 1350 (SO_2_ asym), 1162 (SO_2_ sym) cm^−1^; ^1^H NMR (CDCl_3_, 400 MHz, ppm) *δ* 7.99, 7.59 (4H, AA′BB′, ^3^*J*_AB_ = ^3^*J*_A‘B’_ = 8.5 Hz, ^4^*J*_AA_ = ^4^*J*_BB′_ = 2.0 Hz, ^5^*J*_AB_ = ^5^*J*_A′B_ = 0.2 Hz, Ar-H), 6.26 (1H, s, H^5^), 3.72 (1H, ddd, ^3^*J* = 10.7, 4.5 Hz, H-6), 2.38 (1H, septd, ^3^*J* = 6.9, 2.2 Hz, H-13), 2.23–2.11 (1H, m, H-7), 1.77–1.63 (2H, m, H-9, H-10), 1.49–1.34 (1H, m, H-8), 1.34–1.23 (1H, m, H-11), 1.12–0.74 (3H, m, H-7, H-9, H-10), 0.96 (3H, d, ^3^*J* = 6.9 Hz, C*H*_3_ (*i*Pr)), 0.92 (3H, d, ^3^*J* = 6.5 Hz, H-12), 0.86 (3H, d, ^3^*J* = 6.9 Hz, C*H*_3_ (*i*Pr)); ^13^C{^1^H} NMR (CDCl_3_, 100 MHz, ppm) *δ* 163.7 (C-2), 155.1 (C-4), 142.4, 136.8, 130.3, 129.9, 125.0 (C-3, Ar-C), 102.8 (C-5), 83.8 (C-6), 48.5 (C-11), 42.3 (C-7), 33.9 (C-9), 31.8 (C-8), 24.8 (C-13), 22.8 (C-10), 22.2 (CH_3_ (*i*Pr)), 21.3 (C-12), 15.9 (CH_3_ (*i*Pr)); HRMS (ESI) *m/z* 513.0106 (calcd for C_20_H_24_BrClNaO_5_S, 513.0109).

**5(*S*)-3-Bromo-4-[4-bromophenylsulfonyl]-5-[(1*R*,2*S*,5*R*)-2-isopropyl-5-methylcyclohexyloxy]-2(5*H*)-furanone (24)**: colorless crystals, 0.35 g (73% yield); mp 142 °C; *R*_f_ 0.48 (CH_2_Cl_2_); [α] +134.3 (*c* 1.0, CHCl_3_); IR (ATR) ν_max_ 2950, 2923, 2873, 2851 (C–H), 1789 (C=O), 1611 (C=C lactone), 1578 (C=C aromatic ring), 1350 (SO_2_ asym), 1160 (SO_2_ sym) cm^−1^; ^1^H NMR (CDCl_3_, 400 MHz, ppm) *δ* 7.90, 7.75 (4H, AA′BB′, *N* = ^3^*J*_AB_ + ^5^*J*_AB′_ = 8.6 Hz, Ar-H), 6.26 (1H, s, H-5), 3.72 (1H, ddd, ^3^*J* = 10.7, 4.5 Hz, H-6), 2.37 (1H, septd, ^3^*J* = 7.0, 2.1 Hz, H-13), 2.20–2.11 (1H, m, H-7), 1.74–1.62 (2H, m, H-9, H-10), 1.48–1.34 (1H, m, H-8), 1.34–1.22 (1H, m, H-11), 1.10–0.74 (3H, m, H-7, H-9, H-10), 0.96 (3H, d, ^3^*J* = 7.0 Hz, C*H*_3_ (*i*Pr)), 0.92 (3H, d, ^3^*J* = 6.5 Hz, H-12), 0.85 (3H, d, ^3^*J* = 7.0 Hz, C*H*_3_ (*i*Pr)); ^13^C{^1^H} NMR (CDCl_3_, 100 MHz, ppm) *δ* 163.7 (C-2), 155.0 (C-4), 137.4, 132.9, 131.1, 130.3, 125.1 (C-3, Ar-C), 102.8 (C-5), 83.8 (C-6), 48.5 (C-11), 42.3 (C-7), 33.9 (C-9), 31.8 (C-8), 24.8 (C-13), 22.8 (C-10), 22.2 (CH_3_ (*i*Pr)), 21.3 (C-12), 15.9 (CH_3_ (*i*Pr)); HRMS (ESI) *m/z* 556.9610 (calcd for C_20_H_24_Br_2_NaO_5_S, 556.9603).

**5(*S*)-3-Chloro-4-[(4-methylphenyl)sulfonyl]-5-[(1*S*,2*R*,4*S*)-1,7,7-trimethylbicyclo[2.2.1]heptan-2-yloxy]-2(5*H*)-furanone (25)**: colorless solid, 0.26 g (84% yield); mp 149–150 °C; *R*_f_ 0.49 (CH_2_Cl_2_); [α] +103.7 (*c* 1.0, CHCl_3_); IR (ATR) ν_max_ 2986, 2951, 2922, 2882 (C–H), 1785, 1774 (C=O), 1618 (C=C lactone), 1595 (C=C aromatic ring), 1343 (SO_2_ asym), 1151 (SO_2_ sym) cm^−1^; ^1^H NMR (CDCl_3_, 400 MHz, ppm) *δ* 7.97, 7.41 (4H, AA′BB′, *N* = ^3^*J*_AB_ + ^5^*J*_AB_ = 8.1 Hz, Ar-H), 6.22 (1H, s, H-5), 4.16–4.06 (1H, m, H-6), 2.48 (3H, s, C*H*_3_), 2.33–2.21 (1H, m, H-11), 1.80–1.63 (3H, m, H-8, H-9, H-10), 1.34–1.22 (1H, m, H-8 or H-9), 1.22–1.10 (2H, m, H-8 or H-9, H-11), 1.06 (3H, s, H-12), 0.884, 0.876 (6H, s, C*H*_3_ (*i*Pr)); ^13^C{^1^H} NMR (CDCl_3_, 100 MHz, ppm) *δ* 163.2 (C-2), 151.4 (C-4), 147.0, 135.6, 133.2, 130.3, 129.0 (C-3, Ar-C), 102.8 (C-5), 90.6 (C-6), 49.9 (C-13), 47.9 (C-7), 44.9 (C-10), 36.8 (C-11), 28.2, 26.6 (C-8, C-9), 22.0 (CH_3_), 19.7, 18.9 (CH_3_ (*i*Pr)), 13.9 (C-12); HRMS (ESI) *m/z* 447.1026 (calcd for C_21_H_25_ClNaO_5_S, 447.1003).

**5(*S*)-4-[(4-Bromophenyl)sulfonyl]-3-chloro-5-[(1*S*,2*R*,4*S*)-1,7,7-trimethylbicyclo[2.2.1]heptan-2-yloxy]-2(5*H*)-furanone (27)**: colorless solid, 0.23 g (74% yield); mp 160 °C; *R*_f_ 0.45 (CH_2_Cl_2_); [α] +156.4 (*c* 1.0, CHCl_3_); IR (ATR) ν_max_ 2987, 2950, 2934, 2877 (C–H), 1790, 1780 (C=O), 1619 (C=C lactone), 1574, 1471 (C=C aromatic ring), 1347 (SO_2_ asym), 1162 (SO_2_ sym) cm^−1^; ^1^H NMR (CDCl_3_, 400 MHz, ppm) *δ* 7.94, 7.76 (4H, AA′BB′, *N* = ^3^*J*_AB_ + ^5^*J*_AB_ = 8.6 Hz, Ar-H), 6.23 (1H, s, H-5), 4.20–4.03 (1H, m, H-6), 2.36–2.18 (1H, m, H-11), 1.79–1.60 (3H, m, H-8, H-9, H-10), 1.35–1.23 (1H, m, H-8 or H-9), 1.23–1.10 (2H, m, H-8 or H-9, H-11), 1.04 (3H, s, H-12), 0.884, 0.876 (6H, s, C*H*_3_ (*i*Pr)); ^13^C{^1^H} NMR (CDCl_3_, 100 MHz, ppm) *δ* 162.8 (C-2), 150.6 (C-4), 137.5, 134.2, 133.1, 131.3, 130.3 (C-3, Ar-C), 102.7 (C-5), 90.8 (C-6), 49.9 (C-13), 48.0 (C-7), 44.9 (C-10), 36.8 (C-11), 28.2, 26.7 (C-8, C-9), 19.7, 18.9 (CH_3_ (*i*Pr)), 14.0 (C-12); HRMS (ESI) *m/z* 510.9956 (calcd for C_20_H_22_BrClNaO_5_S, 510.9952).

**5(*S*)-3-Bromo-4-[(4-methylphenyl)sulfonyl]-5-[(1*S*,2*R*,4*S*)-1,7,7-trimethylbicyclo[2.2.1]heptan-2-yloxy]-2(5*H*)-furanone (28)**: colorless crystals, 0.22 g (72% yield); mp 140–141 °C; *R*_f_ 0.41 (CH_2_Cl_2_); [α] +155.6 (*c* 1.0, CHCl_3_); IR (ATR) ν_max_ 2990, 2974, 2955, 2927, 2889 (C–H), 1781 (C=O), 1614 (C=C lactone), 1594, 1482 (C=C aromatic ring), 1342 (SO_2_ asym), 1163 (SO_2_ sym) cm^−1^; ^1^H NMR (CDCl_3_, 400 MHz, ppm) *δ* 8.00, 7.40 (4H, AA′BB′, *N* = ^3^*J*_AB_ + ^5^*J*_AB′_ = 8.2 Hz, Ar-H), 6.19 (1H, s, H-5), 4.22–4.04 (1H, m, H-6), 2.48 (3H, s, C*H*_3_), 2.34–2.20 (1H, m, H-11), 1.83–1.60 (3H, m, H-8, H-9, H-10), 1.36–1.23 (1H, m, H-8 or H-9), 1.23–1.11 (2H, m, H-8 or H-9, H-11), 1.06 (3H, s, H-12), 0.89, 0.88 (6H, s, C*H*_3_ (*i*Pr)); ^13^C{^1^H} NMR (CDCl_3_, 100 MHz, ppm) *δ* 163.9 (C-2), 155.6 (C-4), 146.9, 135.4, 130.2, 129.2, 124.3 (C-3, Ar-C), 104.0 (C-5), 90.5 (C-6), 49.9 (C-13), 47.9 (C-7), 44.9 (C-10), 36.8 (C-11), 28.2, 26.7 (C-8, C-9), 22.0 (CH_3_), 19.7, 18.9 (CH_3_ (*i*Pr)), 13.9 (C-12); HRMS (ESI) *m/z* 491.0498 (calcd for C_21_H_25_BrNaO_5_S, 491.0498).

**5(*S*)-3-Bromo-4-[(4-chlorophenyl)sulfonyl]-5-[(1*S*,2*R*,4*S*)-1,7,7-trimethylbicyclo[2.2.1]heptan-2-yloxy]-2(5*H*)-furanone (29)**: colorless crystals, 0.24 g (78% yield); mp 169–170 ^°^C; *R*_f_ 0.49 (CH_2_Cl_2_); [α] +166.3 (*c* 1.0, CHCl_3_); IR (ATR) ν_max_ 3026, 2992, 2970, 2955, 2888 (C–H), 1780, 1739 (C=O), 1615 (C=C lactone), 1572, 1475 (C=C aromatic ring), 1344 (SO_2_ asym), 1162 (SO_2_ sym) cm^−1^; ^1^H NMR (CDCl_3_, 400 MHz, ppm) *δ* 8.06, 7.59 (4H, AA′BB′, *N* = ^3^*J*_AB_ + ^5^*J*_AB′_ = 8.6 Hz, Ar-H), 6.20 (1H, s, H-5), 4.20–4.08 (1H, m, H-6), 2.35–2.21 (1H, m, H-11), 1.79–1.64 (3H, m, H-8, H-9, H-10), 1.36–1.24 (1H, m, H-8 or H-9), 1.24–1.11 (2H, m, H-8 or H-9, H-11), 1.05 (3H, s, H-12), 0.89, 0.88 (6H, s, C*H*_3_ (*i*Pr)); ^13^C{^1^H} NMR (CDCl_3_, 100 MHz, ppm) *δ* 163.6 (C-2), 154.9 (C-4), 142.5, 136.8, 130.5, 130.0, 125.3 (C-3, Ar-C), 103.9 (C-5), 90.7 (C-6), 49.9 (C-13), 48.0 (C-7), 44.9 (C-10), 36.8 (C-11), 28.2, 26.7 (C-8, C-9), 19.7, 18.9 (CH_3_ (*i*Pr)), 14.0 (C-12); HRMS (ESI) *m/z* 510.9950 (calcd for C_20_H_22_BrClNaO_5_S, 510.9952).

**5(*S*)-3-Bromo-4-[(4-bromophenyl)sulfonyl]-5-[(1*S*,2*R*,4*S*)-1,7,7-trimethylbicyclo[2.2.1]heptan-2-yloxy]-2(5*H*)-furanone (30)**: colorless crystals, 0.22 g (72% yield); mp 158–160 °C; *R*_f_ 0.51 (CH_2_Cl_2_); [α] +156.9 (*c* 1.0, CHCl_3_); IR (ATR) ν_max_ 2990, 2955, 2925, 2870 (C–H), 1781 (C=O), 1614 (C=C lactone), 1569, 1470 (C=C aromatic ring), 1343 (SO_2_ asym), 1161 (SO_2_ sym) cm^−1^; ^1^H NMR (CDCl_3_, 400 MHz, ppm) *δ* 7.98, 7.76 (4H, AA′BB′, *N* = ^3^*J*_AB_ + ^5^*J*_AB′_ = 8.6 Hz, Ar-H), 6.20 (1H, s, H-5), 4.17–4.07 (1H, m, H-6), 2.34–2.21 (1H, m, H-11), 1.78–1.62 (3H, m, H-8, H-9, H-10), 1.37–1.24 (1H, m, H-8 or H-9), 1.24–1.11 (2H, m, H-8 or H-9, H-11), 1.04 (3H, s, H-12), 0.89, 0.88 (6H, s, C*H*_3_ (*i*Pr)); ^13^C{^1^H} NMR (CDCl_3_, 100 MHz, ppm) *δ* 163.6 (C-2), 154.9 (C-4), 137.3, 133.0, 131.2, 130.5, 125.4 (C-3, Ar-C), 103.9 (C-5), 90.8 (C-6), 49.9 (C-13), 48.0 (C-7), 44.9 (C-10), 36.8 (C-11), 28.2, 26.7 (C-8, C-9), 19.7, 18.9 (CH_3_ (*i*Pr)), 14.0 (C-12); HRMS (ESI) *m/z* 554.9487 (calcd for C_20_H_22_Br_2_NaO_5_S, 554.9447).

### 3.3. Strains and Growth Conditions

*Staphylococcus aureus* ATCC 29213, *Bacillus subtilis* 168, *Escherichia coli* ATCC 25922, *Pseudomonas aeruginosa* ATCC 27853, clinical isolates of *Staphylococcus epidermidis*, *Klebsiella pneumoniae*, *Micrococcus luteus*, and *Bacillus cereus* (obtained from the Kazan Institute of Epidemiology and Microbiology, Kazan, Russia) were used in this study. Bacterial strains were stored as a 50% glycerol stock and incubated in Mueller–Hinton broth (Carl Roth GmbH, Karlsruhe, Germany).

### 3.4. Determination of Minimum Inhibitory Concentration (MIC)

Determination of the minimum inhibitory concentration (MIC) of antimicrobials was carried out using the method of two-fold serial dilutions in 96-well plates (Eppendorf) in accordance with the EUCAST recommendations for testing antimicrobial susceptibility [86] with minor modifications to take into account reduced solubility of 2(5*H*)-furanone derivatives. A bacterial suspension containing 10^8^ CFU/mL was diluted in a ratio of 1:300 with Mueller–Hinton broth (Carl Roth GmbH, Germany) to obtain a suspension with a concentration of 2–8 × 10^5^ CFU/mL, after a series of two-fold dilutions of the studied antimicrobial compounds were added into the wells in concentrations from 1 to 128 µg/mL. Then, the plates were incubated at 37 °C for 20 hours and cell viability was evaluated by alamar blue test. The minimum inhibitory concentration was defined as the lowest concentration of a compound at which no bacterial growth was observed after 20 h of incubation.

### 3.5. Determination of Biofilm-Preventing Concentration (BPC)

Quantitative analysis of bacterial biofilms was performed by crystal violet staining as described in [87] with modifications [88]. The bacterial culture, diluted to a concentration of 2–8 × 10^5^ CFU/mL in Mueller–Hinton broth, was seeded in 24-well culture plates (Eppendorf). The compounds were added to plates in double serial dilutions to obtain final concentrations in the wells from 1 μg/mL to 128 μg/mL, and bacteria were grown under static conditions at 37 °C for 24 h. To stain bacterial biofilms, the liquid culture was removed from the plates and the wells were washed with phosphate-buffered saline, after which the plates were dried at room temperature overnight. Then, 1 mL of a 0.5% solution of crystal violet (Sigma-Aldrich, St. Louis, MO, USA) dissolved in 96% ethanol was added to the wells, followed by a 20-minute incubation. Then, the crystal–violet solution was removed from the wells and the plate was washed with PBS 3–4 times. After drying for 1 h, 1 mL of 96% ethanol was added to the wells to elute the dye bound to the biofilm, and the optical density was measured at a wavelength of 570 nm on Infinite 200 Pro (Tecan, Männedorf, Switzerland) microplate spectrophotometer. Wells incubated with a pure growth medium and subjected to all staining manipulations served as a control. The biofilm-preventing concentration was defined as the lowest concentration of a compound providing 50% reduction in total biofilm mass.

### 3.6. Evaluation of Cytotoxicity

The effect of the compounds on the respiratory activity of human fibroblasts was assessed using the metabolic MTT test [89]. For that, the cells were incubated in the presence of compounds for 24 h at 37 °C, in a 5% CO_2_ atmosphere. After that, an equal volume of MTT solution at a final concentration of 1 mg/mL was added to 100 μL of cell culture, and incubation continued for 2–4 h. Then, the plate was centrifuged for 5 min at 3.5 rpm to precipitate the crystals of formazan. The supernatant was removed, and the formed formazan crystals were dissolved in dimethyl sulfoxide (DMSO) for 15 min at 33 °C. Then, the absorbance was measured at a wavelength of 550 nm on a Tecan infinite 200 Pro microplate reader (Tecan, Switzerland).

### 3.7. Assessment of Synergy between Furanone and Antibiotics

To assess a synergy between furanones derivatives and gentamicin, a checkerboard assay was performed as described in [60]. The final concentrations of both compounds ranged from 1/16 to 4×MIC for a furanone derivative and from 1/256 to 4×MIC for the antibiotic. In total, nine dilution steps of antimicrobial and seven dilution steps of furanone in Mueller–Hinton broth were obtained. The plates were incubated at 37 °C for 24 h under static conditions. Each test was performed in triplicate and included a growth control without addition of any antimicrobials. Then, the effective concentrations (EC_50_) of these compounds leading to double reduce of antibiotic’s MIC were calculated by using GraphPad Prism version 6.0 for Windows (GraphPad Software, USA, www.graphpad.com, accessed on 02 January 2023).

To assess the type of interactions of two antimicrobial agents and, the fractional inhibitory concentration index (FICI) was calculated according to [90]. The obtained FICI values < 0.5 indicated synergism, 0.5 ≤ FICI ≤ 4 no effect, and FICI > 4 indicated antagonism.

### 3.8. Resistance Development

The resistance development was assessed as described in [56]. Compound **26**, or the antibiotic gentamicin, was used to study the development of resistance in *S. aureus*. The conventional antimicrobials (vancomycin and gentamicin) were used as a reference drug. Cells were grown in the presence of two-fold dilutions of compounds (from 1 to 1024 μg/mL). Cells from the cell with highest concentration where the growth was observed served as inoculum for the next cycle. The procedure was repeated to obtain 16 passages, after each of which the MIC values for the antimicrobial agent were determined. After that, a series of seven passages was carried out in petri dishes on nutrient agar in the absence of the studied antimicrobial agents, and then the MICs were again determined (passage 17).

### 3.9. Animals, Wounds Creation and Wound-Healing Assays

The experimental design was similar to previous work [77]. The study on laboratory animals was carried out on white rats, Wistar line, males, and females (SCBMT FMBA of Russia), 3–6 months old, weight 250–300 g., in accordance with the relevant regulations in Helsinki and performed in compliance with the bioethical standards and were approved by the Local ethics committee at Kazan Federal University (according to the protocol No. 14 approved on 08.02.2019).

Animal conditions: Wistar rats were kept in rooms with controlled microclimate parameters (temperature 22–26 °C, relative humidity 30–70%, air exchange 8–10 room volumes per hour, day/night light mode). The adaptation period of the animals was carried out in a vivarium with comfortable conditions (temperature 22 °C, 12-h light regime). The rats had free access to water and standard vivarium food, which they received ad libitum.

To obtain the model of infected wound the animals were anesthetized (inhalation anesthesia, isoflurane (Baxter, Deerfield, Illinois, USA); induction–3–4%, 2 min, 1 L min^–1^, basic–1–2%) [91] and a skin area of 4 × 4 cm^2^ was shaved on the back of rats. The full-thickness skin excision rounded wound of 1 cm in diameter was created using a sterile lancet in the paravertebral area of rats. Then, 100 µL of *S. aureus* ATCC 29213 suspension containing 10^9^ CFUs mL^–1^ in 0.9% saline was dropped onto the wound, and the wound was dressed. After 24 h, swabs were taken from each infected area into sterile tubes containing 1 mL of sterile 0.9% saline, and CFUs were counted with drop plate assay on salt-mannitol agar plates. In average, 10^6^–10^7^ CFUs cm^−1^ was observed, suggesting the development of infection [92].

### 3.10. Preparing Gels with Antimicrobial Compounds

Antimicrobials were applied in the form of the gel prepared on the base of hydroxypropylmethylcellulose, the synthetic cellulose ether (HPMC). This gel-forming agent is widely used to create various pharmacological preparations of gels in ophthalmology or dermatology since it is resistant to long-term storage, is soluble in water, and has viscosity at wide range of pH (4.0–9.0). The final concentrations of gentamicin and **26** were of 100× of effective concentrations (0.5 mg/mL and 0.04 mg/mL, respectively). Briefly, 50 mg of gentamicin were dissolved in 98 g of pure water, 2 g of HPMC (powder) were added, and the solution was left to swell on water for 1 hour, followed by stirring at 60–100 rpm until a homogeneous mass was formed. Alternatively, 4 mg of **26** were dissolved in 10 g of DMSO and added to 88 g of water, and 2 g of HPMC (powder) were added. To obtain the gel containing both gentamicin and **26**, 50 mg of gentamicin were dissolved in 98 g of pure water, 2 g of HPMC (powder) were added, and the solution was left to swell on water for 1 h followed with stirring at 60–100 rpm until a homogeneous mass was formed. Next, 4 mg of **26** were dissolved in 10 g of DMSO and added to obtained mass and stirred at 60–100 rpm until a homogeneous mass was formed.

### 3.11. Animals Grouping and Treatment Options

Treatment started 24 h after the infection was induced. In each experiment, eight animals (four male and four female) were selected for each experimental group. Groups were formed by random selection using body weight as a leading feature (the spread in the initial mass between and within groups did not exceed ± 20%). In the control group, the wounds were treated once per day with vehicle gel. Other groups were topically treated once per day with gel containing either gentamicin (0.5 mg/mL), solely **26** (0.04 mg/mL), or both gentamicin and **26** (0.5 and 0.04 mg/mL, respectively).

### 3.12. Evaluation of Wounds Healing and Microbial Decontamination

The experimental design was similar as described in [77]. For general anesthesia, combined intramuscular anesthesia was used using drugs such as zoletil (Zoletil 100, Virbac, Carros, France) at a concentration of 15 mg/kg, as well as injectable xylavet (XylaVET, Pharmamagist Ltd., Budapest, Hungary) at a concentration of 0.15 mL/kg. Two drugs were mixed in a syringe 1:1. Narcosis was verified by inhibition of reflex reactions. To apply plane wounds, the hair and undercoat in the interscapular region were cut off and a skin flap of 10 mm in diameter with an area of 80 mm^2^ was cut out using a special stencil. Infection was carried out by applying a bacterial suspension containing 2–3 × 10^8^ CFU of *S. aureus* ATCC29213 for 24 h. Treatment of wounds was carried out after taking a wash with cotton swabs for quantifying bacterial CFUs. After the end of the experiments, all animals were removed from the experiment by killing through the guillotine with preliminary anesthesia (zoletil, xilavet). On the 15th day of the experiment, skin areas were taken, including the entire wound surface, as well as surrounding healthy tissues.

On 15th day, animals from each group were sacrificed and the skin samples were surgically removed from the wounds and fixed 24 h in 10% formalin solution in PBS. Skin biopsy specimens included the epidermis, the dermis, and the subcutaneous panniculus carnosus muscle. Then, 10 m tissue sections were cut with a microtome (Thermo Scientific HM325, Waltham, MA, USA) to obtain the 5 µm-thick cuts, dehydrated and stained, according to Mallory protocol. The histological samples were documented on Carl Zeiss Axio Imager 2 microscope with magnification of 100× in at least 10 fields per sample. For a detailed characterization of tissue repair, the dispersion and density of collagen were analyzed. To assess the quality of dermal repair, the orientation and density of collagen fibers were analyzed as described previously in [78]. To assess the direction of collagen orientation, histological images were aligned using a 2D Cartesian grid. The average intersection length (MIL) was calculated for each element [79], followed by finding a suitable ellipse and calculating eigenvalues and eigenvectors. The collagen elongation was evaluated using the aspect ratio of the eigenvalues.

## 4. Conclusions

In conclusion, we have developed an efficient method for the synthesis of chiral 4-arylsulfanyl 2(5*H*)-furanone derivatives and their oxidation products from commercially available mucohalic acids. Novel furanone thioethers were obtained via thiolation reactions of stereochemically pure 5-(*l*)-menthyloxy- and 5-(*l*)-bornyloxy-2(5*H*)-furanones in the presence of triethylamine. The corresponding chiral sulfones, possessing arylsulfonyl group at the 4 position of the unsaturated γ-lactone ring, were synthesized using hydrogen peroxide in acetic acid. The structure of novel sulfanyl and sulfonyl derivatives of 2(5*H*)-furanone was characterized by spectral methods, HRMS, and single crystal X-ray diffraction. While thioethers **7**–**18** demonstrate no antimicrobial activity, the 4-arylsulfonyl-2(5*H*)-furanones **19**–**30** repress the growth of Gram-positive bacteria at 8–32 μg/mL. The leading compound, **26** (5(*S*)-3-chloro-4-[(4-chlorophenyl)sulfonyl]-5-[(1*S*,2*R*,4*S*)-1,7,7-trimethylbicyclo[2.2.1]heptan-2-yloxy]-2(5*H*)-furanone), was active at 8 μg/mL and significantly reduced the bacterial decontamination and healing of infected skin wounds on rats. Furthermore, sulfones **20**–**23** and **25** also increased the efficacy of gentamicin against *S. aureus* cells with EC_50_ of 0.5–1.6 μg/mL. While the relatively high cytotoxicity of compounds (specificity index CC_50_: MIC is about 6) limits their systemic use, these compounds or compounds of similar structure can be used for the topical application or for antimicrobial treatment of various surfaces in order to improve the activity of conventional antiseptics.

## Figures and Tables

**Table 1:** In vitro antimicrobial activity of 2(5*H*)-furanone derivatives **7**–**30** and reference drugs (MICs, μg/mL). The median values from five independent experiments are shown.

Compounds	Gram-Positive			Gram-Negative		
	*S. aureus* ATCC 29213	*B. subtilis* 168	*S. epidermidis* (Clinical Isolate)	*E. coli* ATCC 25922	*K. pneumonia* (Clinical Isolate)	*P. aeruginosa* ATCC 27853
**7**	>64	>64	>64	>64	>64	>64
**8**	>64	>64	>64	>64	>64	>64
**9**	>64	>64	>64	>64	>64	>64
**10**	>64	>64	>64	>64	>64	>64
**11**	>64	>64	>64	>64	>64	>64
**12**	>64	>64	>64	>64	>64	>64
**13**	>64	>64	>64	>64	>64	>64
**14**	>64	>64	>64	>64	>64	>64
**15**	>64	>64	>64	>64	>64	>64
**16**	>64	>64	>64	>64	>64	>64
**17**	>64	>64	>64	>64	>64	>64
**18**	>64	>64	>64	>64	>64	>64
**19**	16	16	16	>64	>64	>64
**20**	16	16	16	>64	>64	>64
**21**	32	32	16	>64	>64	>64
**22**	32	32	32	>64	>64	>64
**23**	32	16	32	>64	>64	>64
**24**	32	16	32	>64	>64	>64
**25**	16	32	16	>64	>64	>64
**26**	8	8	16	>64	>64	>64
**27**	16	16	16	>64	>64	>64
**28**	16	16	16	>64	>64	>64
**29**	16	16	16	>64	>64	>64
**30**	16	16	16	>64	>64	>64
Vancomycin	2	0.5	4	>64	>64	64
Benzalkonium chloride	4	8	4	>64	>64	>64
Miramistin	2	1	4	>64	>64	64

**Table 2:** In vitro biofilm-preventing activity of 2(5*H*)-furanone derivatives **7**–**30** (BPCs, μg/mL). The median values from five independent experiments are shown.

Compounds	Gram-Positive			Gram-Negative		
	*S. aureus* ATCC 29213	*B. subtilis* 168	*S. epidermidis* (Clinical Isolate)	*E. coli* ATCC 25922	*K. pneumonia* (Clinical Isolate)	*P. aeruginosa* ATCC 27853
**7**	>64	>64	>64	>64	>64	>64
**8**	>64	>64	>64	>64	>64	>64
**9**	>64	>64	>64	>64	>64	>64
**10**	>64	>64	>64	>64	>64	>64
**11**	>64	>64	>64	>64	>64	>64
**12**	>64	>64	>64	>64	>64	>64
**13**	>64	>64	>64	>64	>64	>64
**14**	>64	>64	>64	>64	>64	>64
**15**	>64	>64	>64	>64	>64	>64
**16**	>64	>64	>64	>64	>64	>64
**17**	>64	>64	>64	>64	>64	>64
**18**	>64	>64	>64	>64	>64	>64
**19**	32	32	32	>64	>64	>64
**20**	32	>64	32	>64	>64	>64
**21**	32	>64	64	>64	>64	>64
**22**	32	32	32	>64	>64	>64
**23**	32	32	16	>64	>64	>64
**24**	32	32	16	>64	>64	>64
**25**	32	32	16	>64	>64	>64
**26**	16	16	16	>64	>64	>64
**27**	16	16	16	>64	>64	>64
**28**	32	32	32	>64	>64	>64
**29**	16	16	16	>64	>64	>64
**30**	32	32	32	>64	>64	>64

**Table 3:** Bactericidal activity of compound **26** against Gram-positive bacteria *S. aureus*, *S. epidermidis*, *M. luteus,* and *B. cereus* at various pH conditions. The values indicate MBC in μg/mL; nd—no visible growth in control sample. The median values from five independent experiments are shown.

Bacteria	pH of the Medium						
	4	5	6	7	8	9	10
*S. aureus*	nd	0.5	8	32	64	128	128
*S. epidermidis*	nd	nd	2	8	32	nd	nd
*M. luteus*	nd	1	4	16	64	128	>128
*B. cereus*	nd	<0.25	8	16	64	>128	nd

**Table 4:** Antibacterial activity of aminoglycoside antibiotics in combination with **26** against *S. aureus*. MIC values indicate inhibitory activity of antimicrobials alone. The median values from five independent experiments are shown. FICI values reflect interaction type of an antibiotic in combination with **26**. EC_50_ is an effective concentration of **26** that reduces MIC of the respective antibiotics two-fold; mean ± SD from five calculations is shown.

	MIC, μg/mL	FICI	EC_50_, μg/mL
**26**	8	–	–
Kanamycin	2	0.44 ± 0.17	1.1 ± 0.24
Gentamicin	0.06	0.33 ± 0.16	0.4 ± 0.23
Amikacin	2	0.33 ± 0.04	0.5 ± 0.25

## Data Availability

All data are included in the manuscript and the Supplementary Materials.

## Supplementary Materials

The following supporting information can be downloaded at: https://www.mdpi.com/article/10.3390/molecules28062543/s1, IR, ^1^H, ^13^C{^1^H} NMR spectra, HRMS, data of the single crystal X-ray diffraction.

## Sample Availability

Samples of the compounds **3**–**30** are available from the authors.
