# Supplementary material for: The Novel Chiral 2(5H)-Furanone Sulfones Possessing Terpene Moiety: Synthesis and Biological Activity
Source: Molecules. 2023 Mar 10;28(6):2543. doi: 10.3390/molecules28062543 (PMC10057426; doi:10.3390/molecules28062543)
Supplement: Supplementary file 1 [file molecules-28-02543-s001.zip › molecules-2228463-supplementary.pdf]

Supplementary Materials

The novel chiral 2(5*H*)-furanone sulfones possessing terpene moiety: synthesis and biological activity

Alsu M. Khabibrakhmanova [1], Roza G. Faizova [1], Olga A. Lodochnikova [1,2], Regina R. Zamalieva [1], Liliya Z. Latypova [1], Elena Y. Trizna [3], Andrey G. Porfiryev [3], Katsunori Tanaka [4,5], Oskar A. Sachenkov[6], Airat R. Kayumov [3] and Almira R. Kurbangalieva [1,*]

[1] Biofunctional Chemistry Laboratory, A. Butlerov Institute of Chemistry, Kazan Federal University, 18 Kremlyovskaya Street, 420008 Kazan, Russia
[2] Arbuzov Institute of Organic and Physical Chemistry, FRC Kazan Scientific Center of RAS, 8 Arbuzov Street, 420088 Kazan, Russia
[3] Institute of Fundamental Medicine and Biology, Kazan Federal University, 18 Kremlyovskaya Street, 420008 Kazan, Russia
[4] Biofunctional Synthetic Chemistry Laboratory, RIKEN Cluster for Pioneering Research, 2-1 Hirosawa, Wako-shi, Saitama 351-0198, Japan
[5] Department of Chemical Science and Engineering, School of Materials and Chemical Technology, Tokyo Institute of Technology, 2-12-1 O-okayama, Meguro-ku, Tokyo 152-8552, Japan
[6] N.I. Lobachevsky Institute of Mathematics and Mechanics, Kazan Federal University, 18 Kremlyovskaya Street, 420008 Kazan, Russia
* Correspondence: akurbang@kpfu.ru; Tel.: +7-843-233-74-62

^{1}H and $^{13}C\{^{1}H\}$ NMR spectra of thioethers

a)

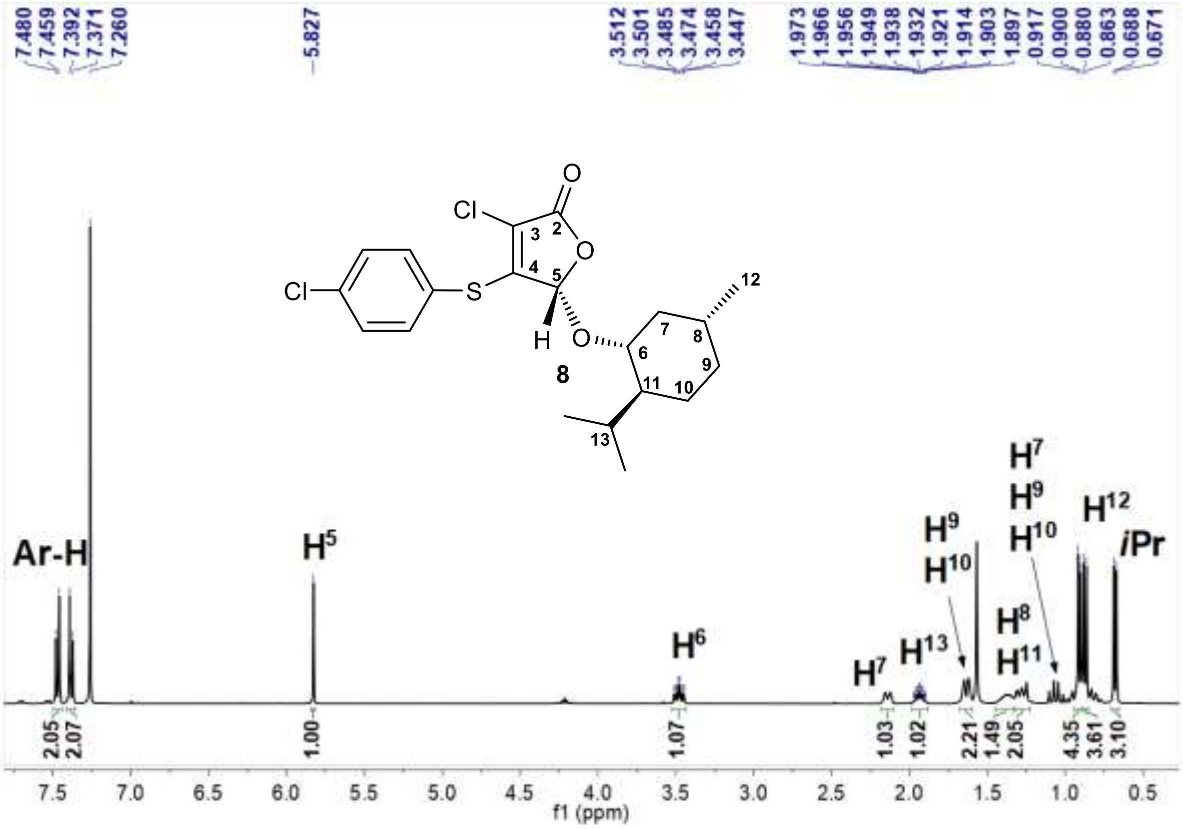

b)

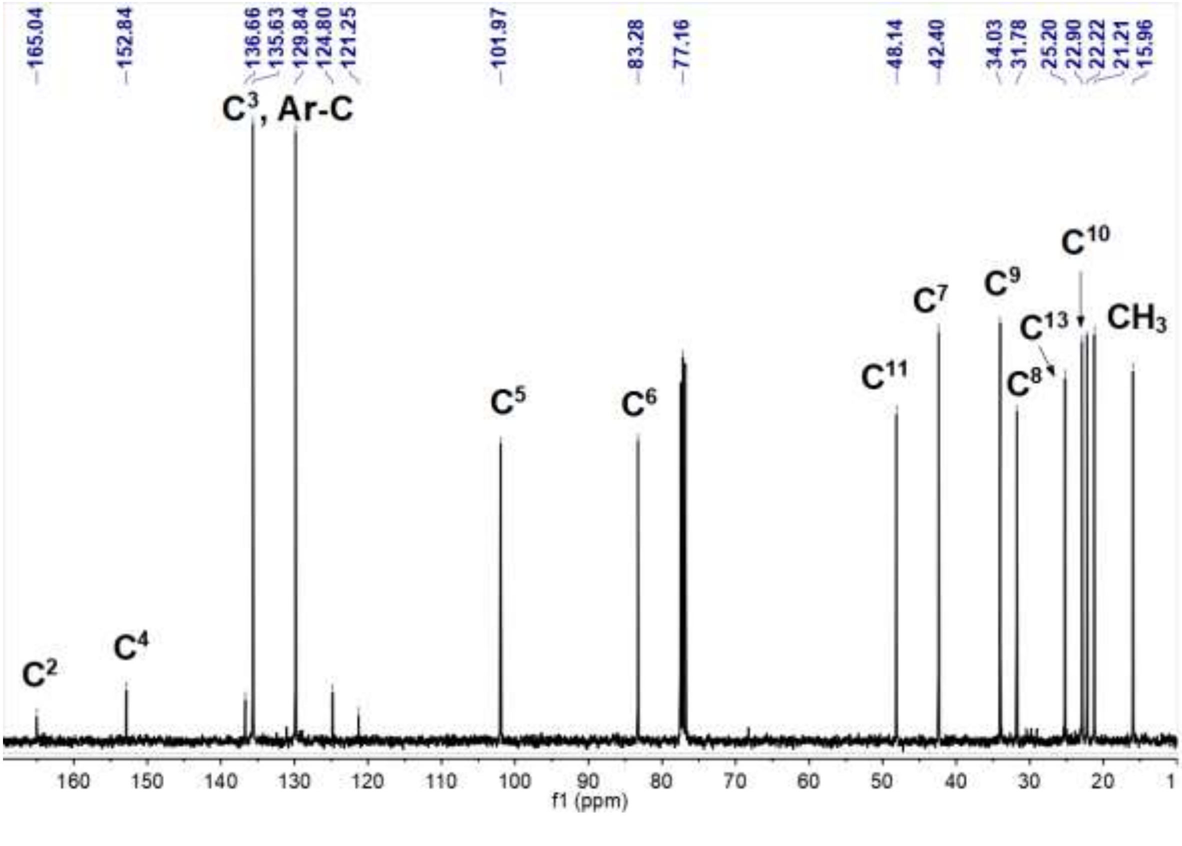

Figure S1. ^{1}H (a) and $^{13}C\{^{1}H\}$ (b) NMR spectra of compound **8** ($CDCl_3$).

a)

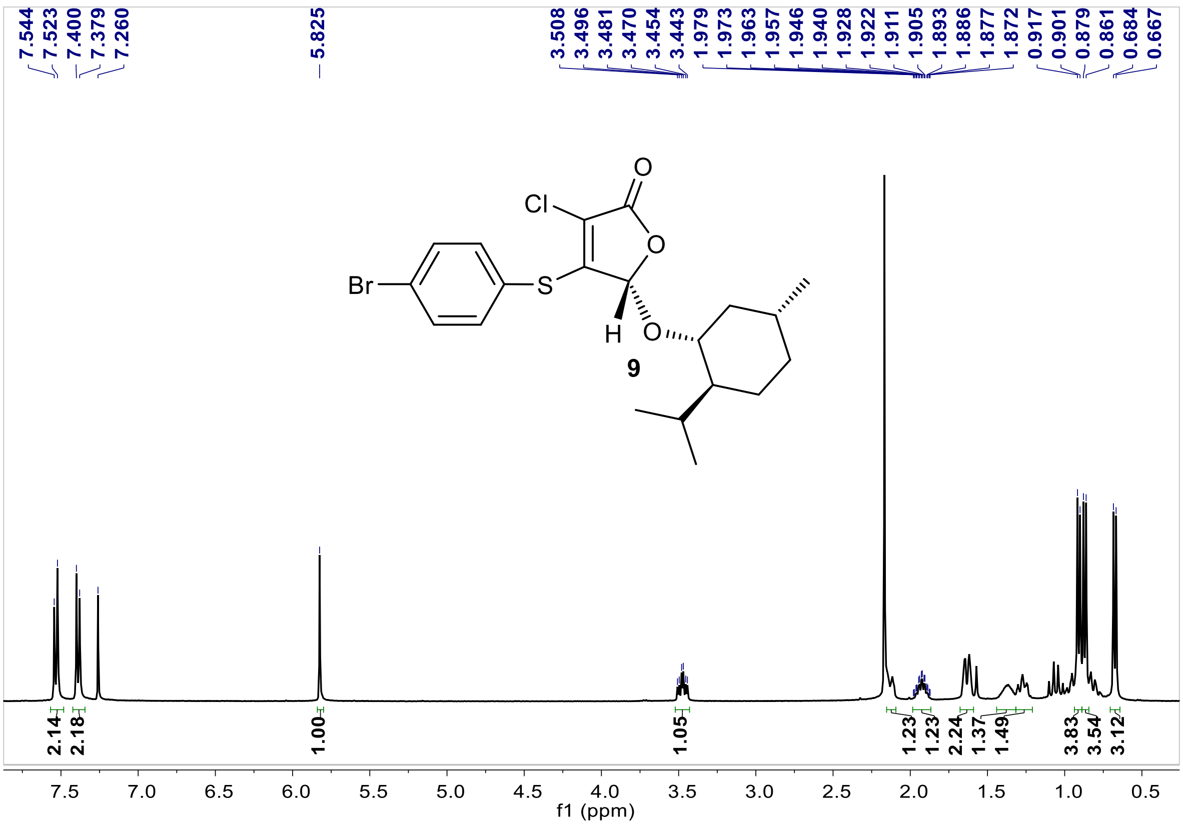

b)

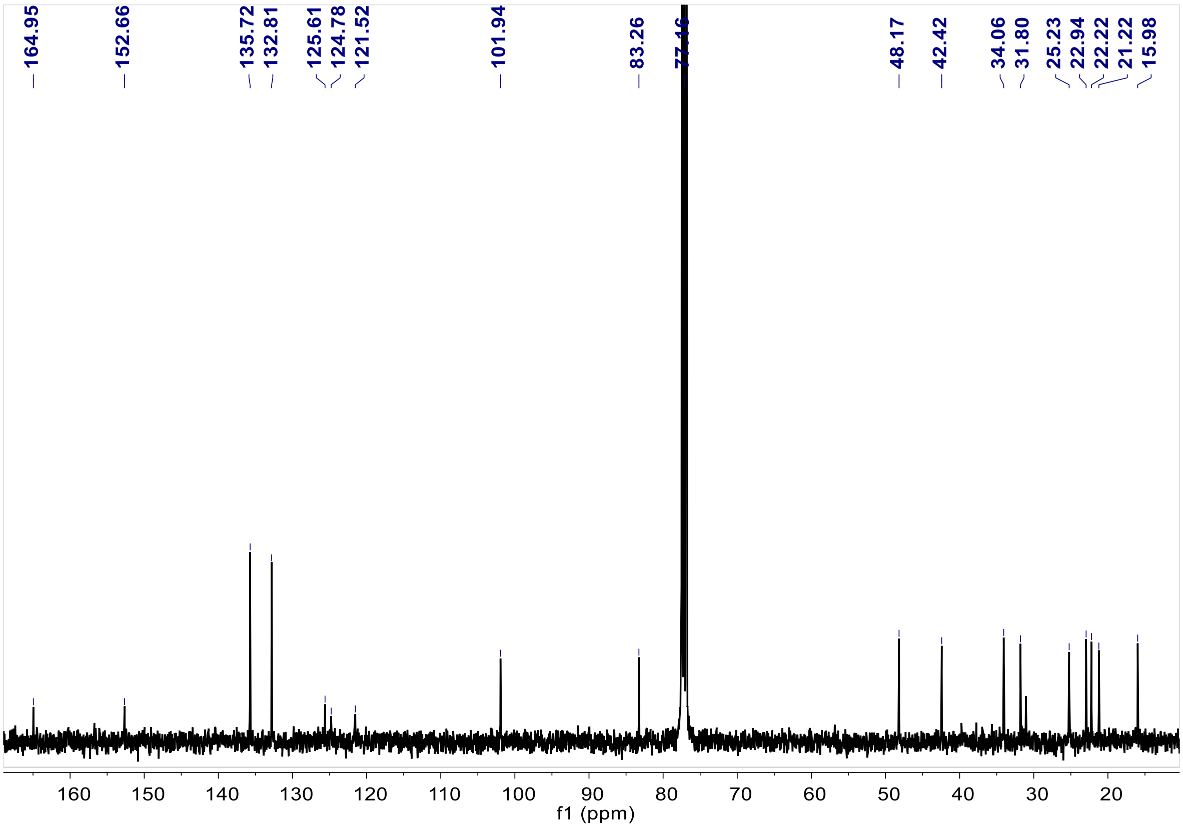

Figure S2. ^{1}H (a) and ^{13}C{^{1}H} (b) NMR spectra of compound **9** ($CDCl_3$).

a)

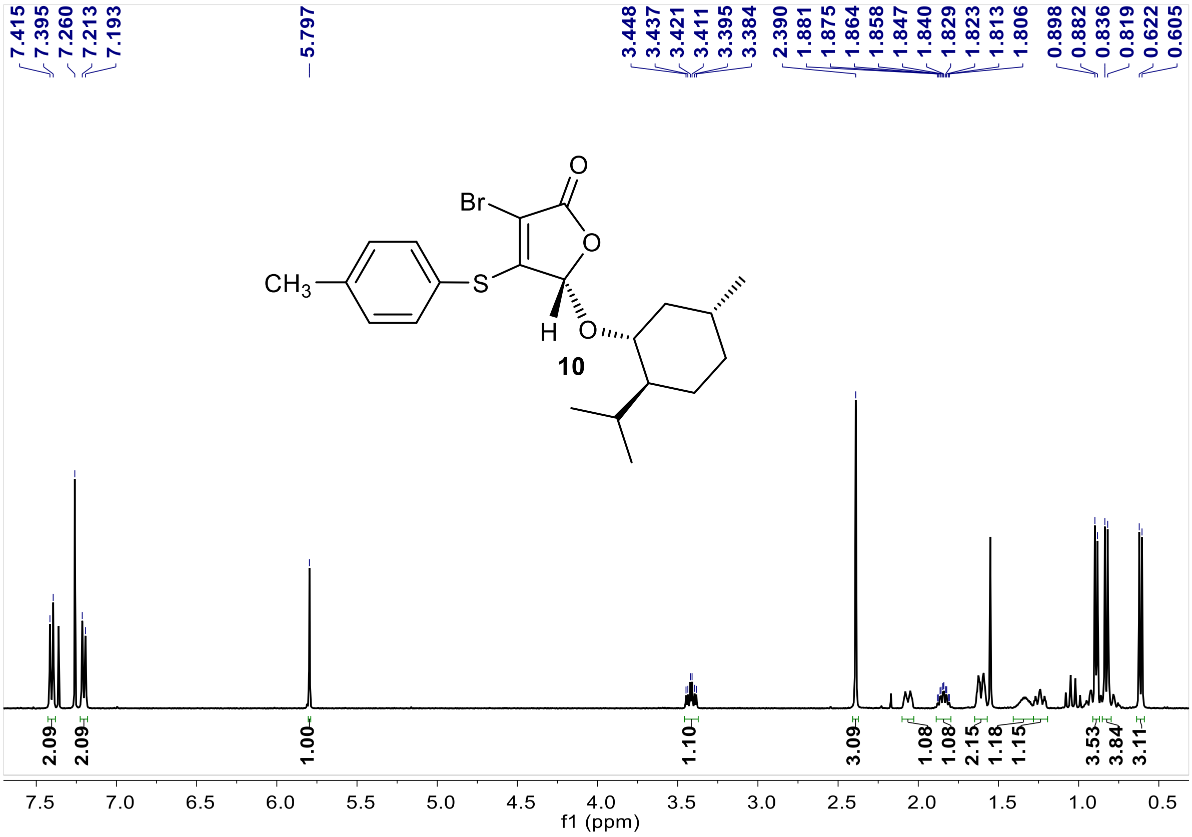

b)

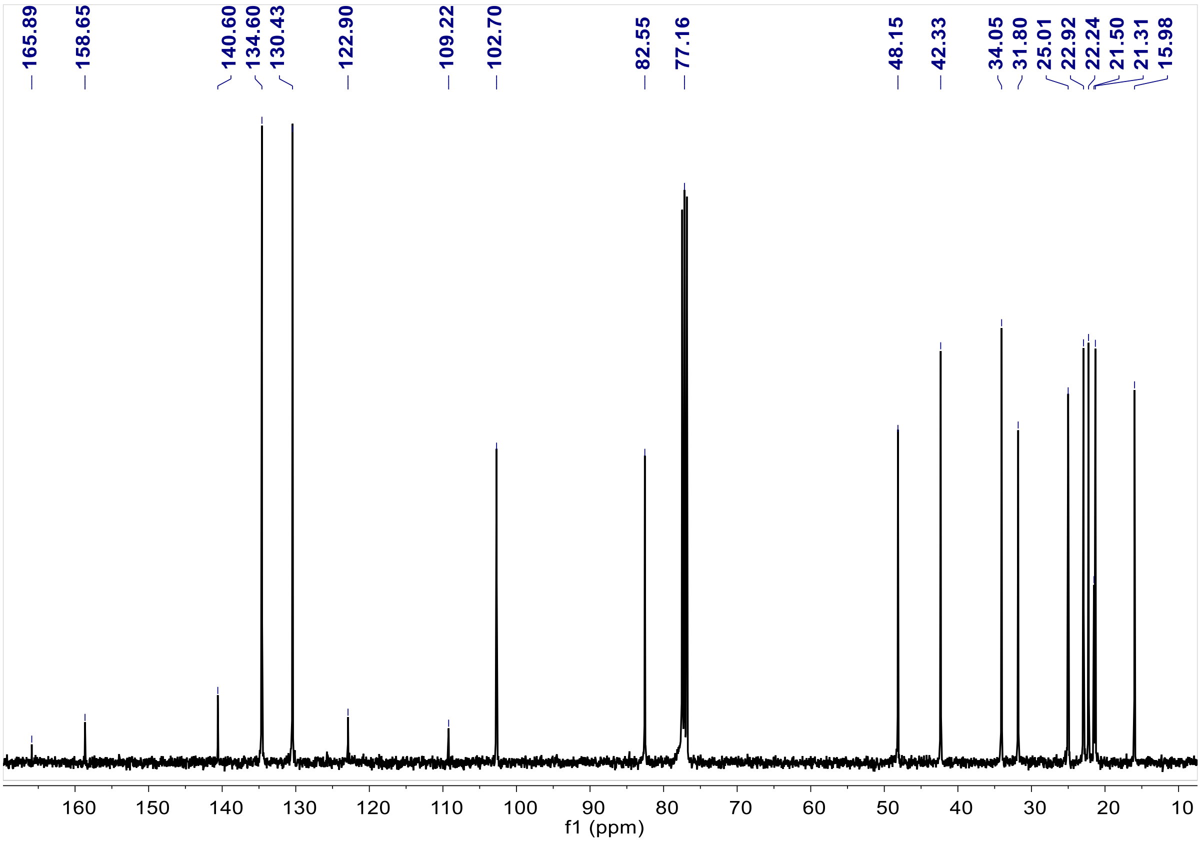

Figure S3. ^{1}H (a) and $^{13}C\{^{1}H\}$ (b) NMR spectra of compound **10** ($CDCl_3$).

a)

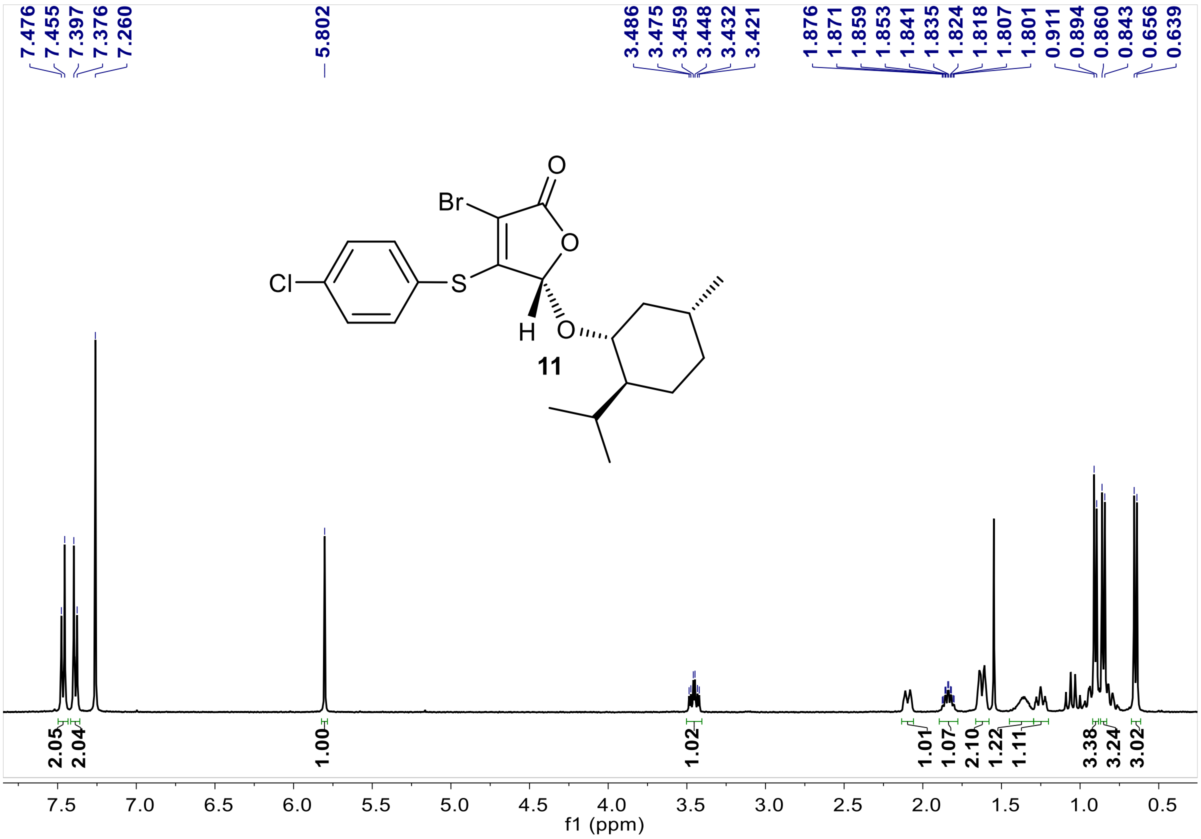

b)

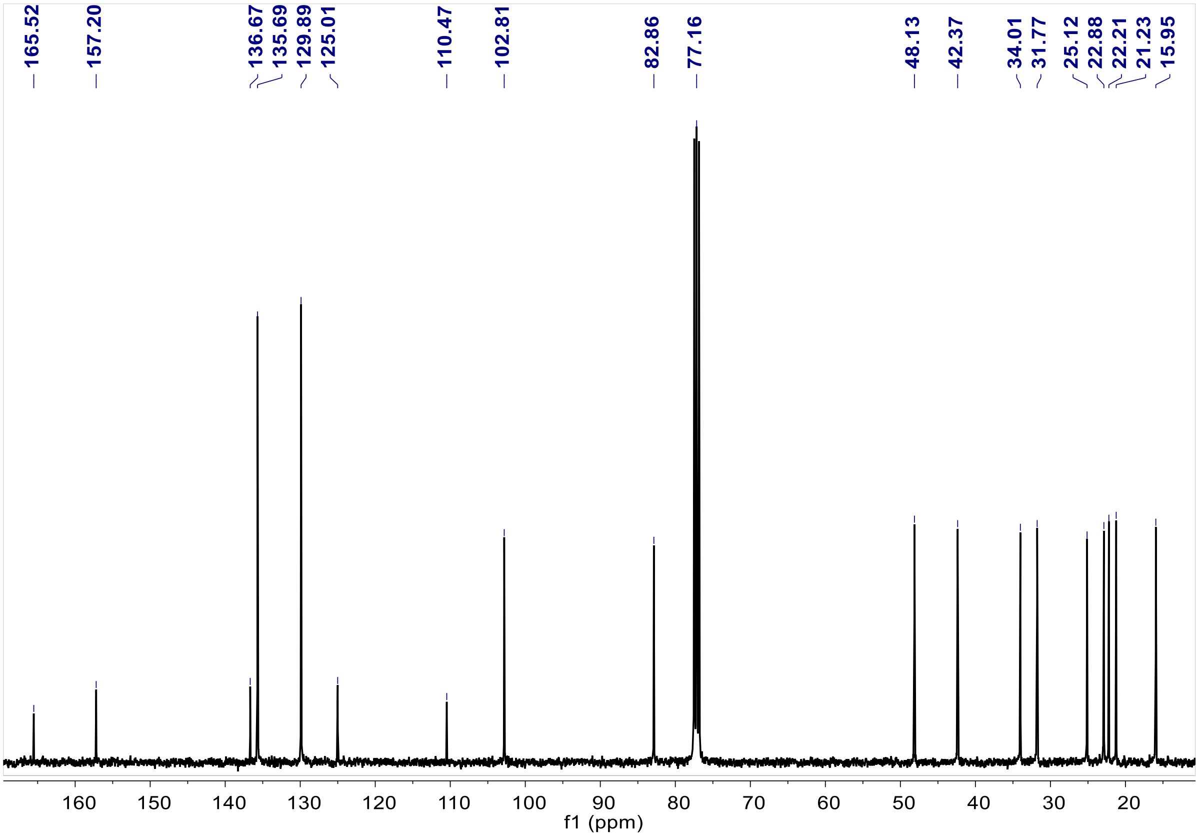

Figure S4. ^{1}H (a) and ^{13}C{^{1}H} (b) NMR spectra of compound **11** ($CDCl_3$).

a)

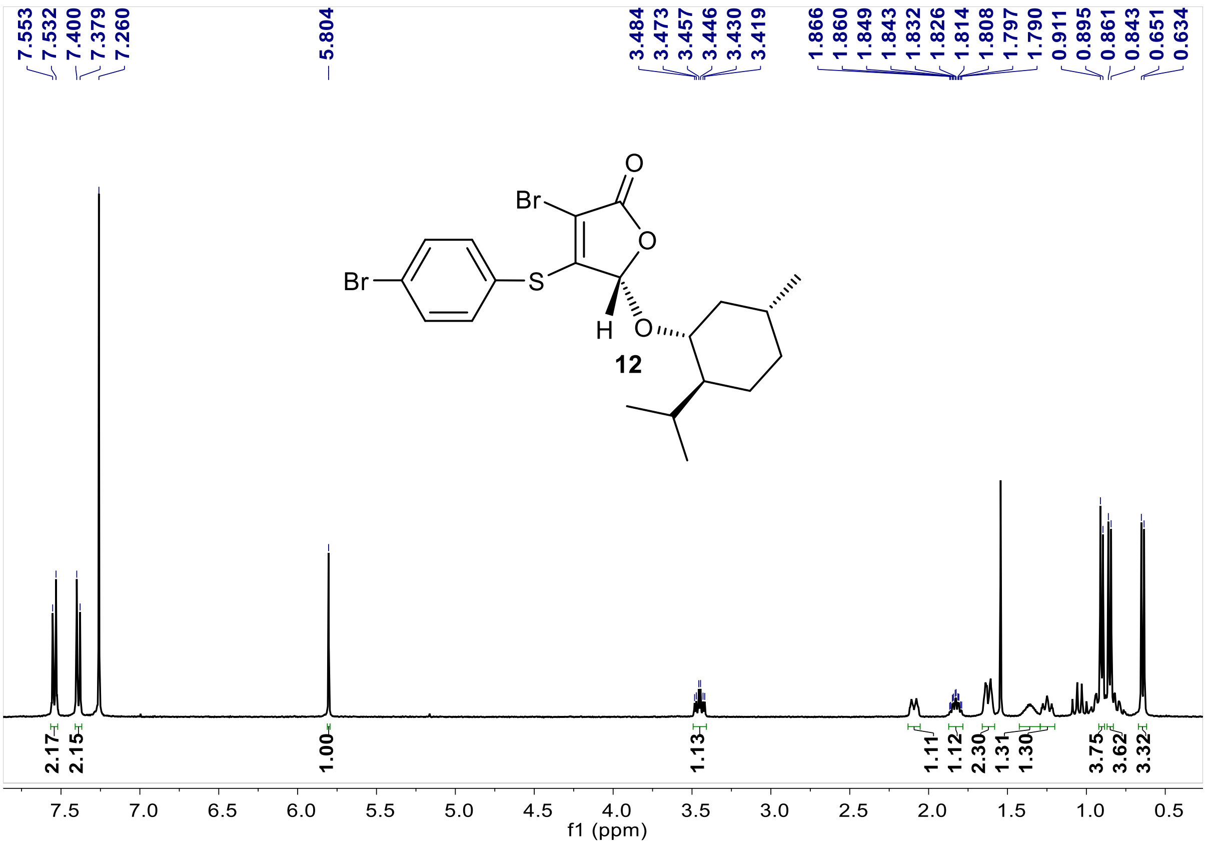

b)

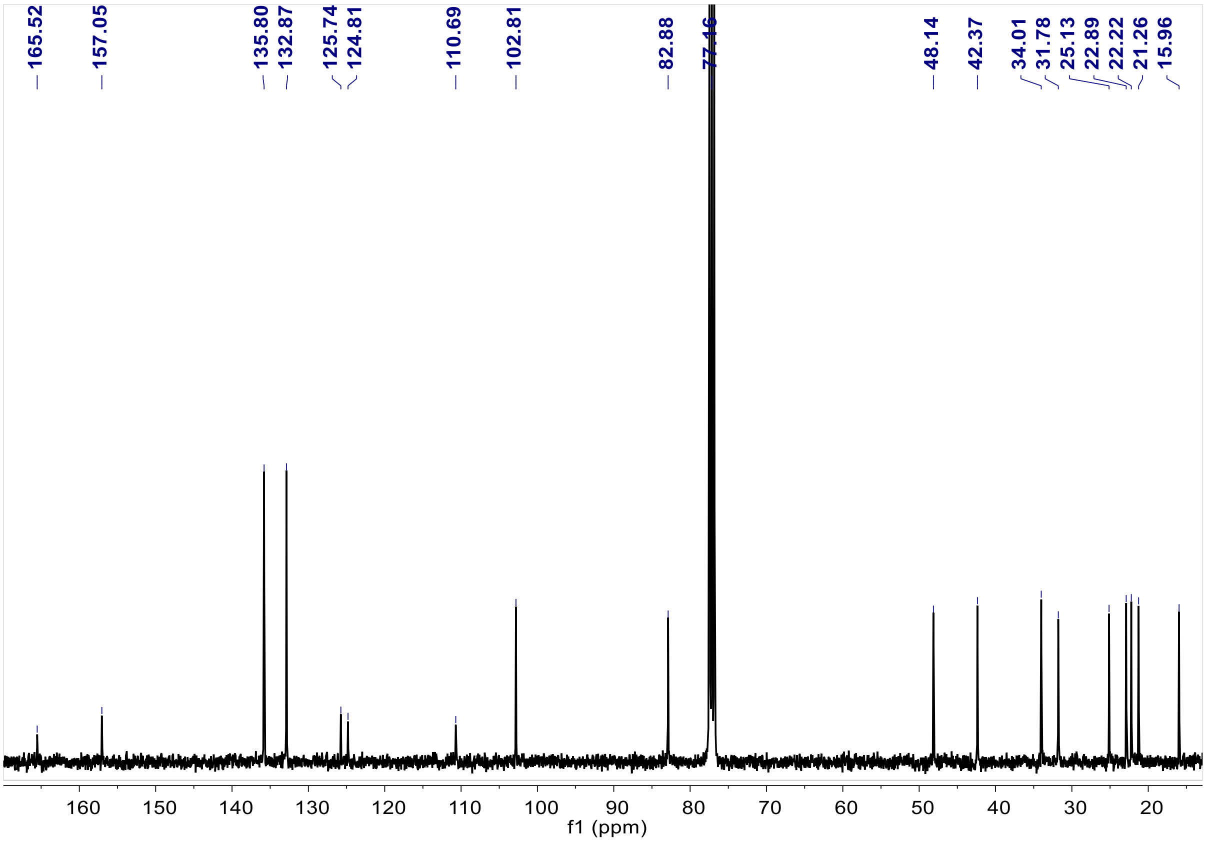

Figure S5. ^{1}H (a) and $^{13}C\{^{1}H\}$ (b) NMR spectra of compound **12** ($CDCl_3$).

a)

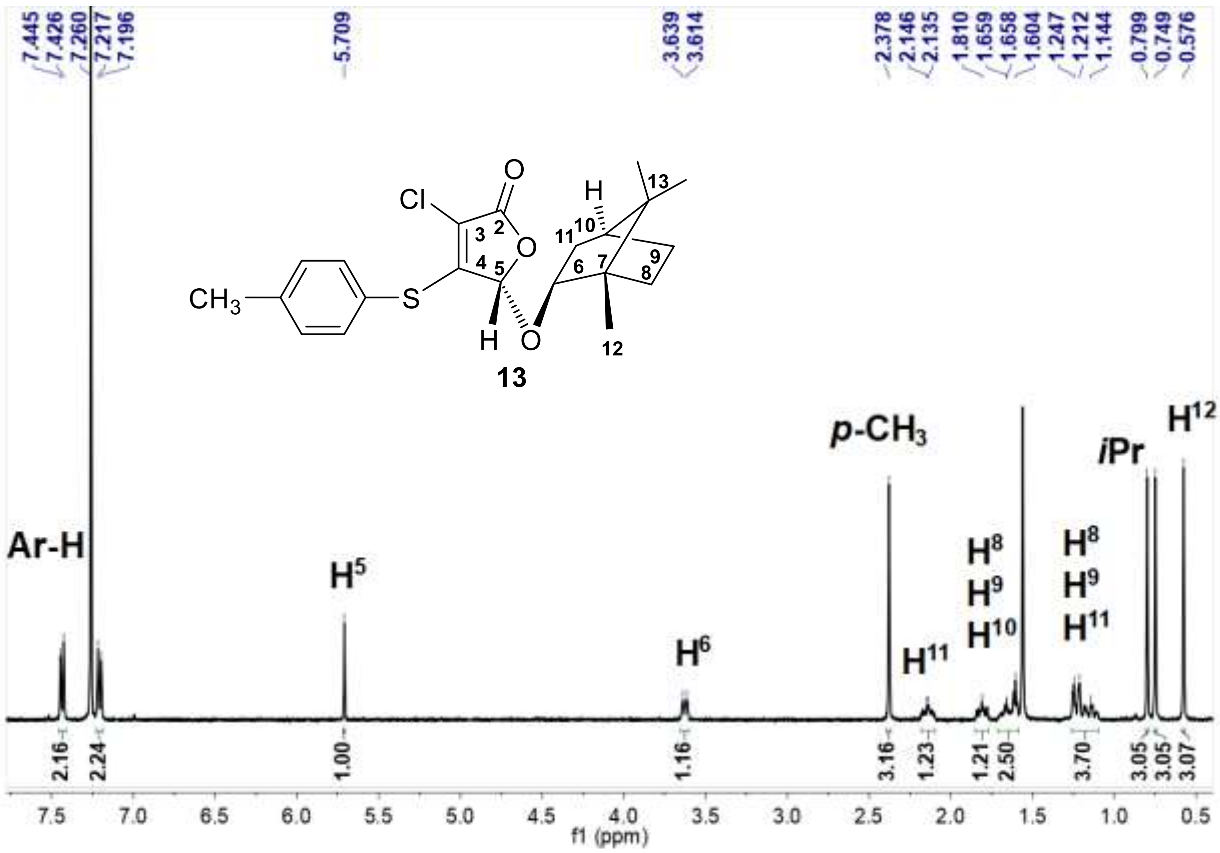

b)

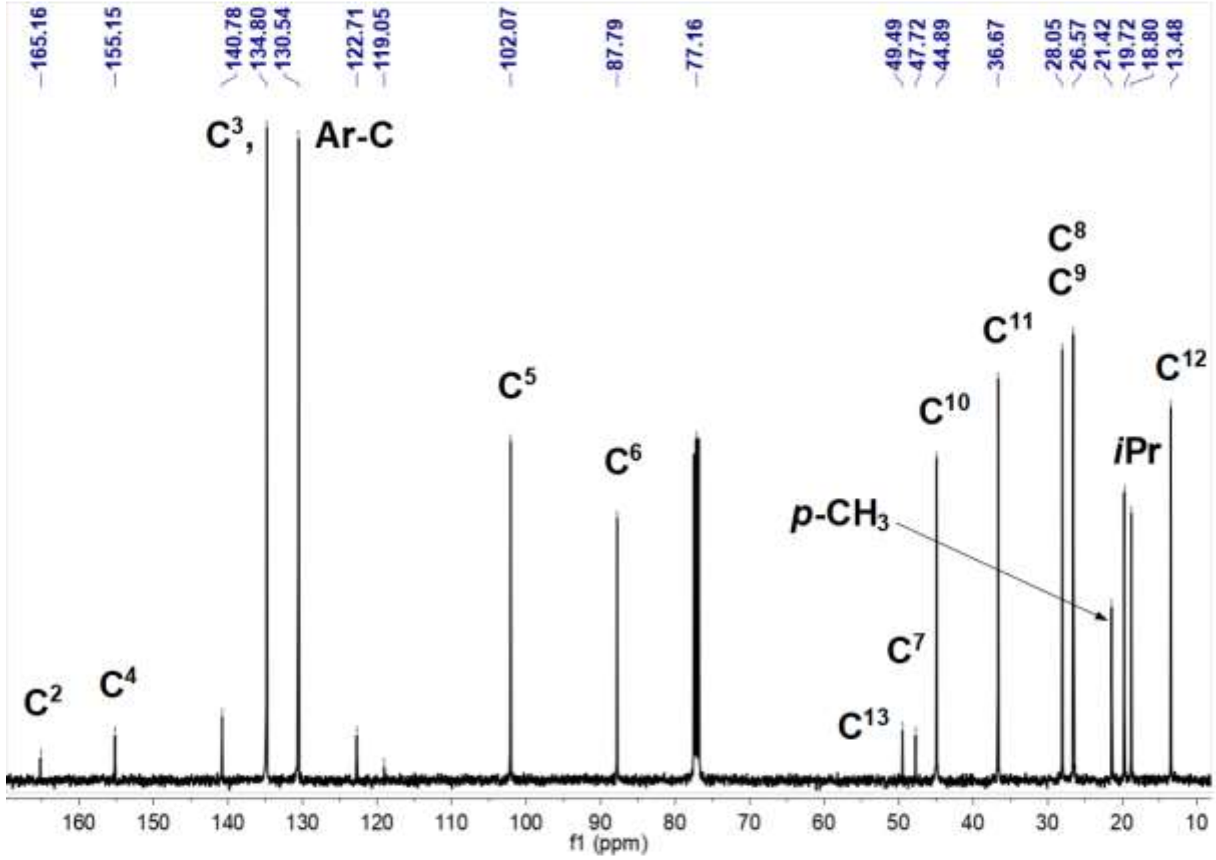

Figure S6. ^{1}H (a) and $^{13}C\{^{1}H\}$ (b) NMR spectra of compound **13** ($CDCl_3$).

a)

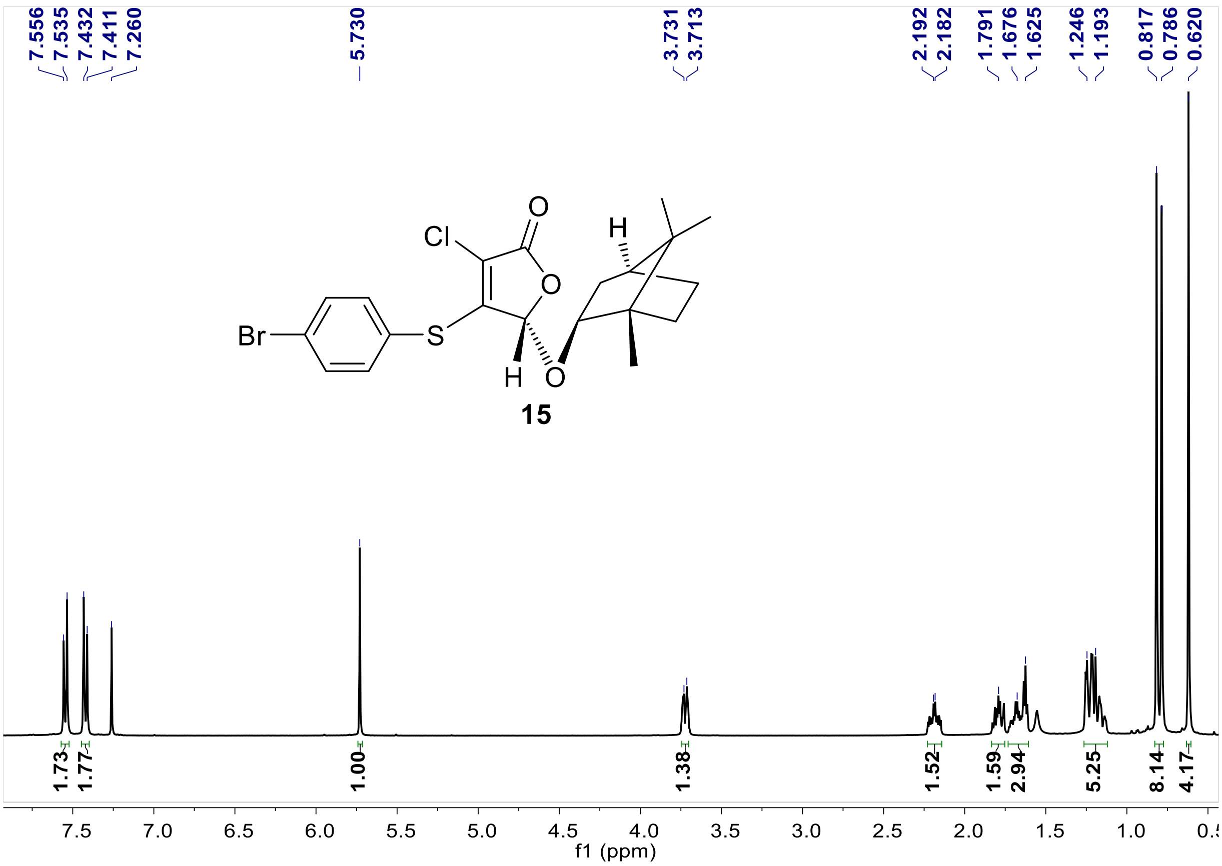

b)

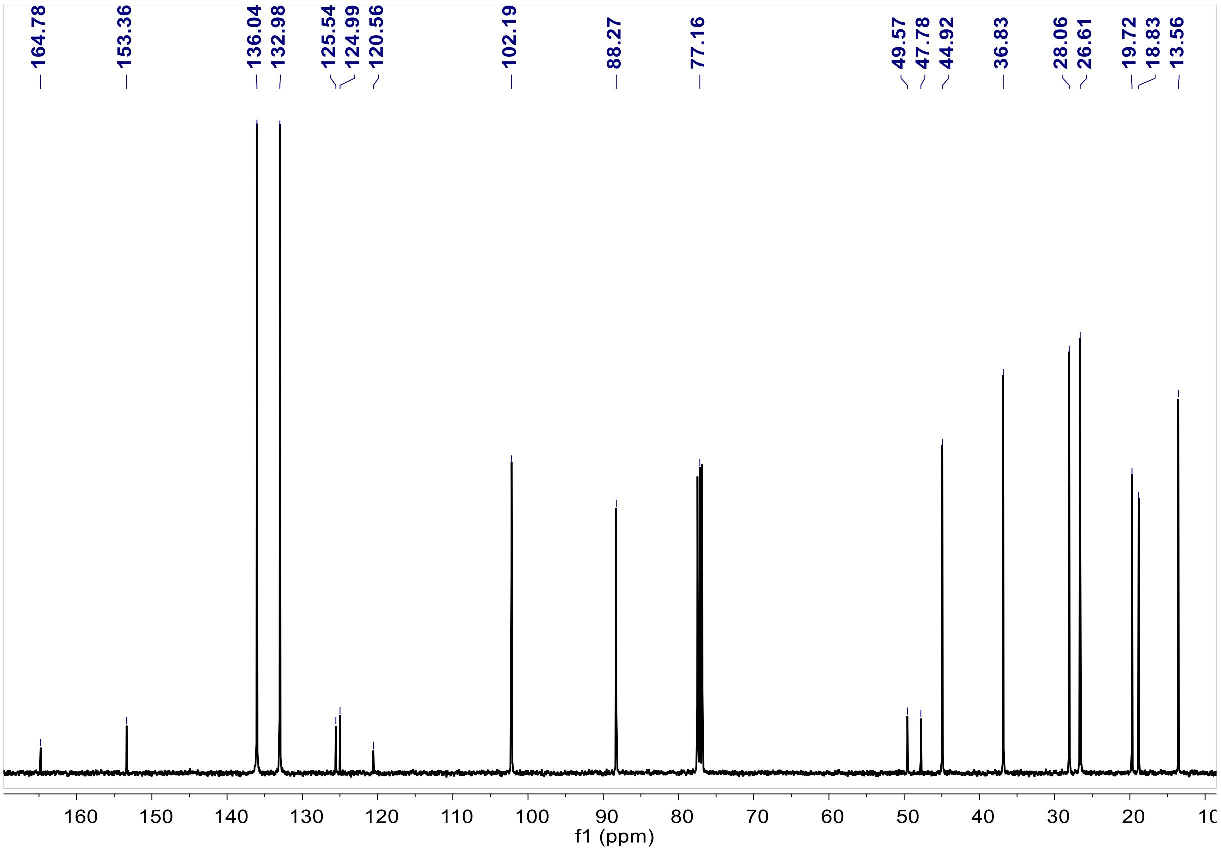

Figure S7. ^{1}H (a) and ^{13}C{^{1}H} (b) NMR spectra of compound **15** ($CDCl_3$).

a)

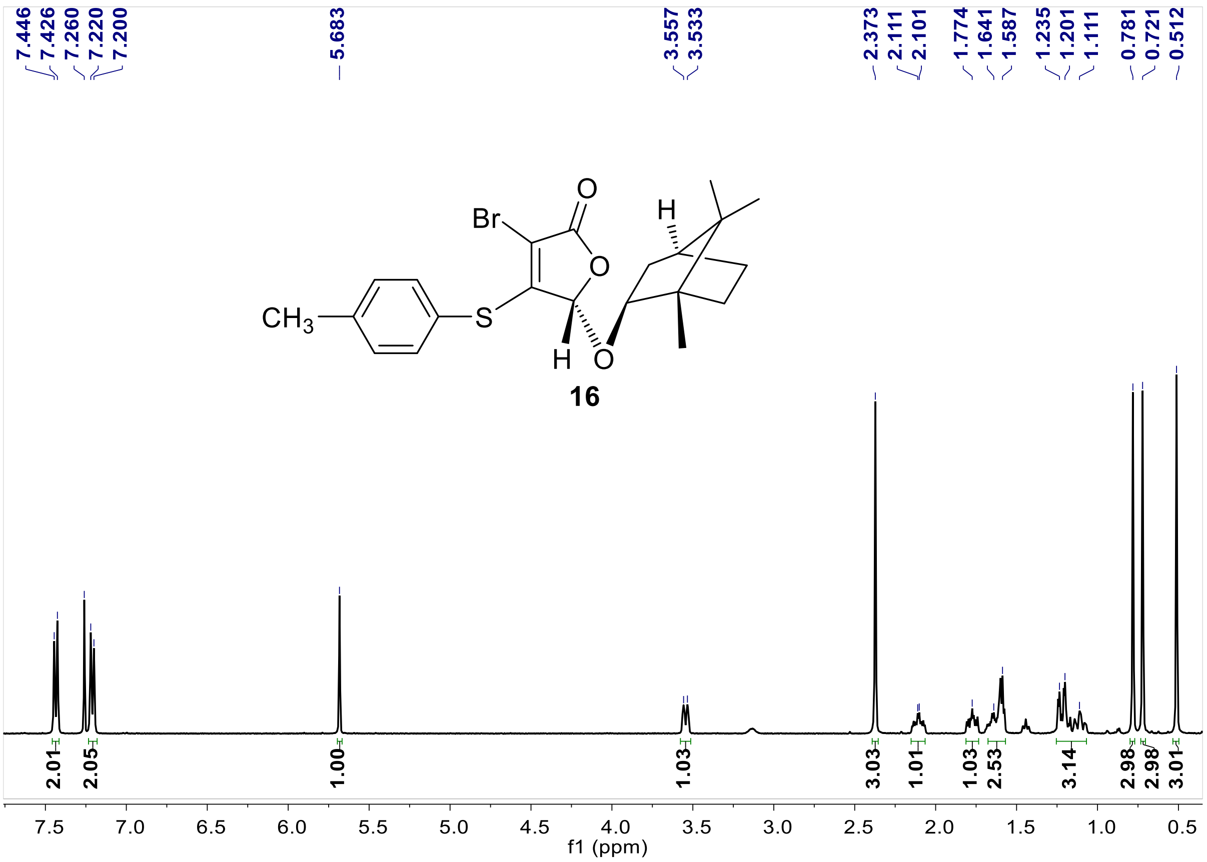

b)

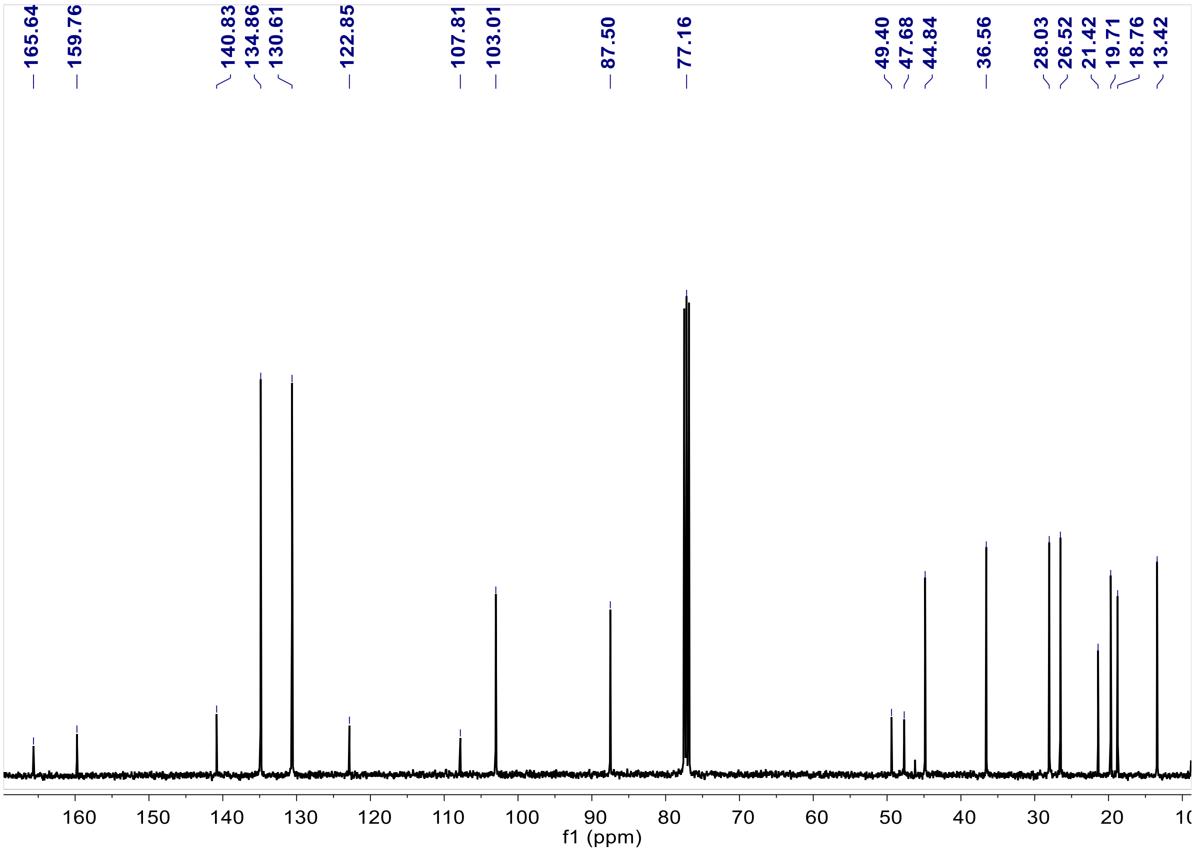

Figure S8. ^{1}H (a) and $^{13}C\{^{1}H\}$ (b) NMR spectra of compound **16** ($CDCl_3$).

a)

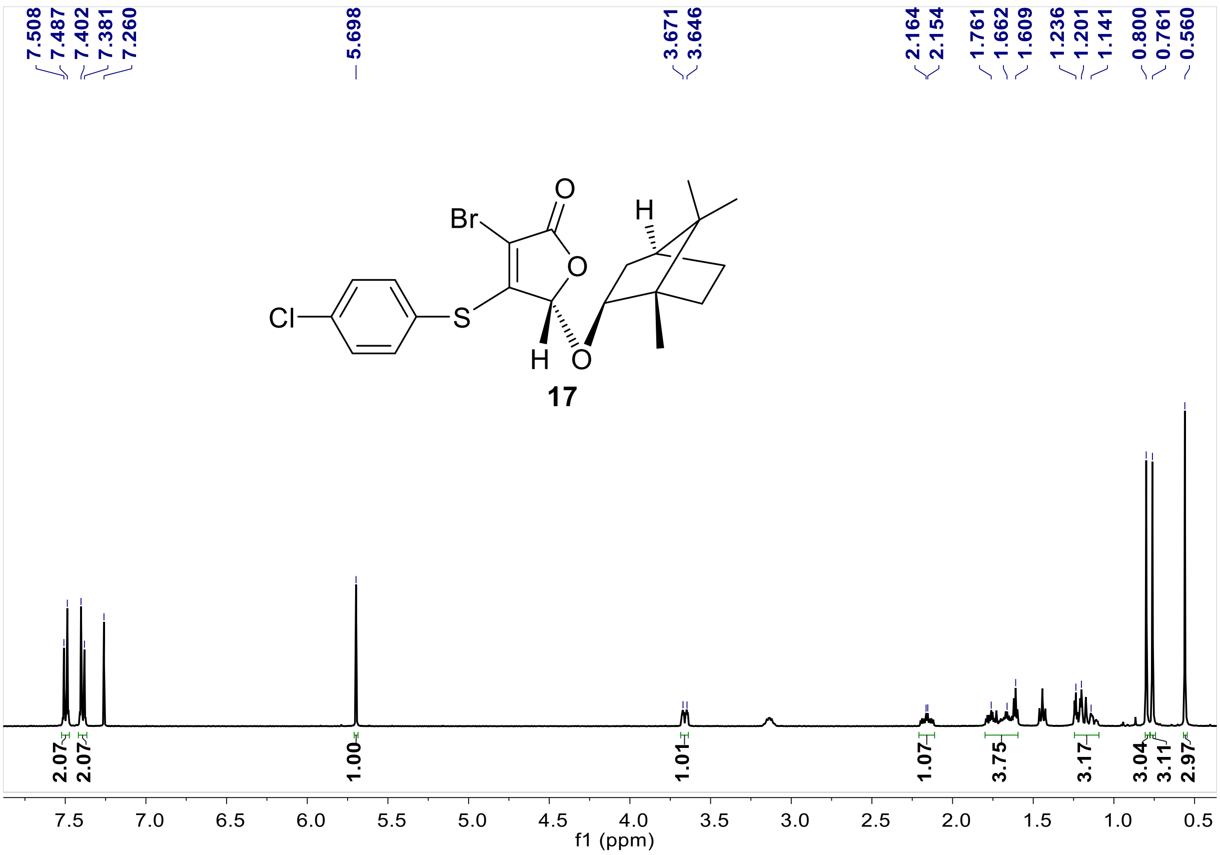

b)

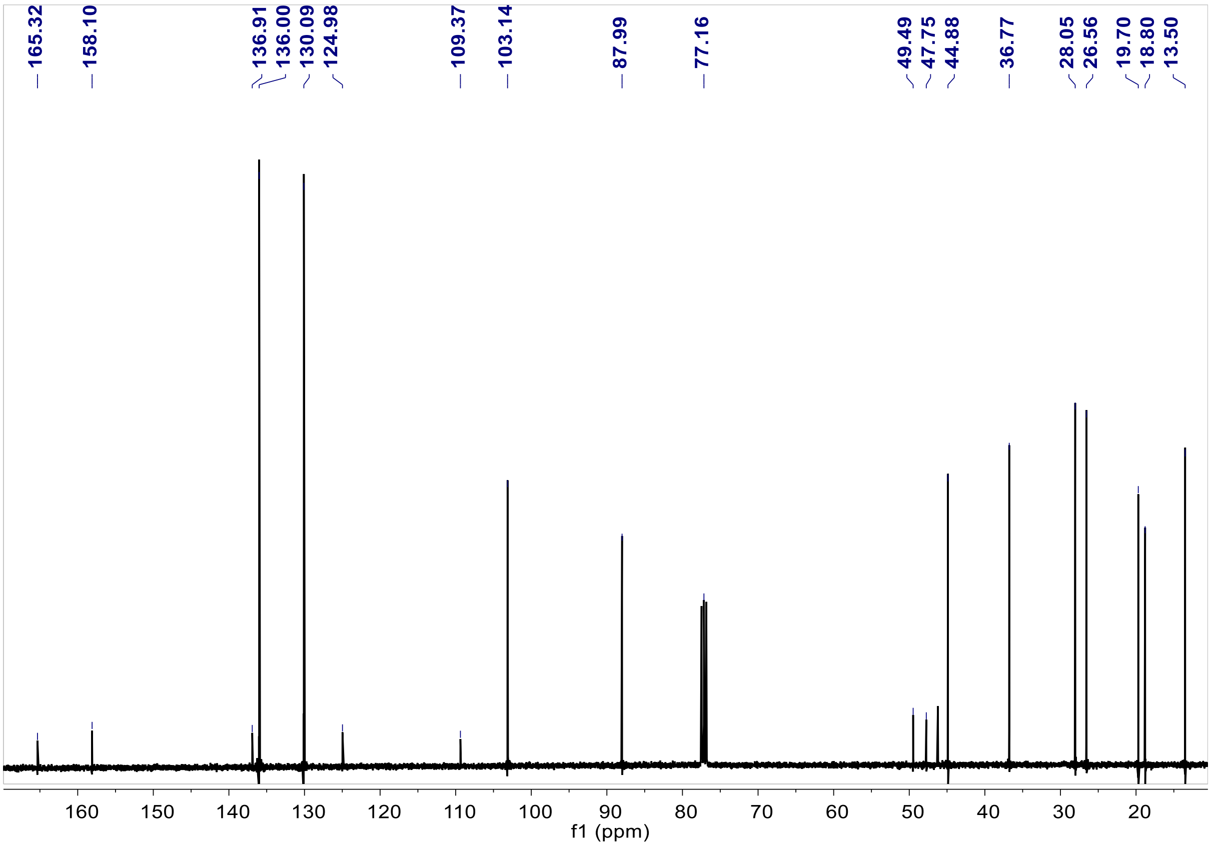

Figure S9. ^{1}H (a) and ^{13}C{^{1}H} (b) NMR spectra of compound **17** ($CDCl_3$).

a)

b)

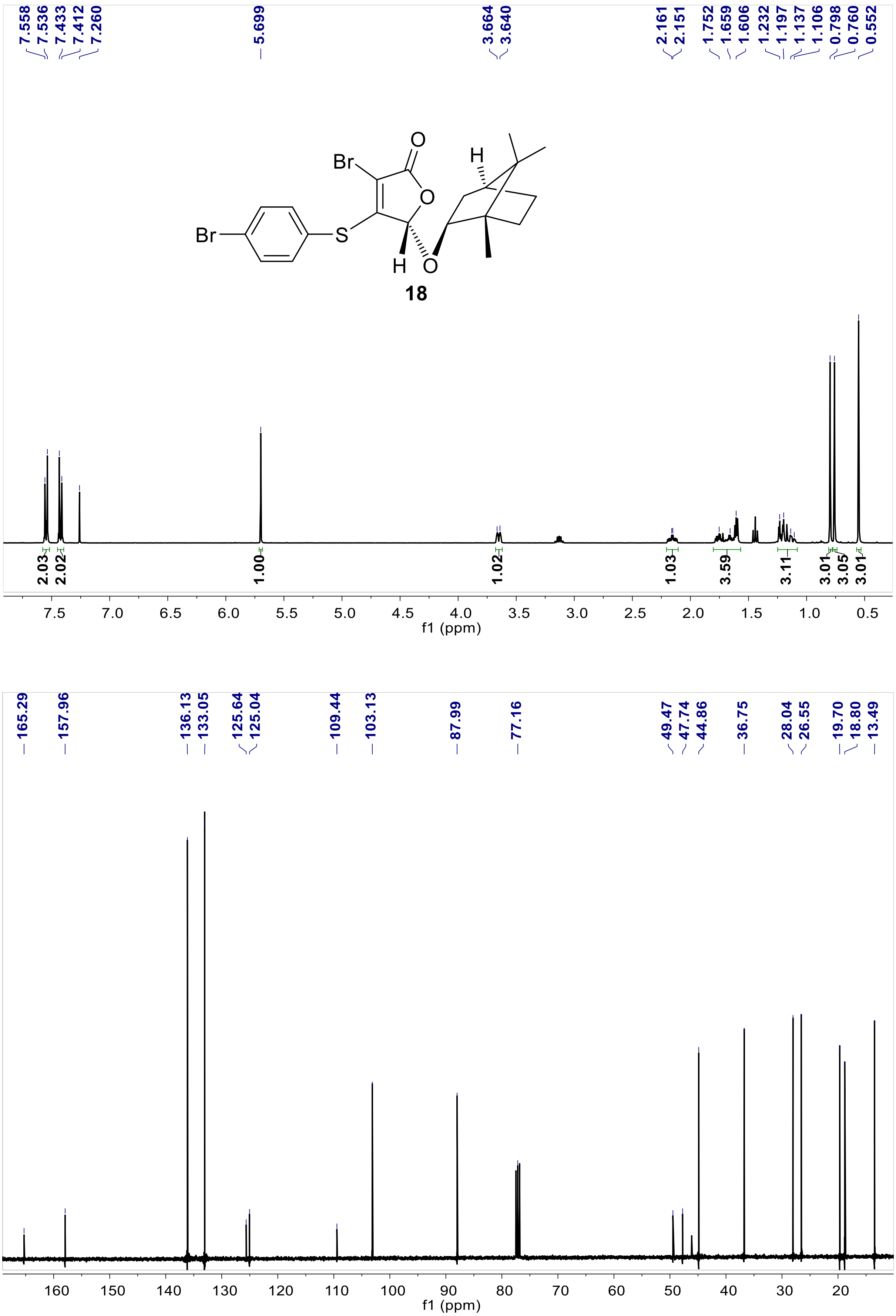

Figure S10. ^{1}H (a) and $^{13}C\{^{1}H\}$ (b) NMR spectra of compound **18** ($CDCl_3$).

^{1}H and $^{13}C\{^{1}H\}$ NMR spectra of sulfones

a)

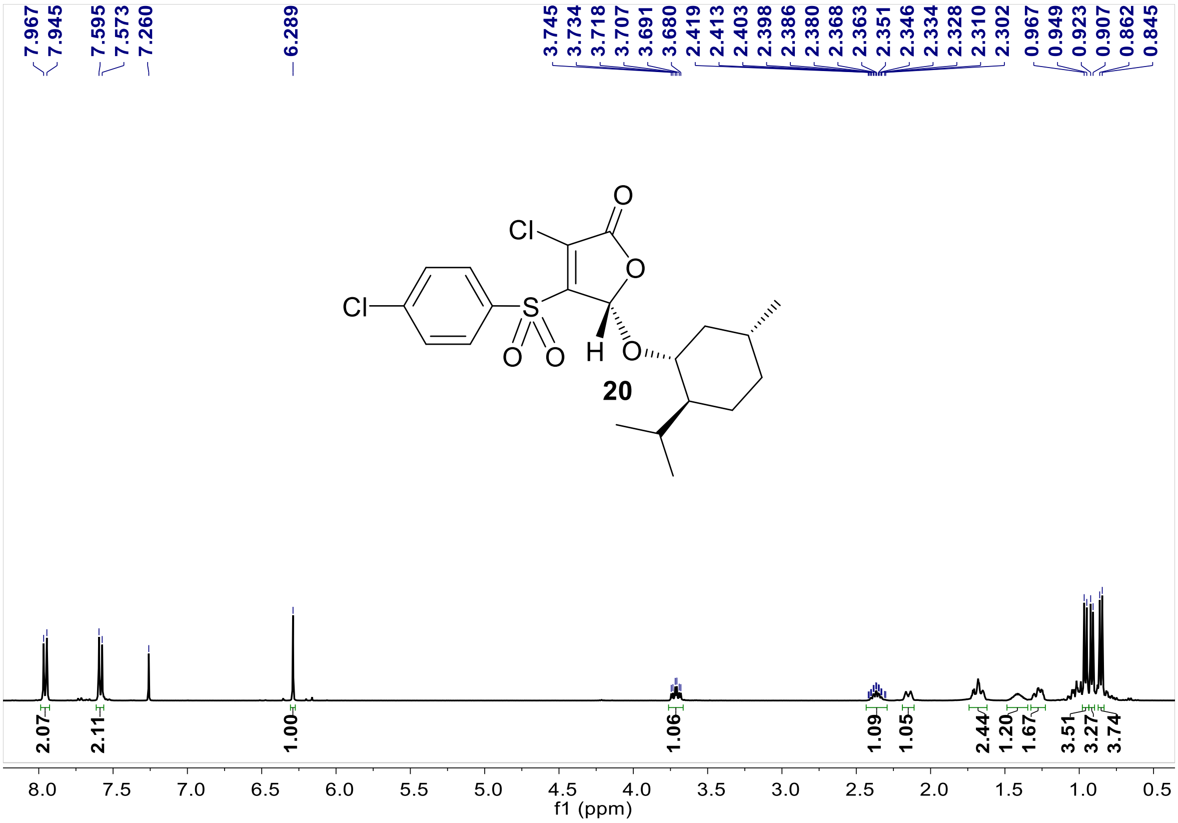

b)

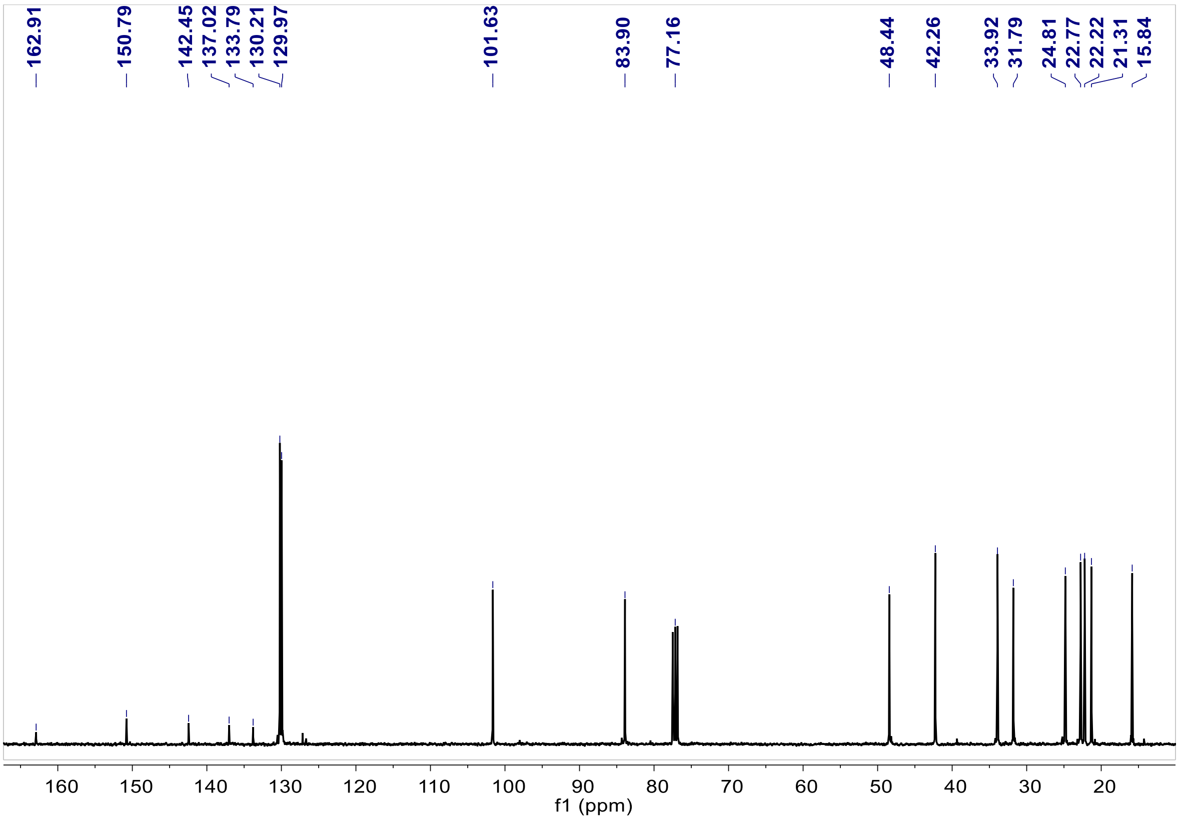

Figure S11. ^{1}H (a) and $^{13}C\{^{1}H\}$ (b) NMR spectra of compound **20** ($CDCl_3$).

a)

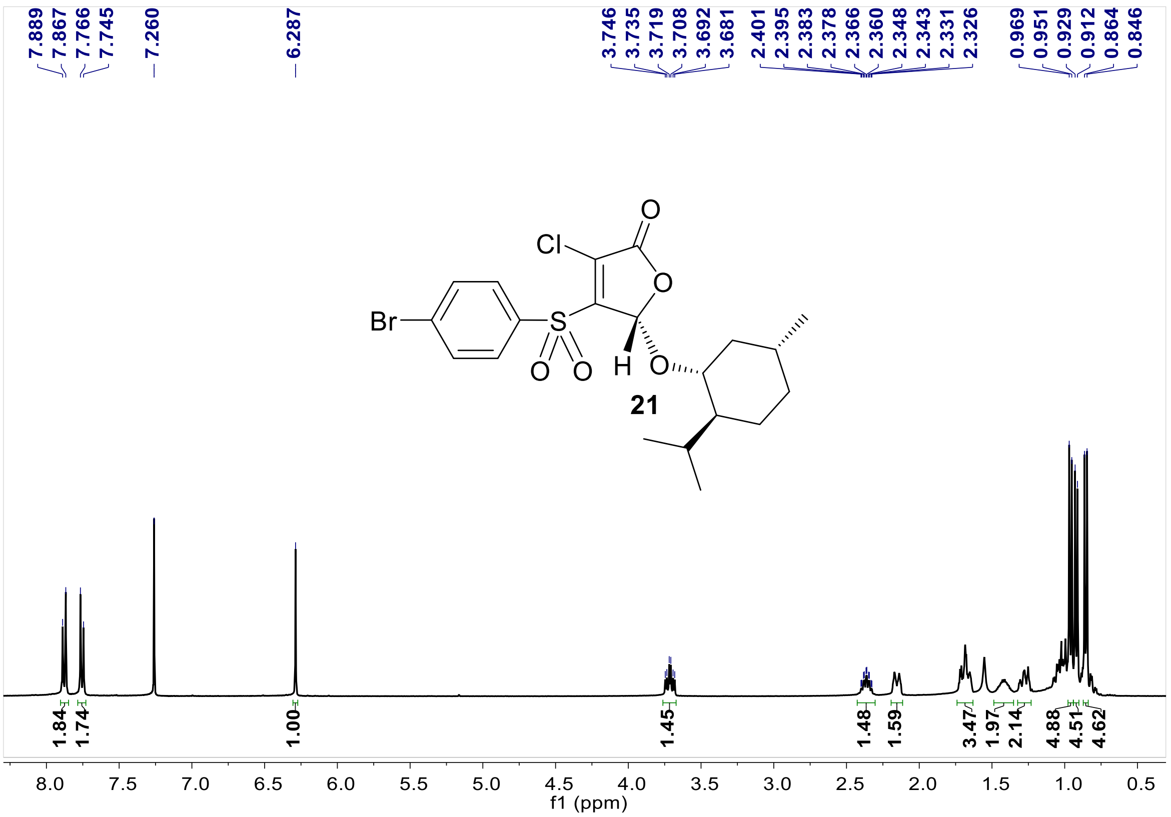

b)

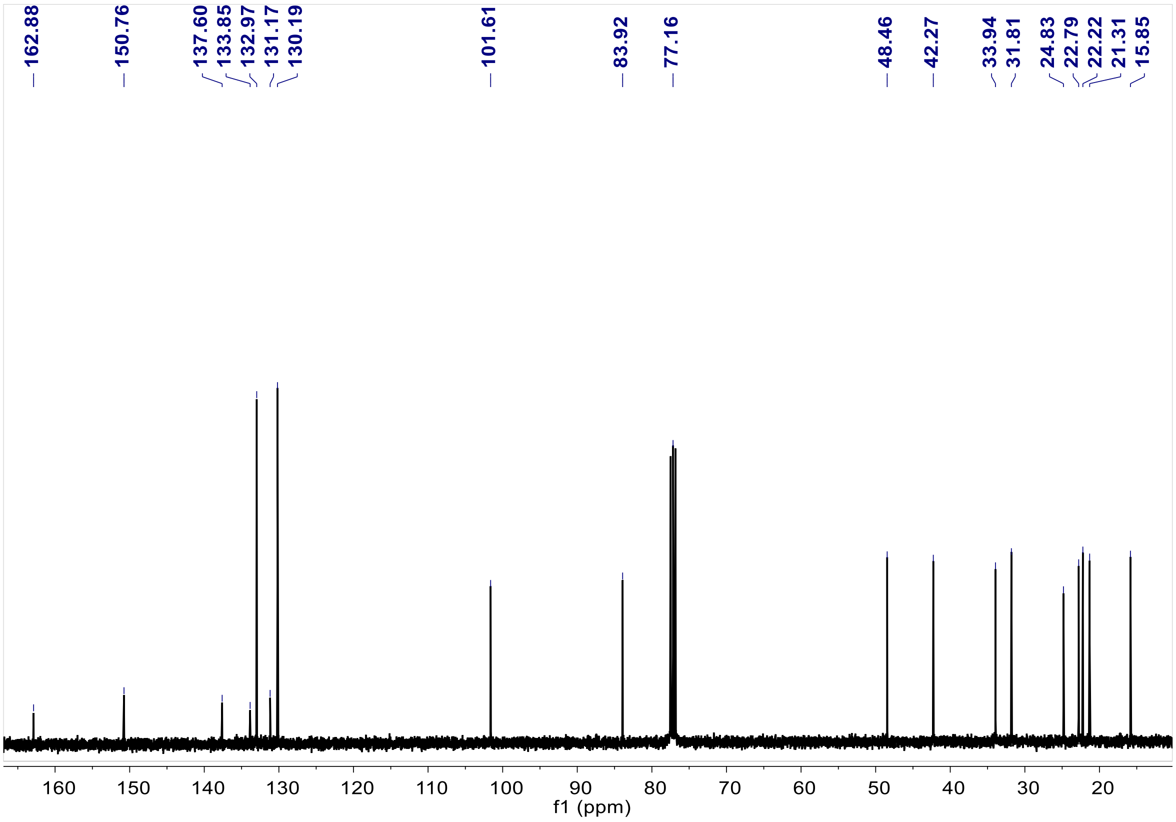

Figure S12. ^{1}H (a) and $^{13}C\{^{1}H\}$ (b) NMR spectra of compound **21** ($CDCl_3$).

a)

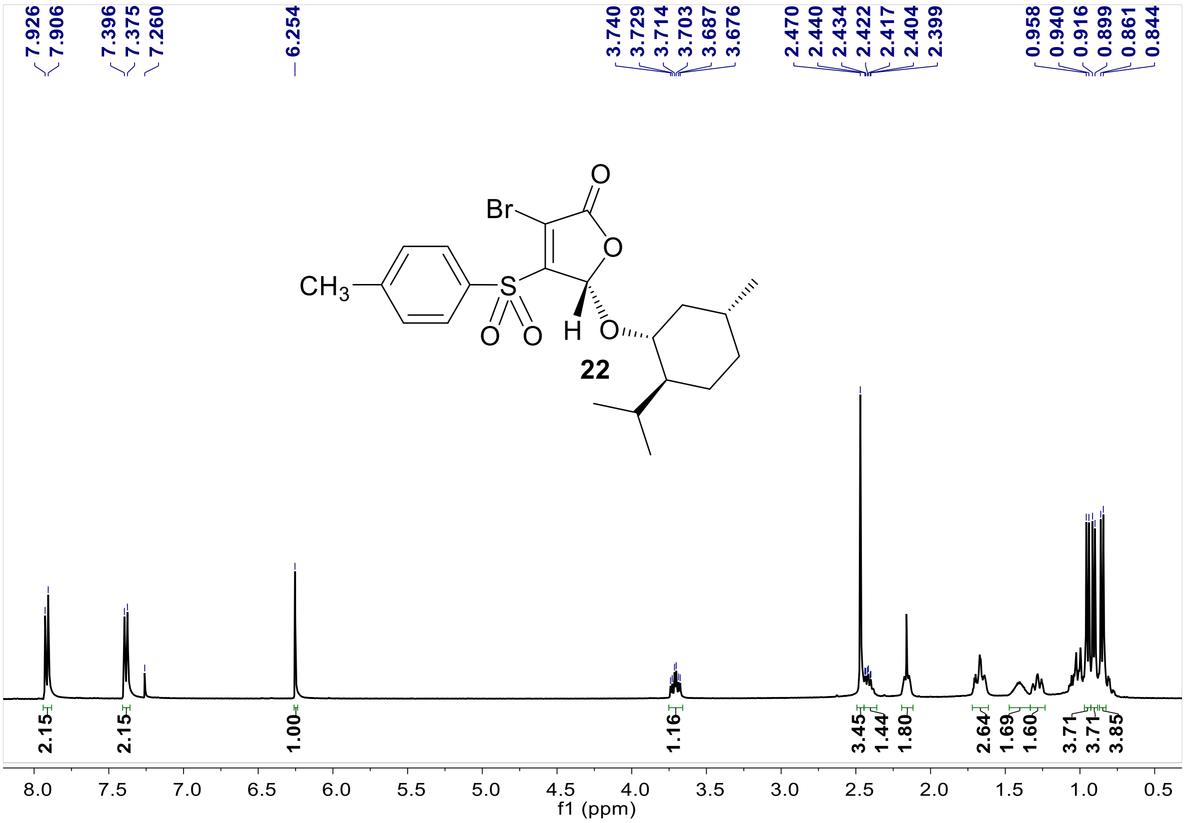

b)

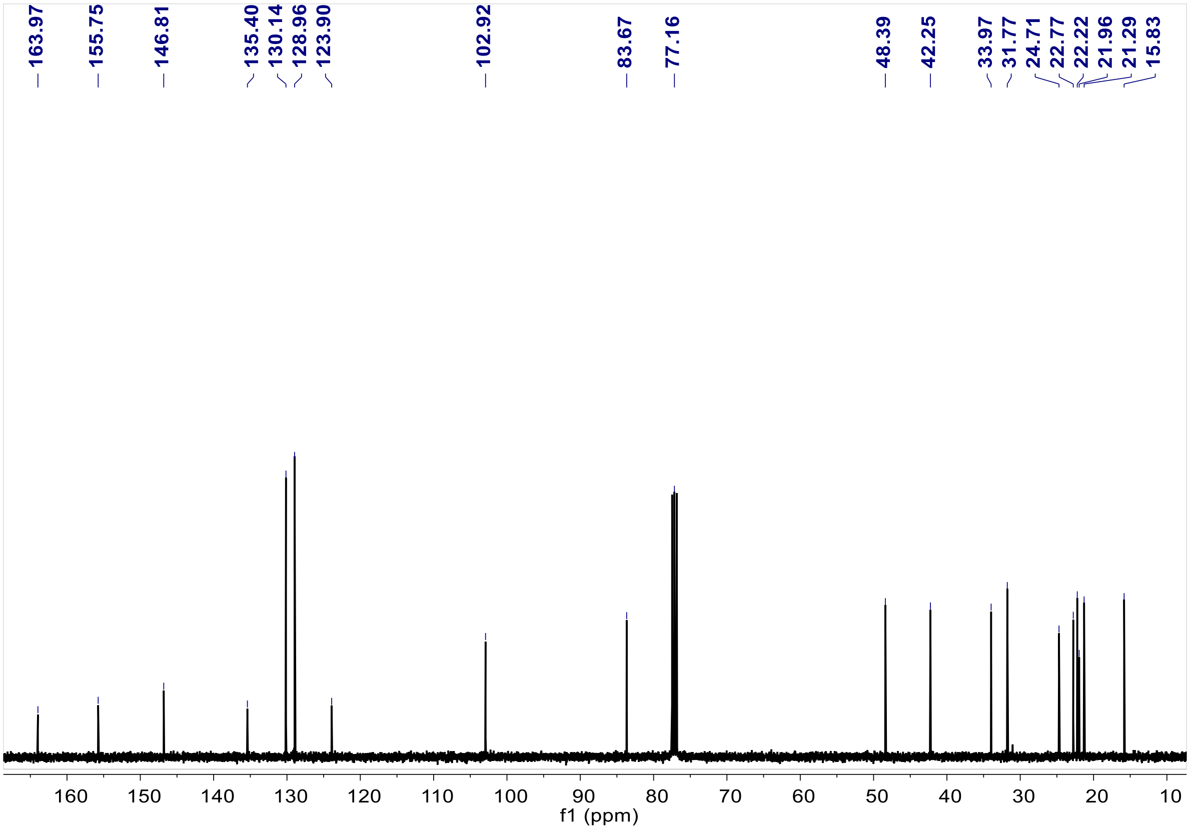

Figure S13. ^{1}H (a) and $^{13}C\{^{1}H\}$ (b) NMR spectra of compound **22** ($CDCl_3$).

a)

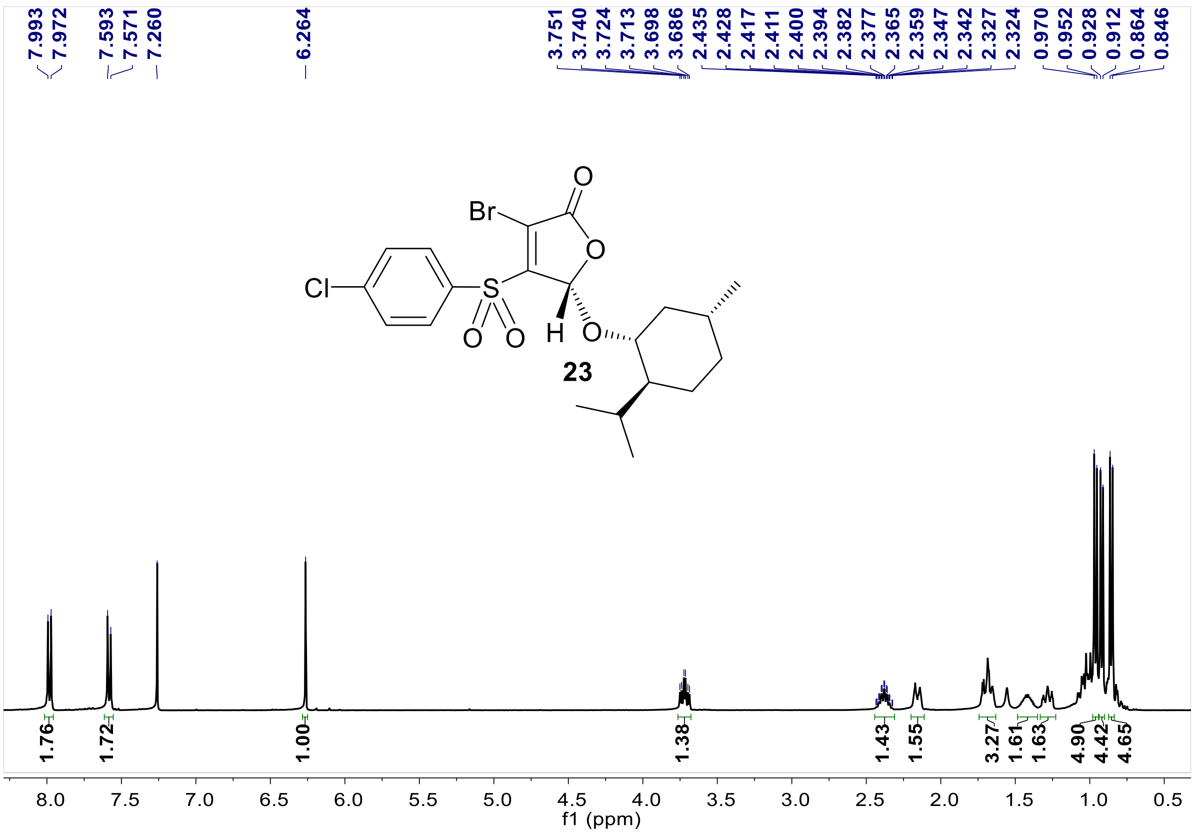

b)

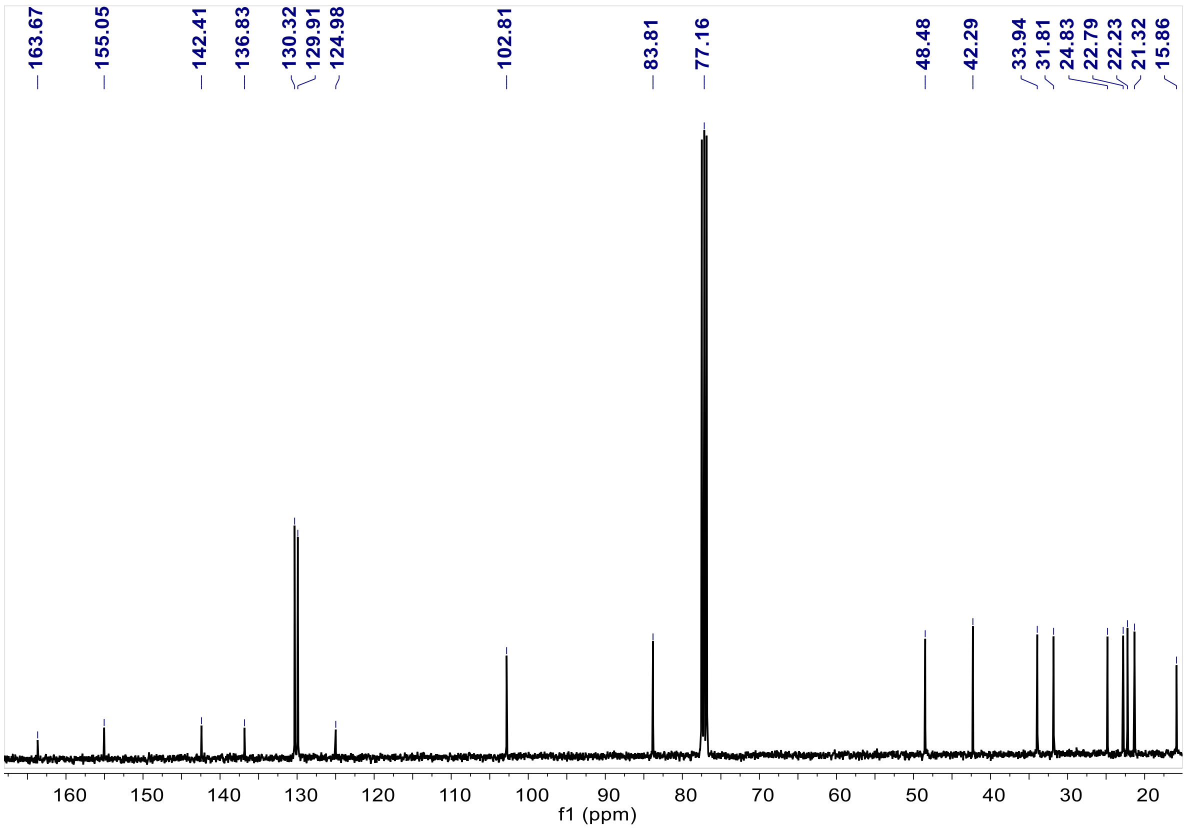

Figure S14. ^{1}H (a) and $^{13}C\{^{1}H\}$ (b) NMR spectra of compound **23** ($CDCl_3$).

a)

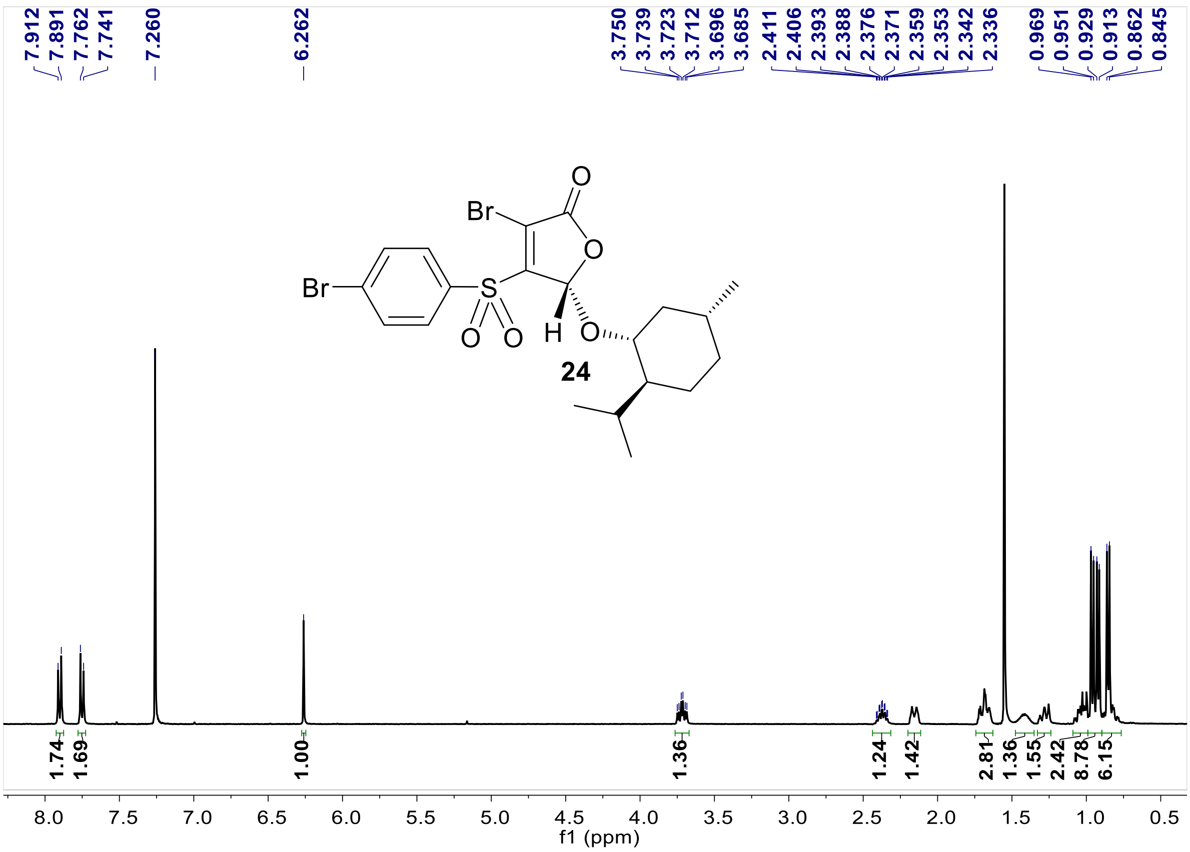

b)

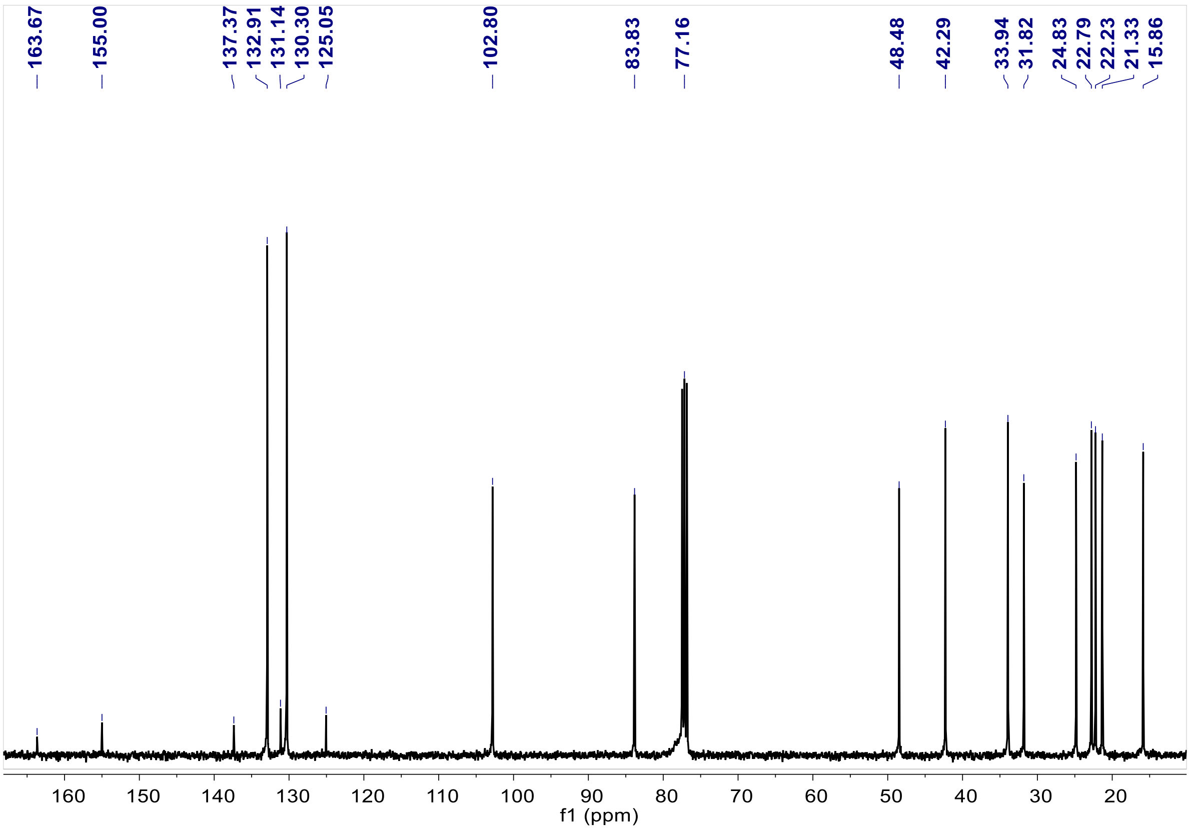

Figure S15. ^{1}H (a) and $^{13}C\{^{1}H\}$ (b) NMR spectra of compound **24** ($CDCl_3$).

a)

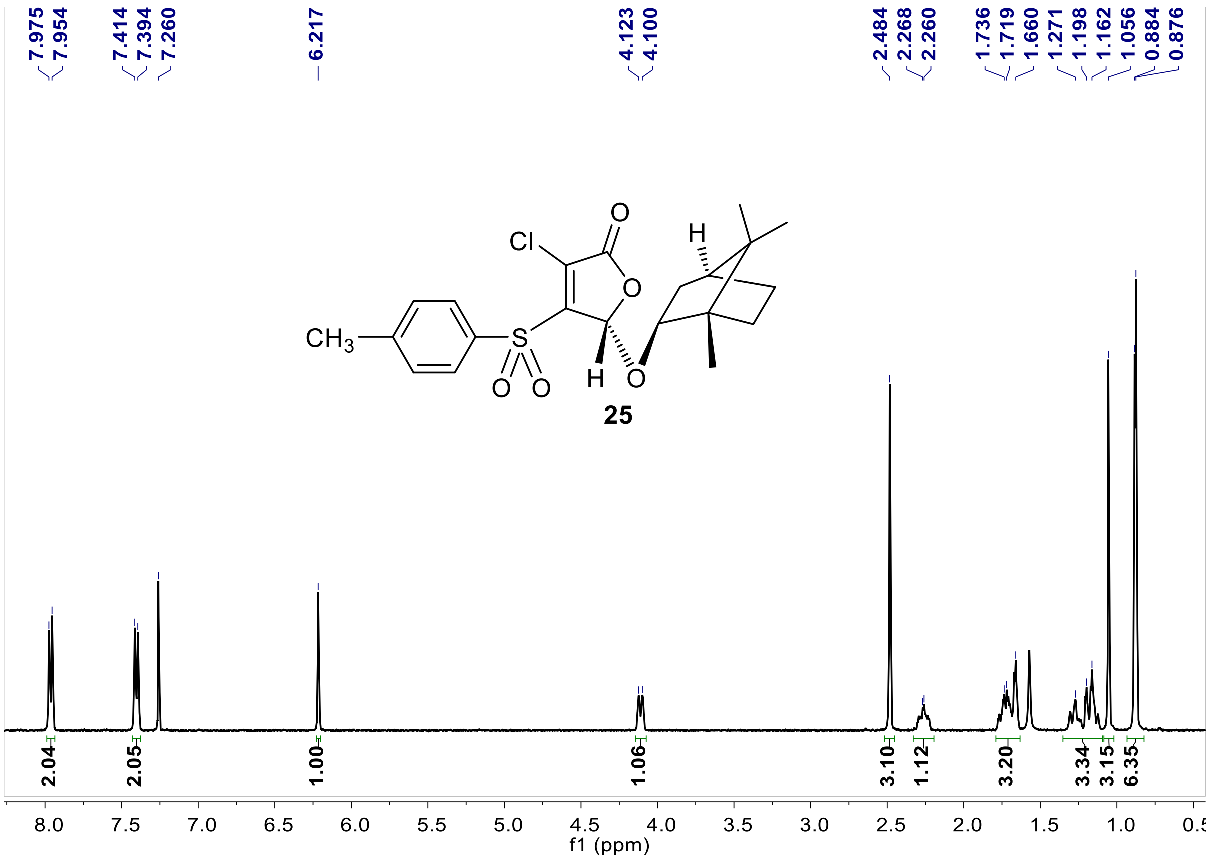

b)

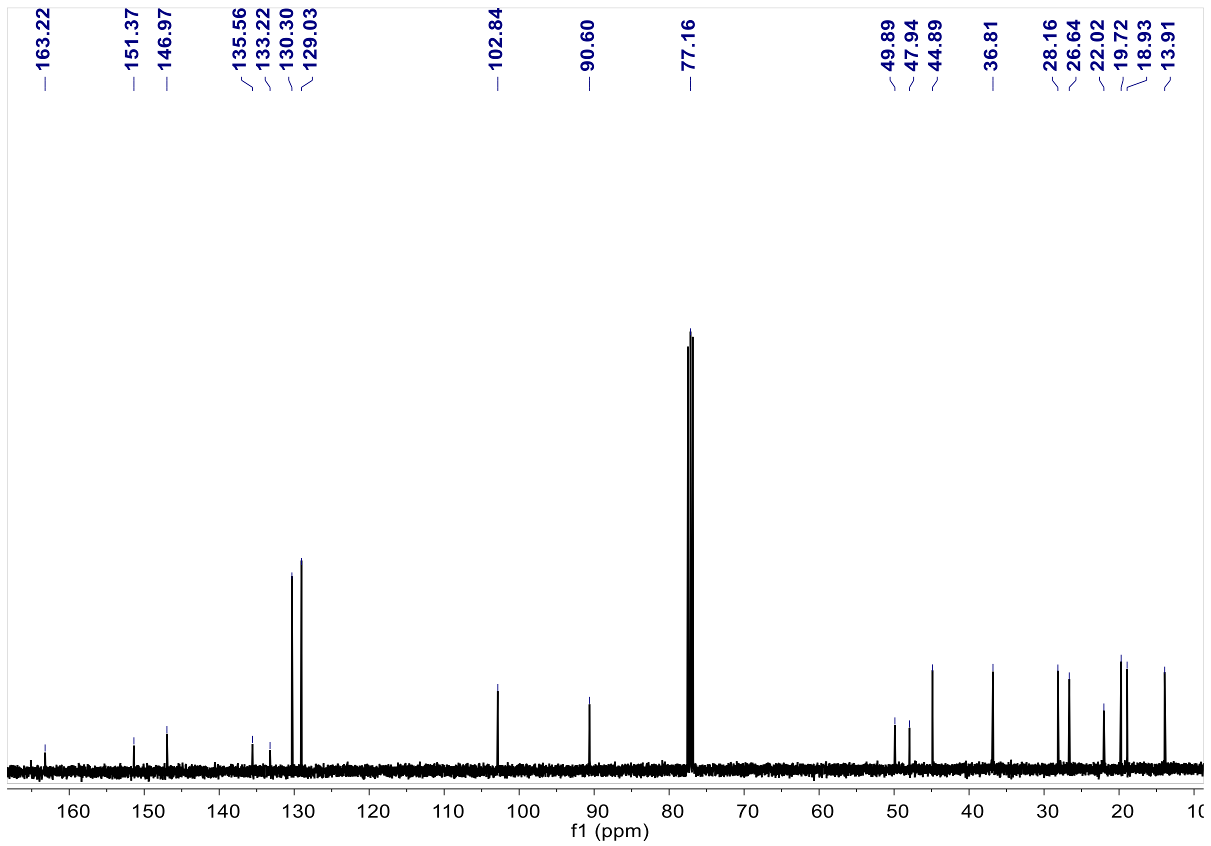

Figure S16. ^{1}H (a) and ^{13}C{^{1}H} (b) NMR spectra of compound **25** ($CDCl_3$).

a)

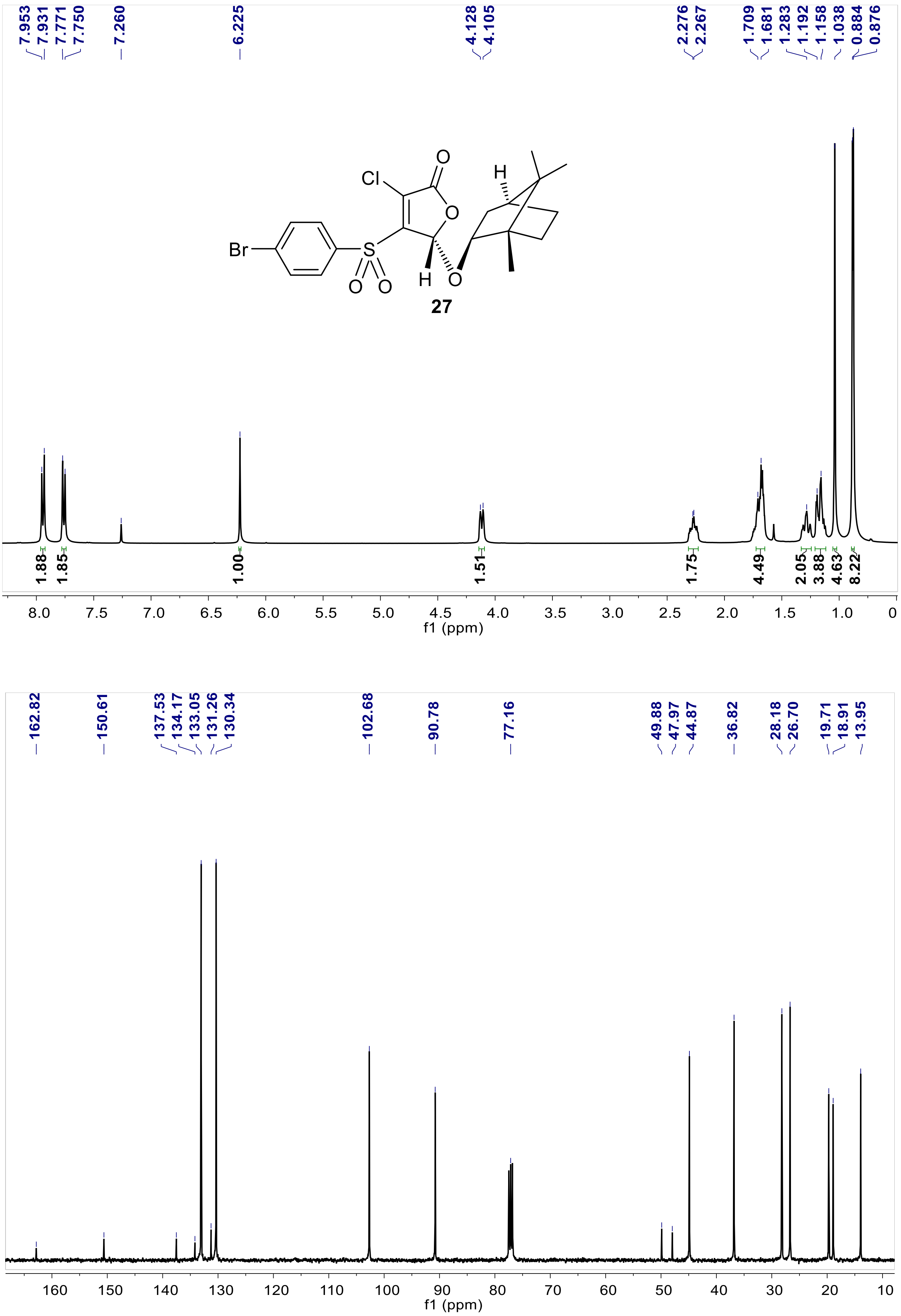

b)

Figure S17. ^{1}H (a) and $^{13}C\{^{1}H\}$ (b) NMR spectra of compound **27** ($CDCl_3$).

a)

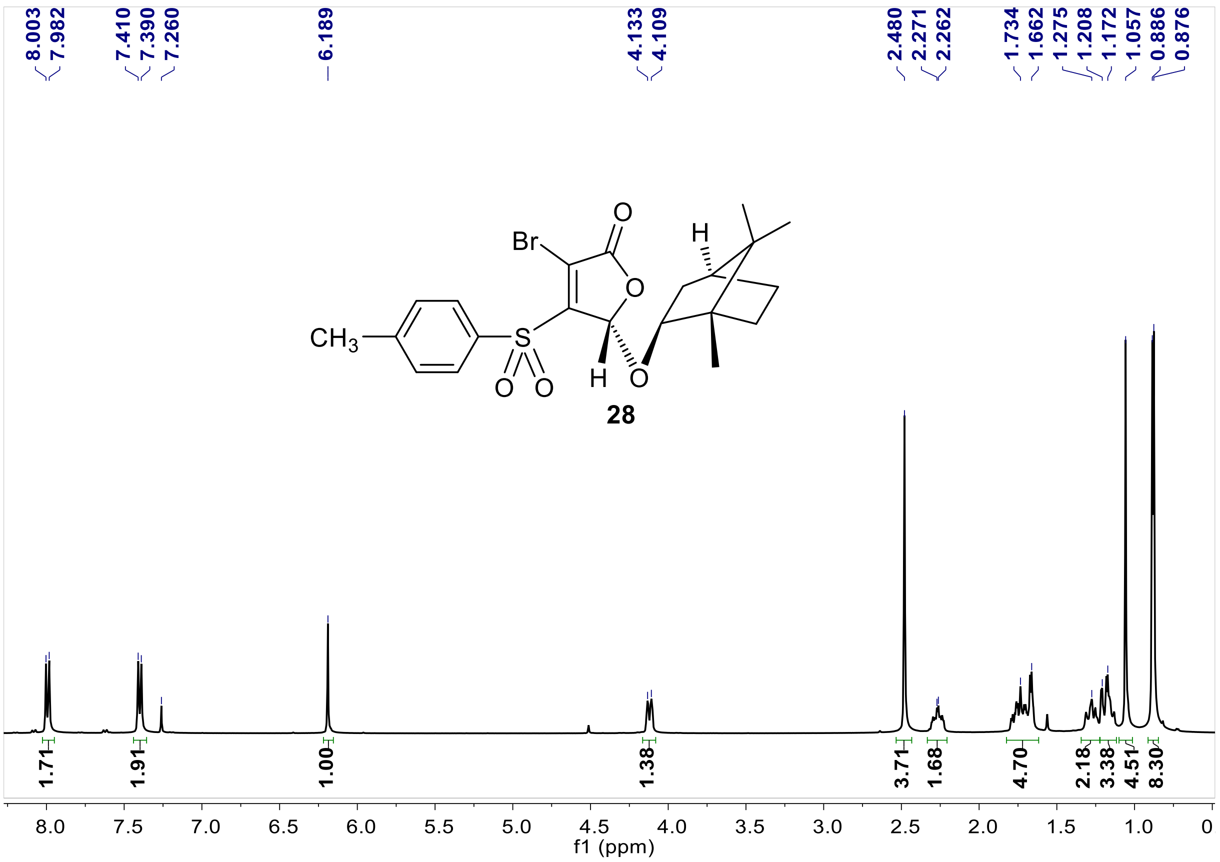

b)

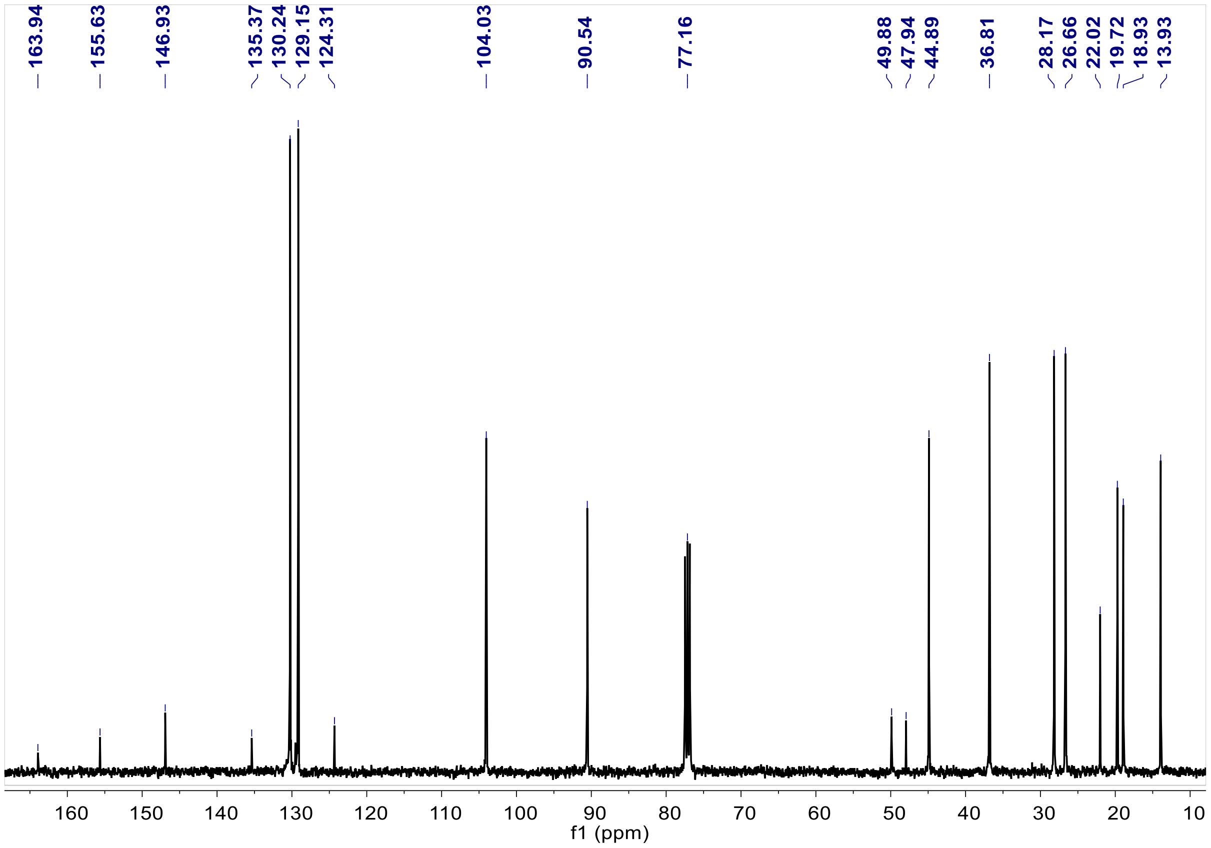

Figure S18. ^{1}H (a) and $^{13}C\{^{1}H\}$ (b) NMR spectra of compound **28** ($CDCl_3$).

a)

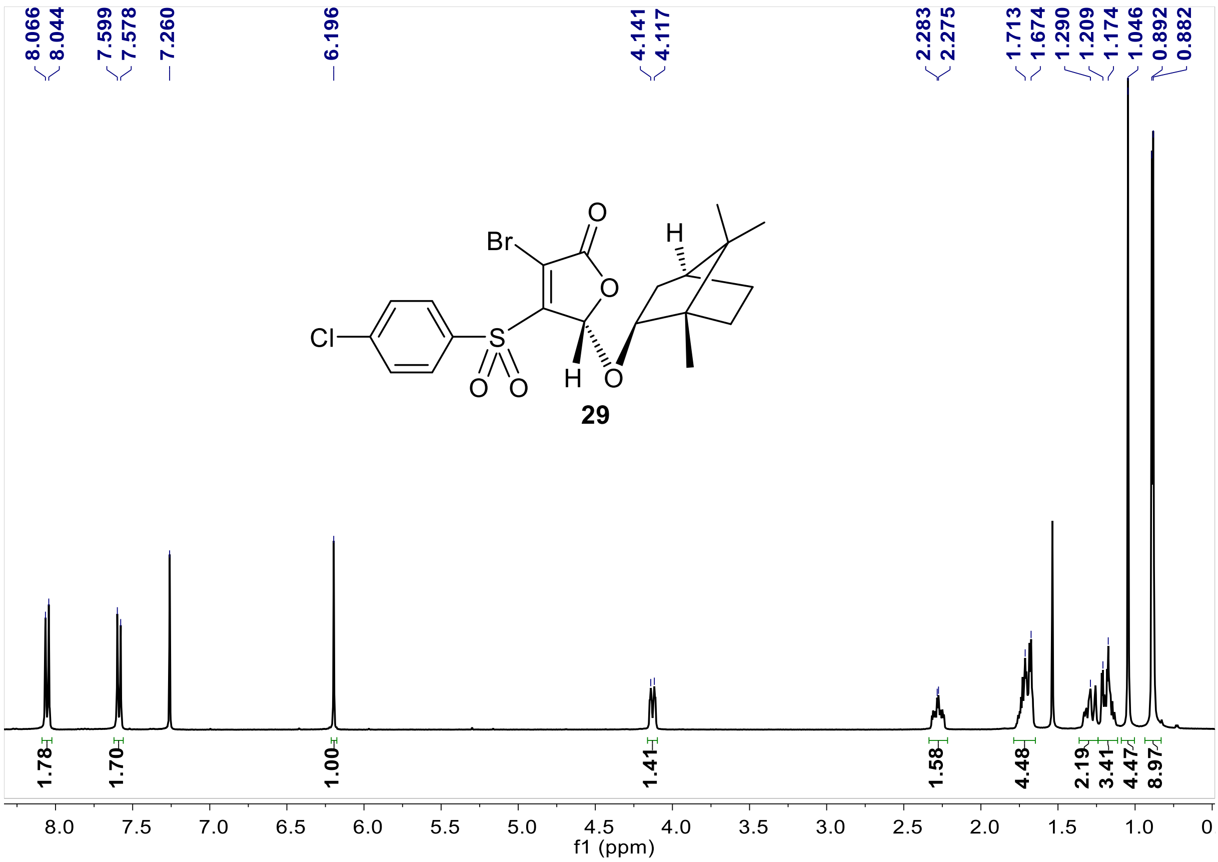

b)

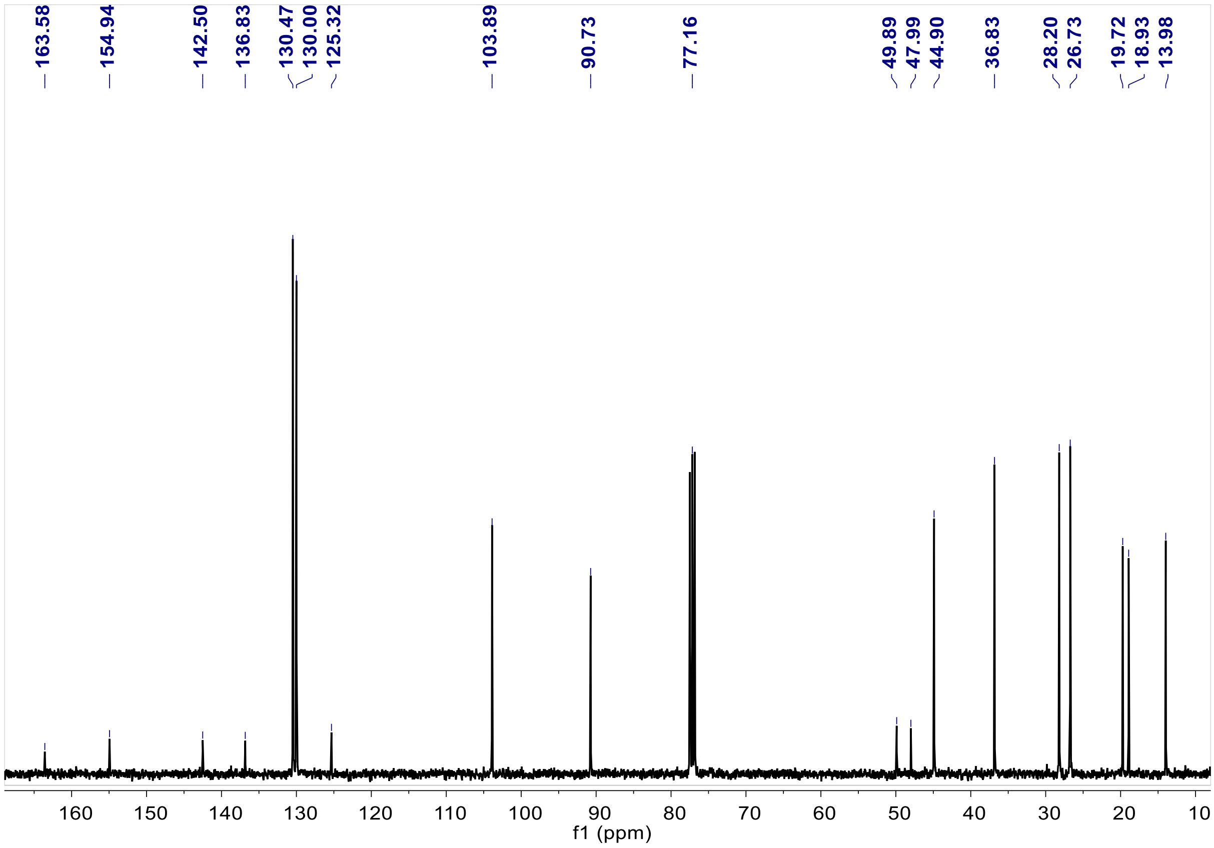

Figure S19. ^{1}H (a) and $^{13}C\{^{1}H\}$ (b) NMR spectra of compound **29** ($CDCl_3$).

a)

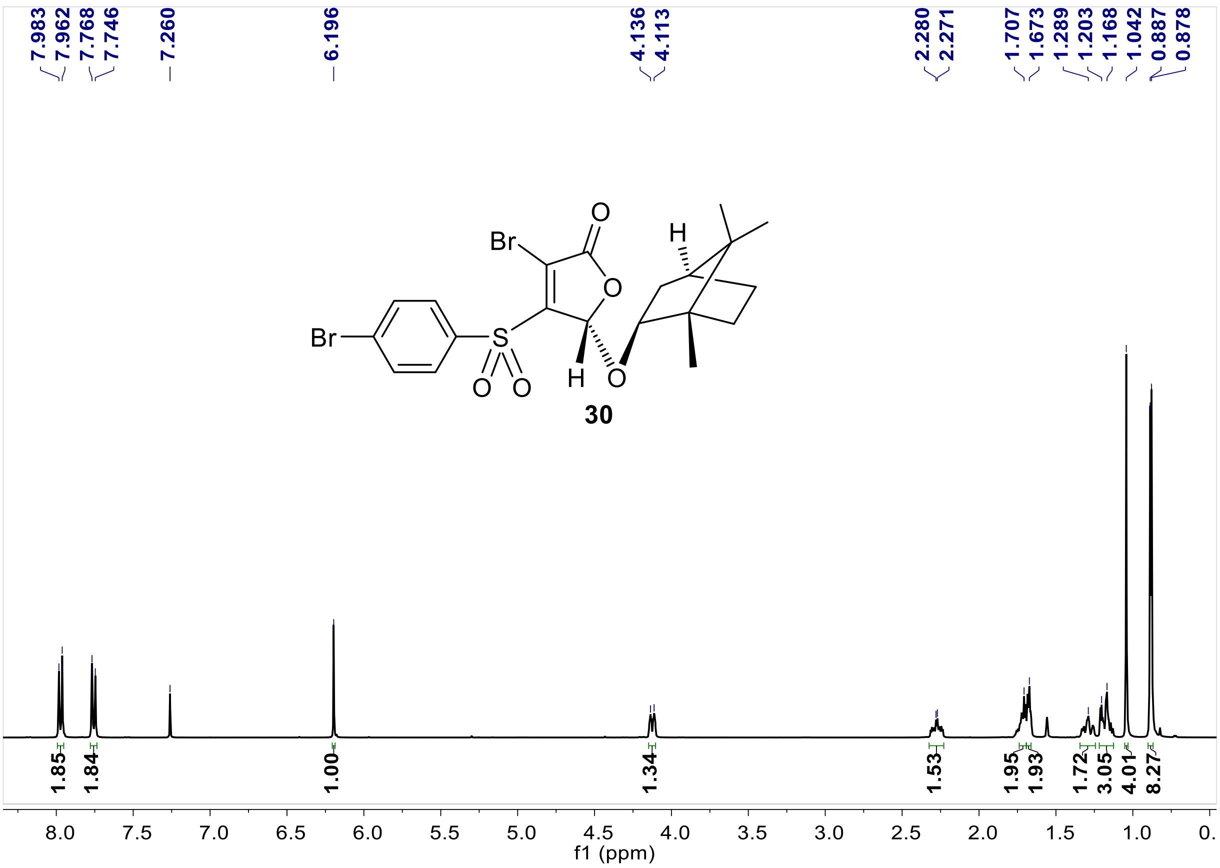

b)

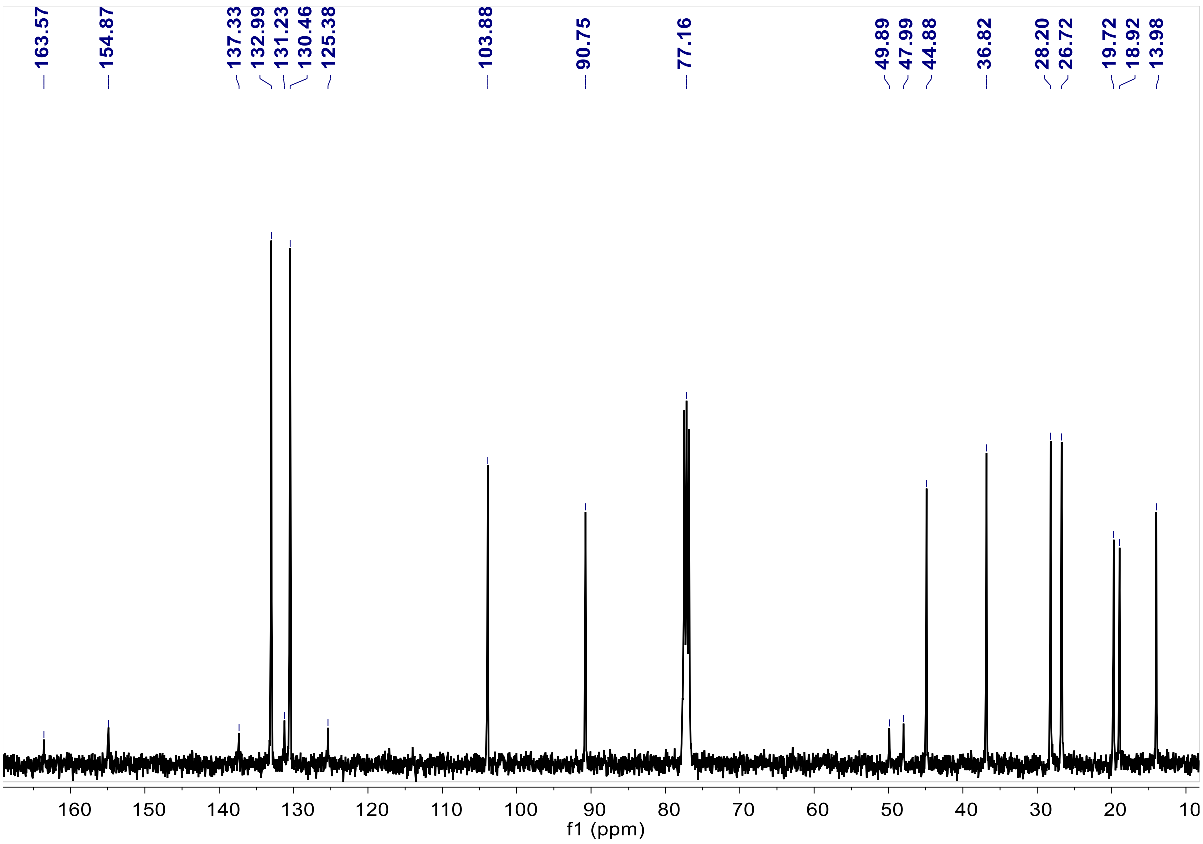

Figure S20. ^{1}H (a) and $^{13}C\{^{1}H\}$ (b) NMR spectra of compound **30** ($CDCl_3$).

IR spectra of thioethers

a)

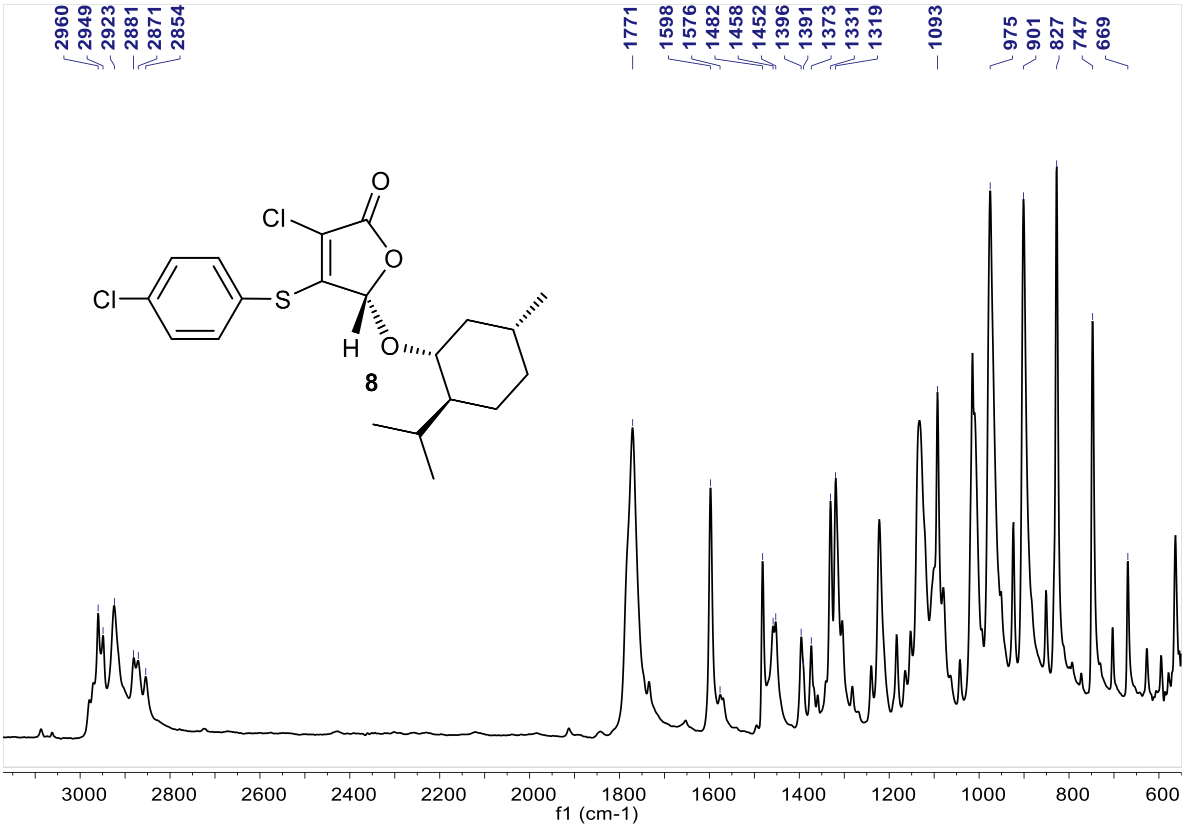

b)

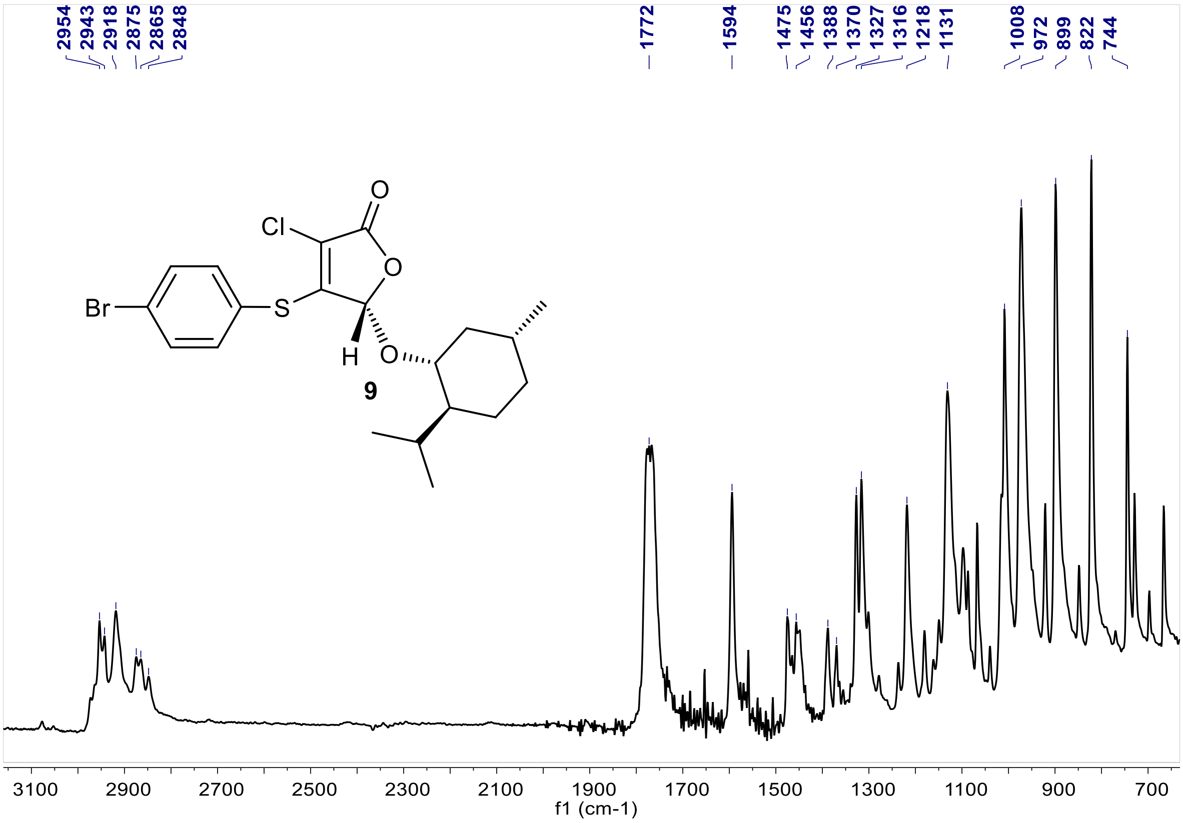

c)

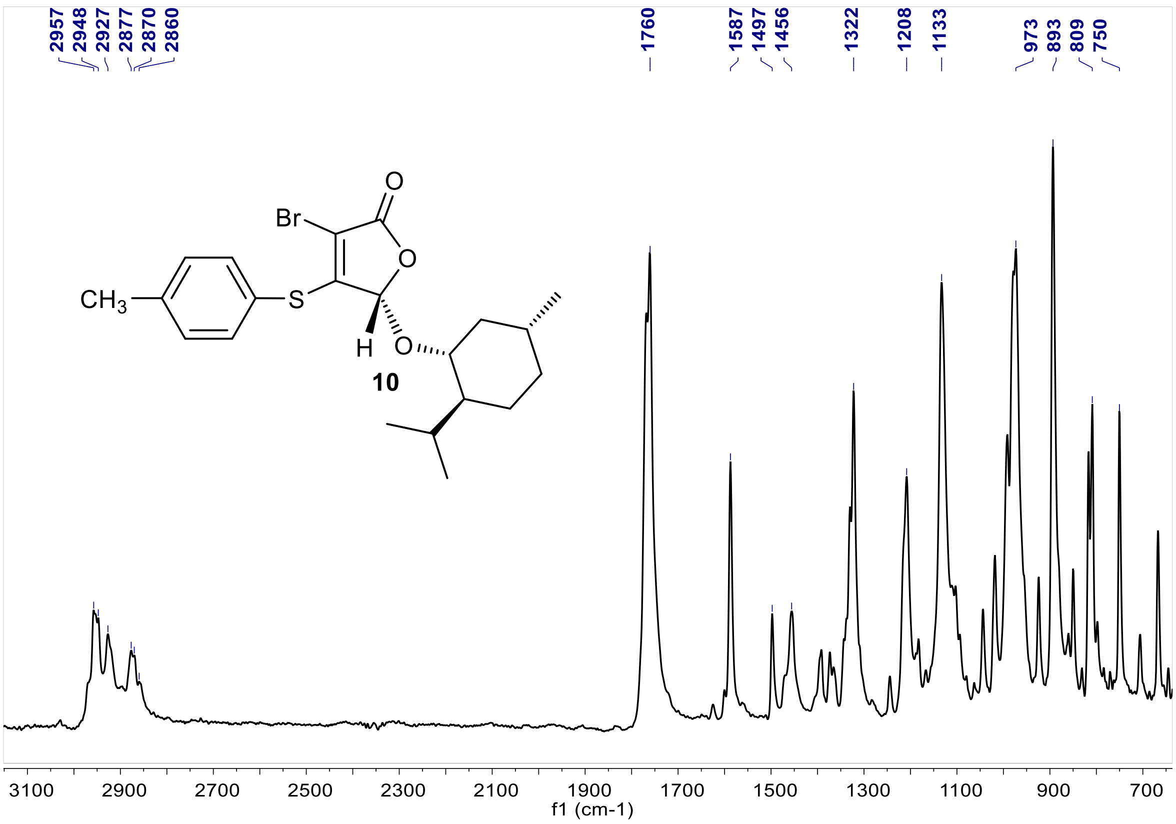
2957
2948
2927
2877
2870
2860
1760
1587
1497
1456
1322
1208
1133
973
893
809
750
Br
O
O
S
CH3
H
O
10
3100
2900
2700
2500
2300
2100
1900
1700
1500
1300
1100
900
700
f1 (cm-1)

d)

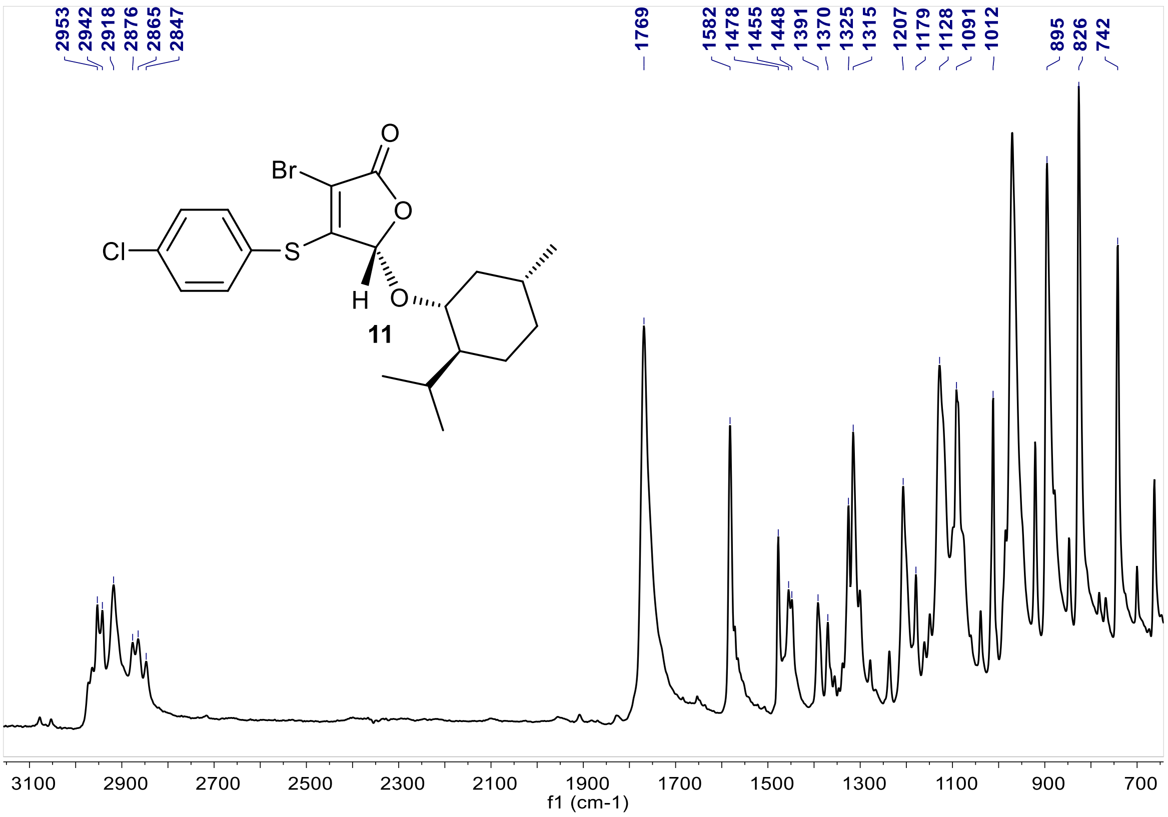
2953
2942
2918
2876
2865
2847
1769
1582
1478
1455
1448
1391
1370
1325
1315
1207
1179
1128
1091
1012
895
826
742
Br
O
O
S
Cl
H
O
11
3100
2900
2700
2500
2300
2100
1900
1700
1500
1300
1100
900
700
f1 (cm-1)

e)

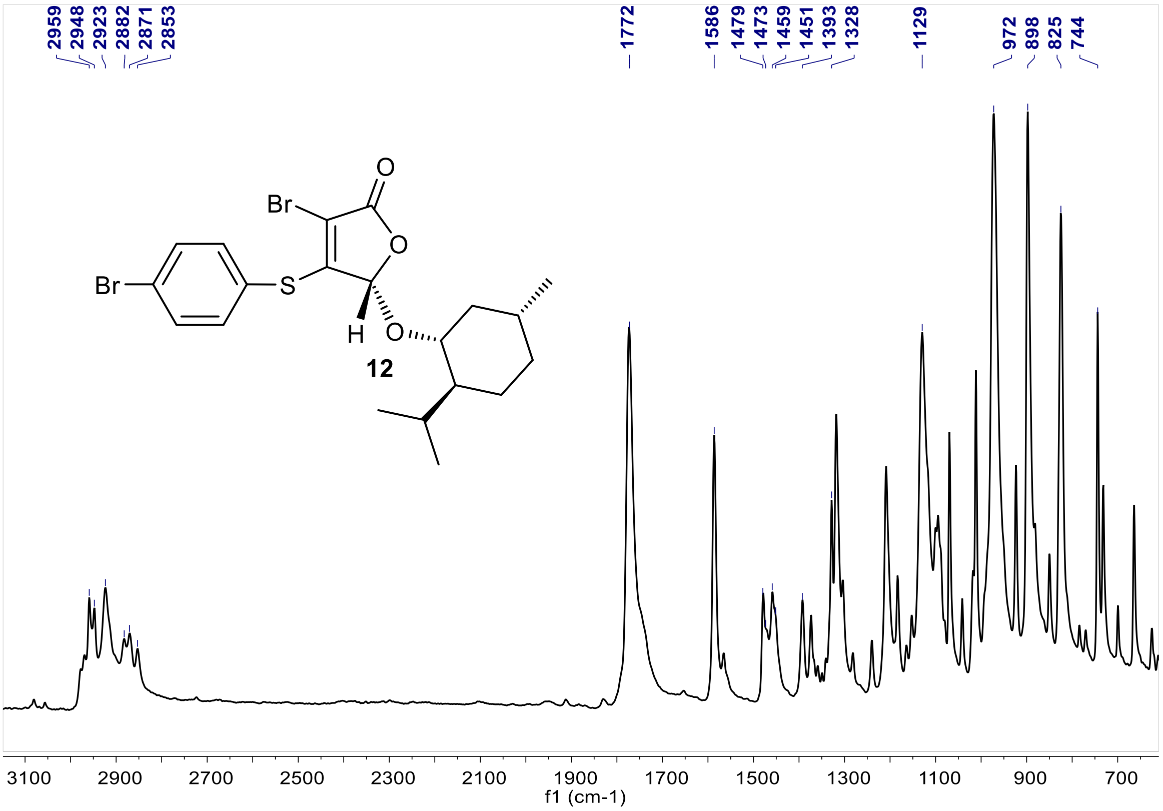
2959
2948
2923
2882
2871
2853
1772
1586
1479
1473
1459
1451
1393
1328
1129
972
898
825
744
Br
O
O
Br
S
H
O
12
3100 2900 2700 2500 2300 2100 1900 1700 1500 1300 1100 900 700
f1 (cm-1)

f)

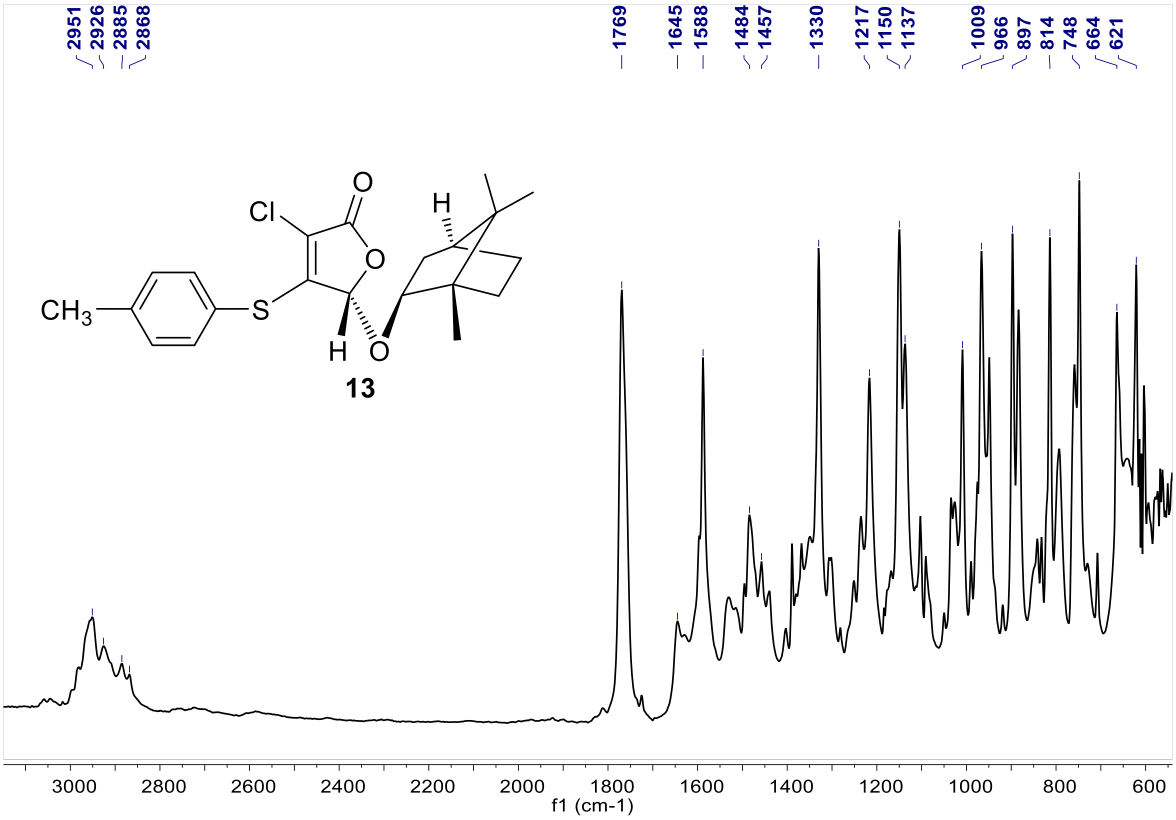
2951
2926
2885
2868
1769
1645
1588
1484
1457
1330
1217
1150
1137
1009
966
897
814
748
664
621
Cl
O
O
H
CH3
S
H
O
13
3000 2800 2600 2400 2200 2000 1800 1600 1400 1200 1000 800 600
f1 (cm-1)

g)

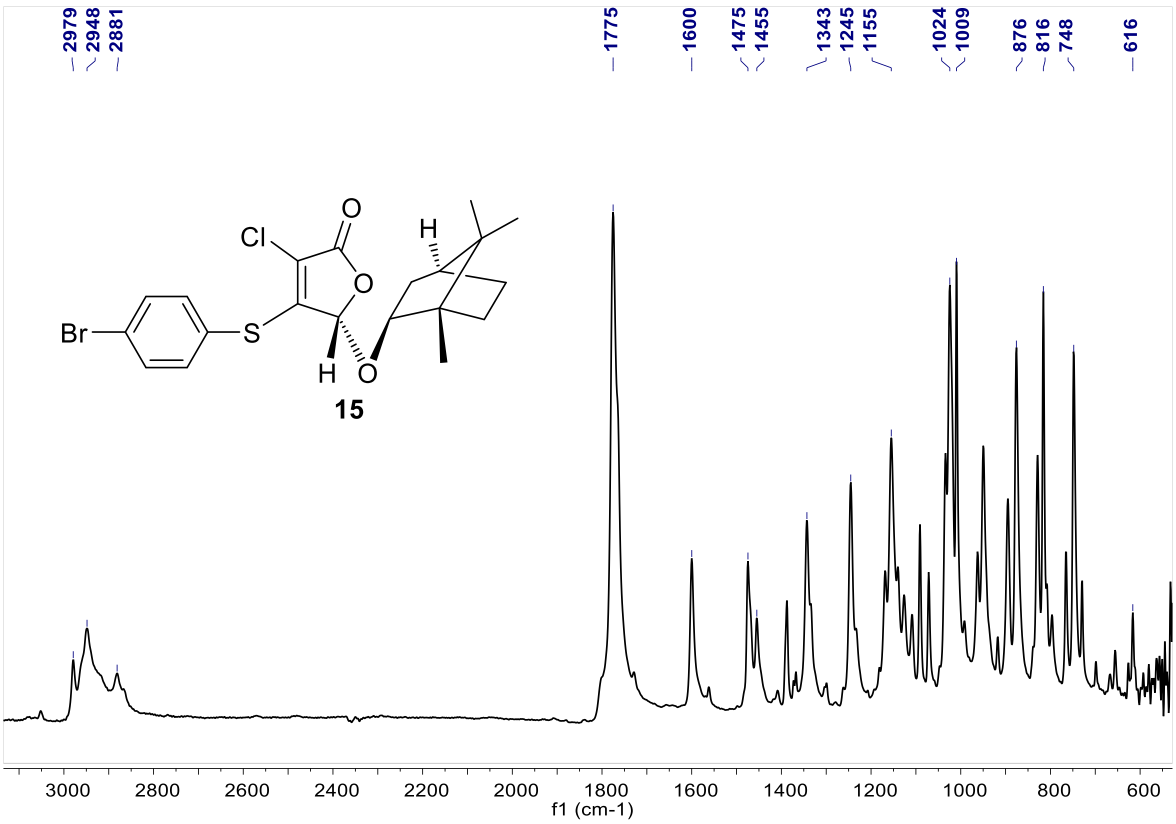
2979
2948
2881
1775
1600
1475
1455
1343
1245
1155
1024
1009
876
816
748
616
Cl
O
H
O
Br
S
H
O
15
3000
2800
2600
2400
2200
2000
1800
1600
1400
1200
1000
800
600
f1 (cm-1)

h)

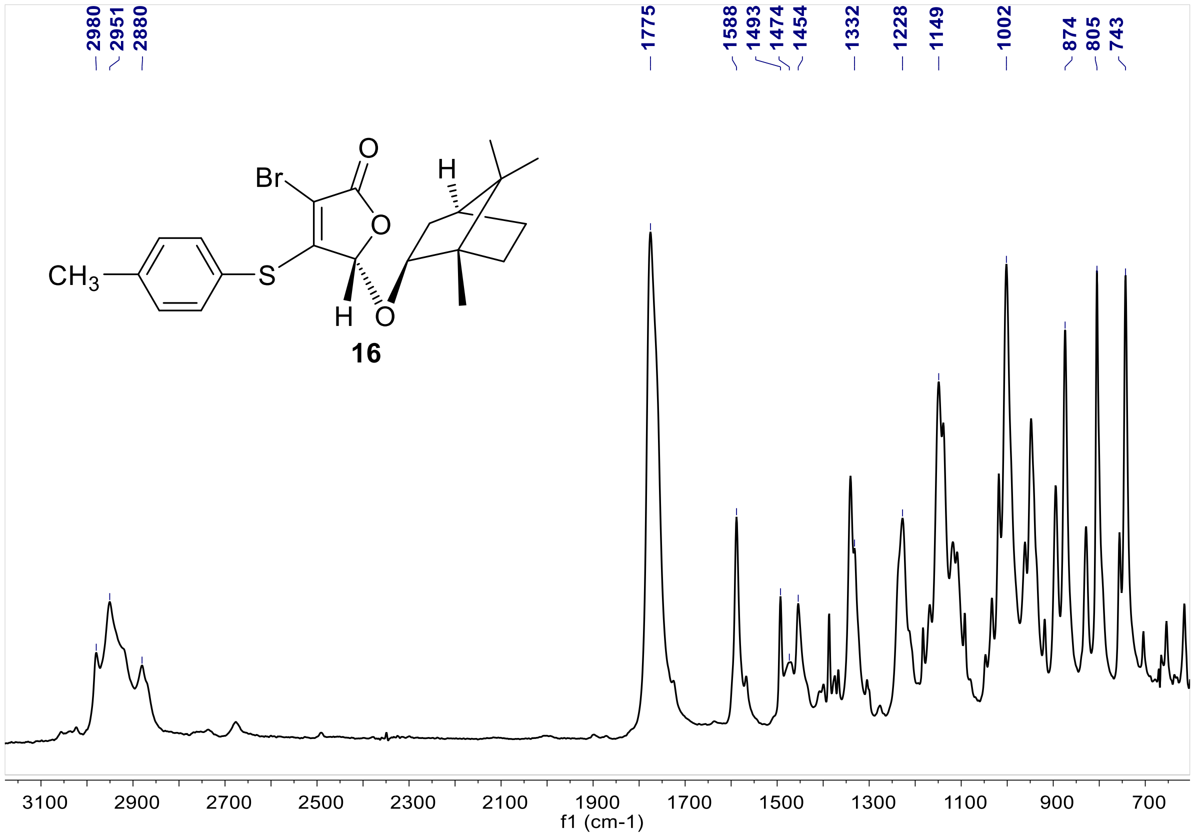
2980
2951
2880
1775
1588
1493
1474
1454
1332
1228
1149
1002
874
805
743
O
Br
H
O
CH_3
S
H
O
16
3100
2900
2700
2500
2300
2100
1900
1700
1500
1300
1100
900
700
f1 (cm-1)

i)

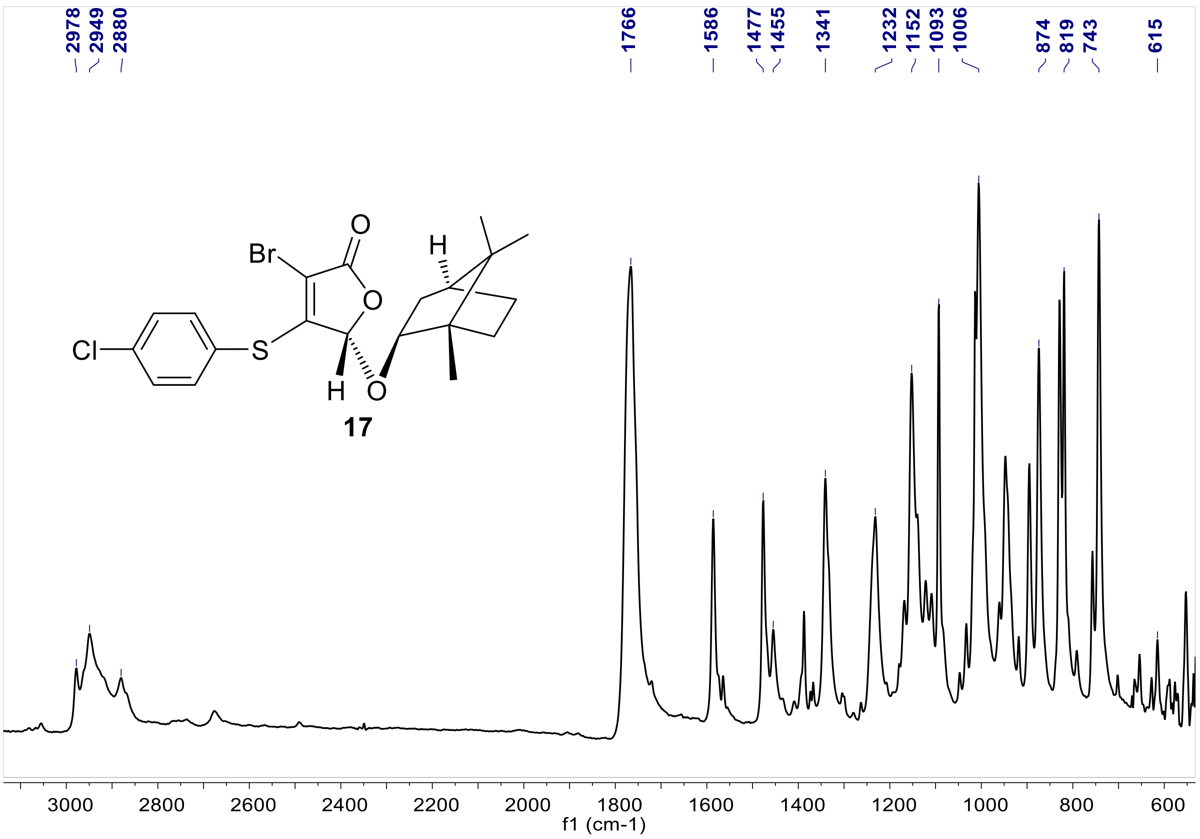

j)

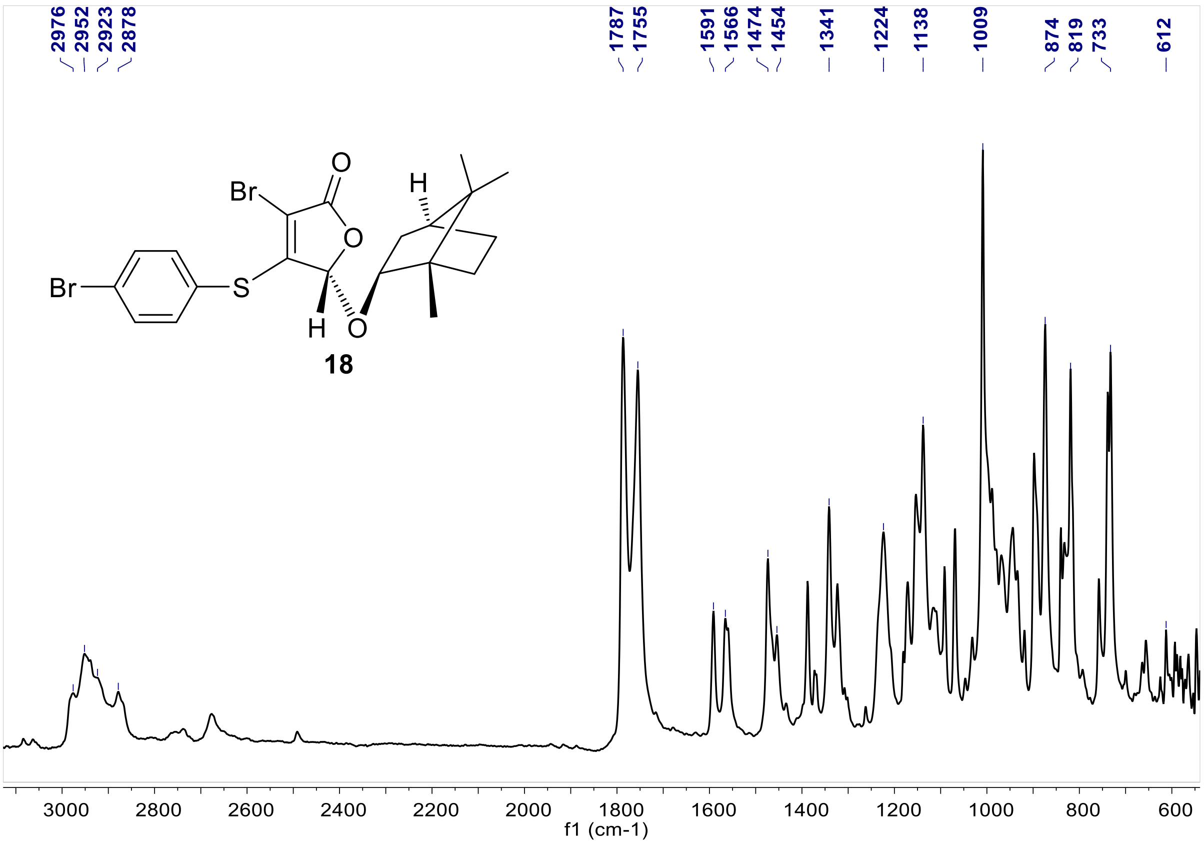

Figure S21. IR spectra of thioethers **8**–**18**.

IR spectra of sulfones

a)

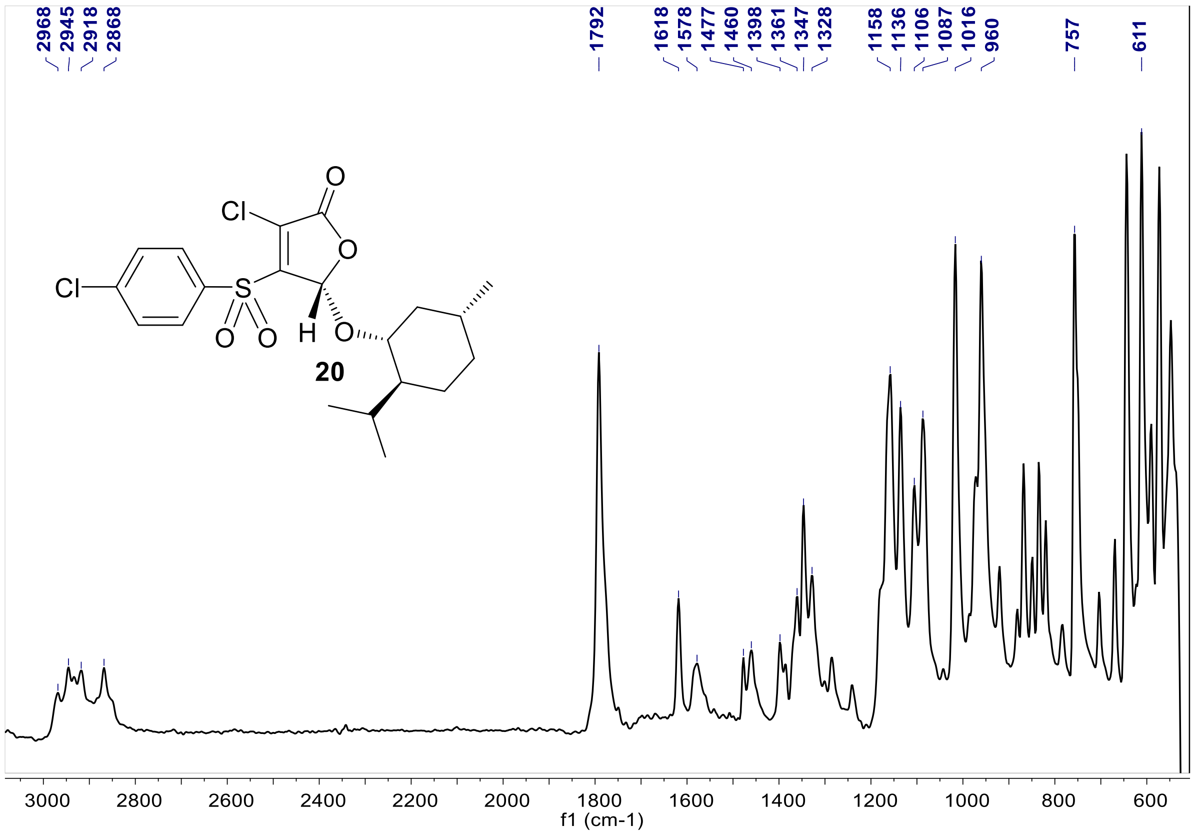

b)

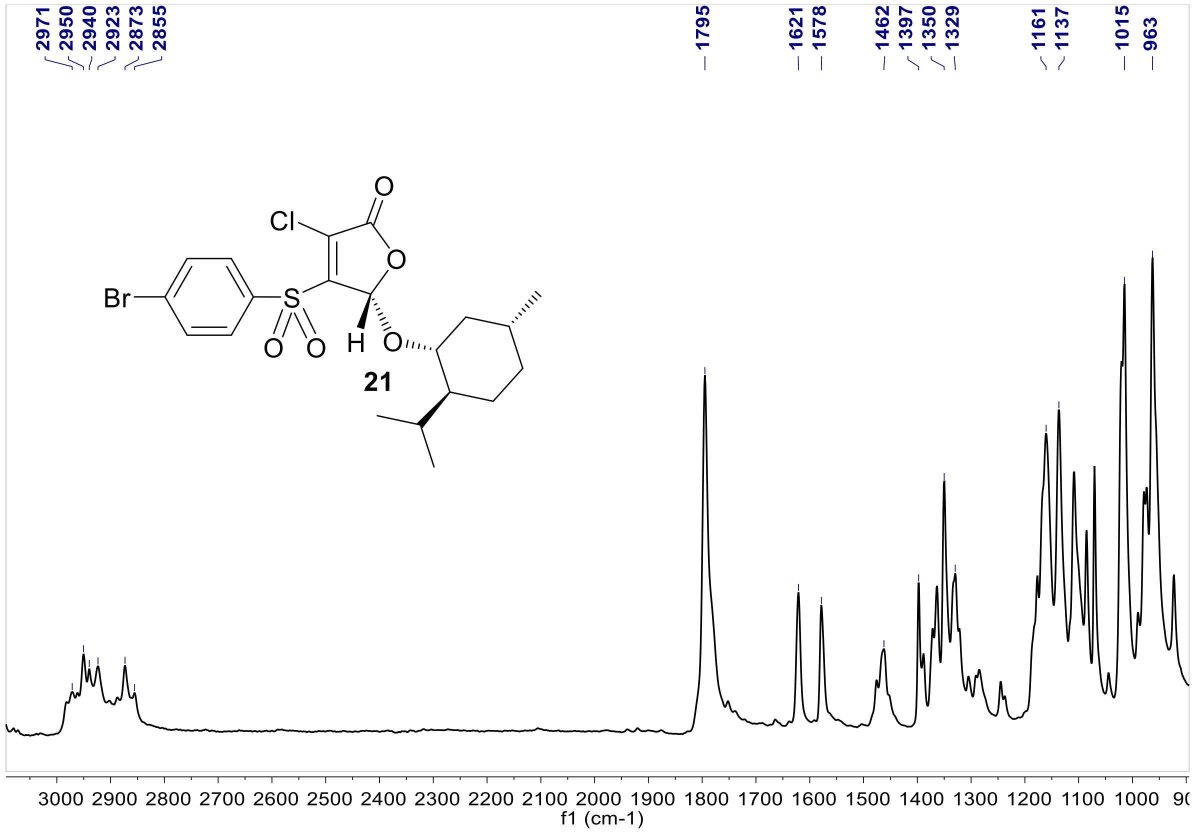

c)

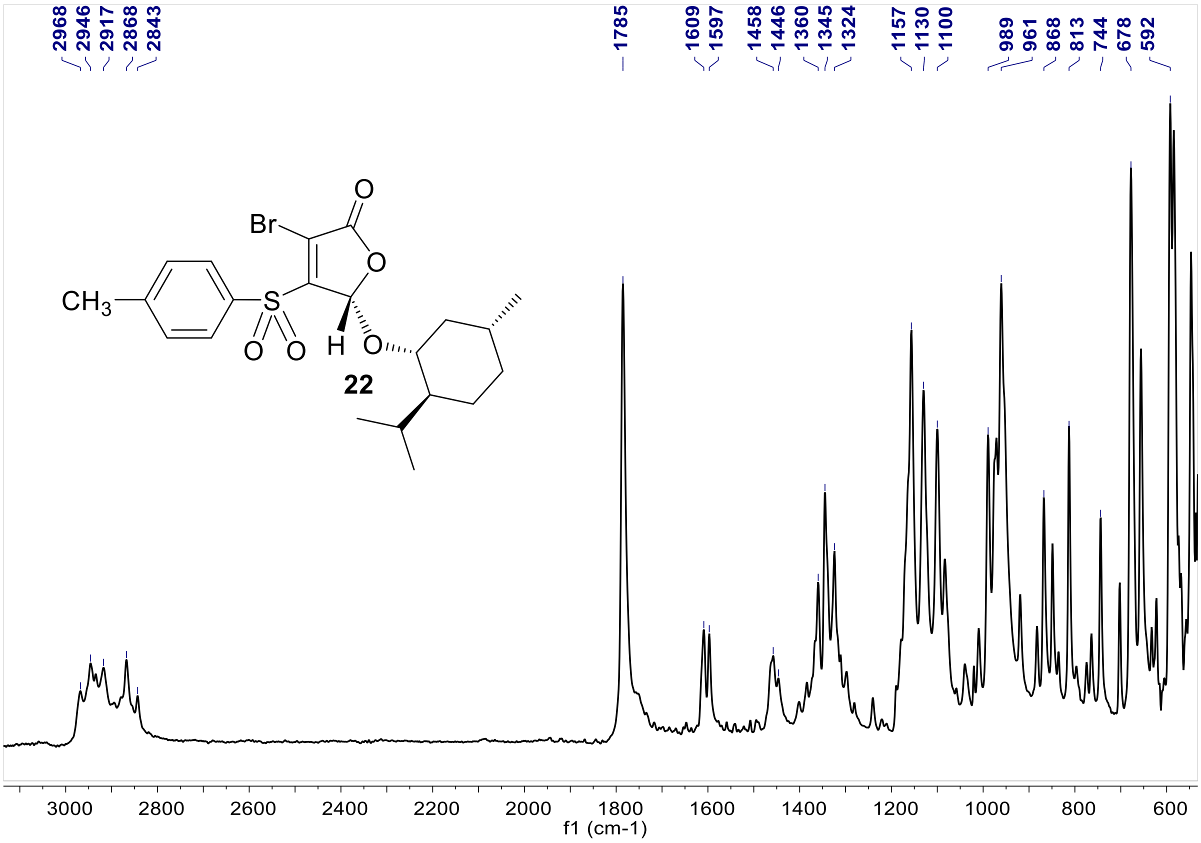
2968
2946
2917
2868
2843
1785
1609
1597
1458
1446
1360
1345
1324
1157
1130
1100
989
961
868
813
744
678
592
Br
O
O
CH3
S
O
O
H
O
22
3000 2800 2600 2400 2200 2000 1800 1600 1400 1200 1000 800 600
f1 (cm-1)

d)

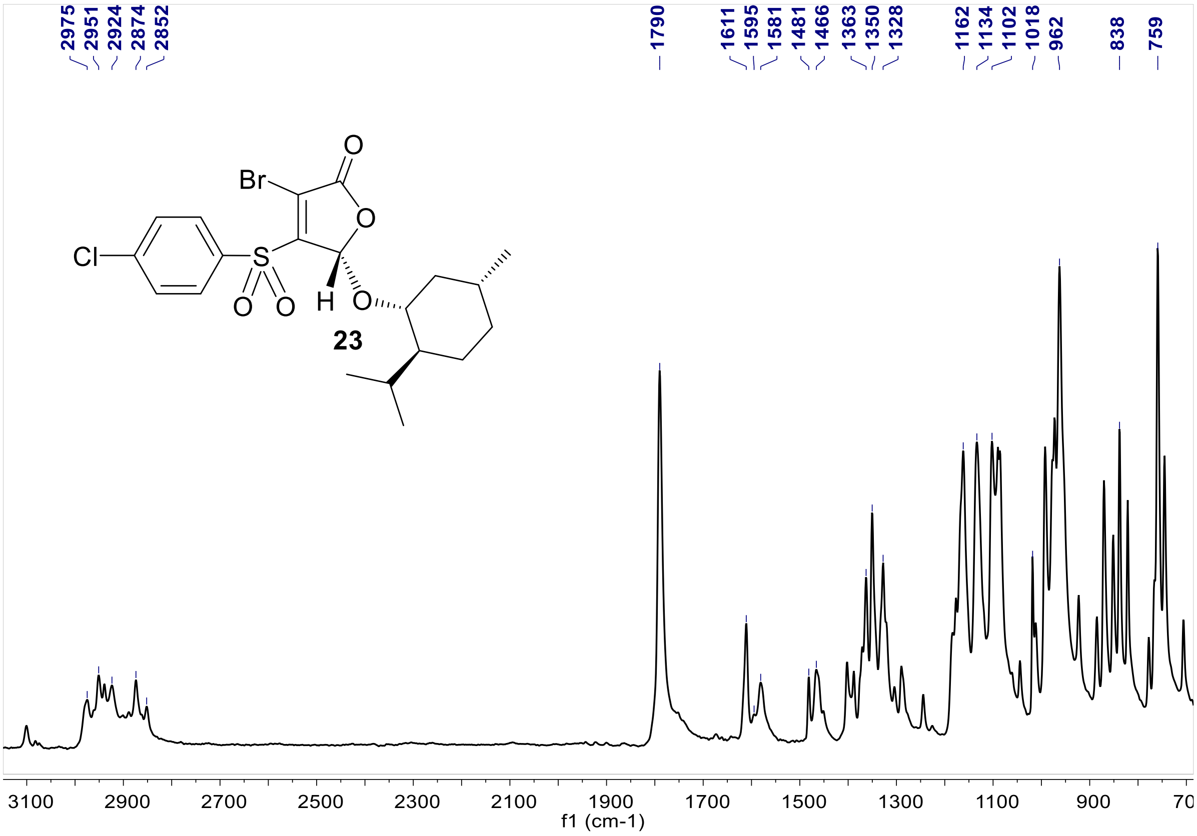
2975
2951
2924
2874
2852
1790
1611
1595
1581
1481
1466
1363
1350
1328
1162
1134
1102
1018
962
838
759
Br
O
O
Cl
S
O
O
H
O
23
3100 2900 2700 2500 2300 2100 1900 1700 1500 1300 1100 900 70
f1 (cm-1)

e)

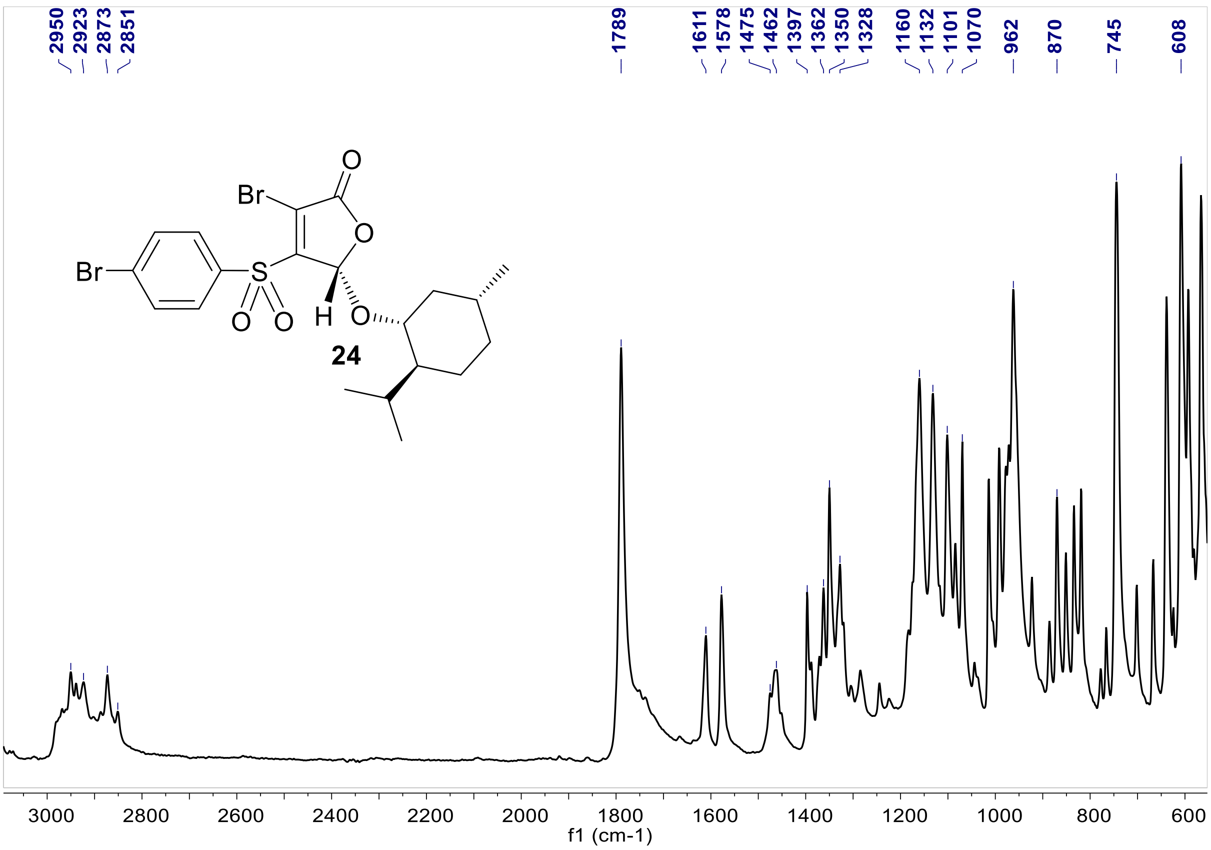
2950
2923
2873
2851
1789
1611
1578
1475
1462
1397
1362
1350
1328
1160
1132
1101
1070
962
870
745
608
Br
Br
S
O
O
O
O
H
O
24
3000 2800 2600 2400 2200 2000 1800 1600 1400 1200 1000 800 600
f1 (cm-1)

f)

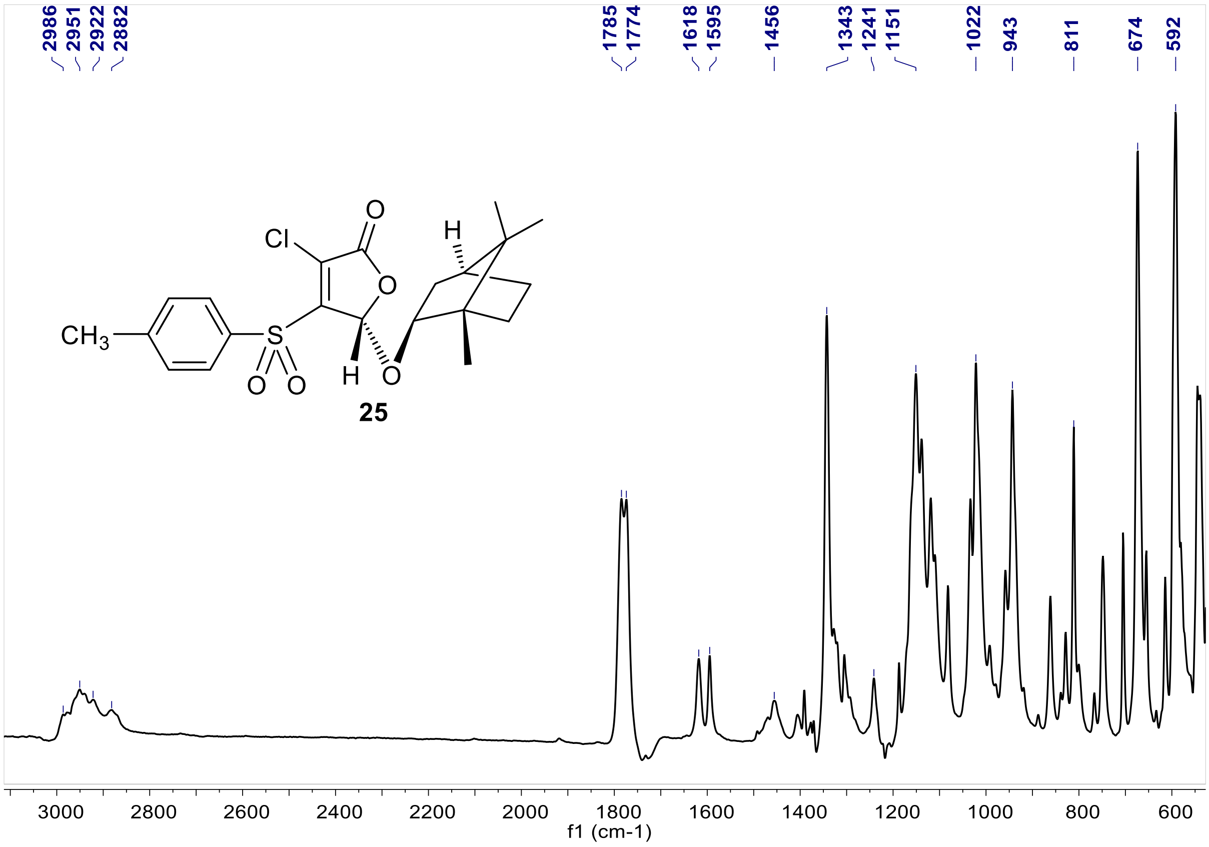
2986
2951
2922
2882
1785
1774
1618
1595
1456
1343
1241
1151
1022
943
811
674
592
Cl
CH3
S
O
O
O
O
H
O
H
25
3000 2800 2600 2400 2200 2000 1800 1600 1400 1200 1000 800 600
f1 (cm-1)

g)

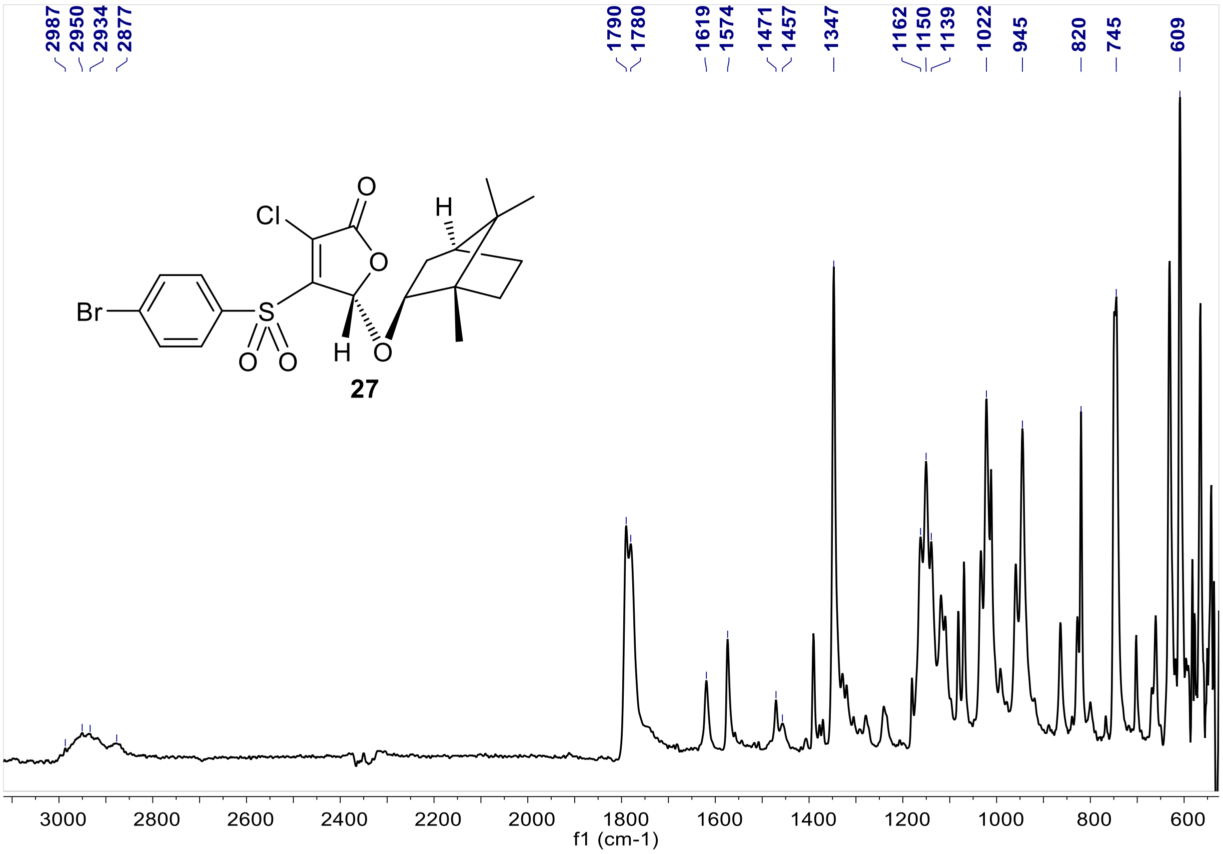
2987
2950
2934
2877
1790
1780
1619
1574
1471
1457
1347
1162
1150
1139
1022
945
820
745
609
Cl
O
O
H
Br
S
O
O
H
O
27
3000
2800
2600
2400
2200
2000
1800
1600
1400
1200
1000
800
600
f1 (cm-1)

h)

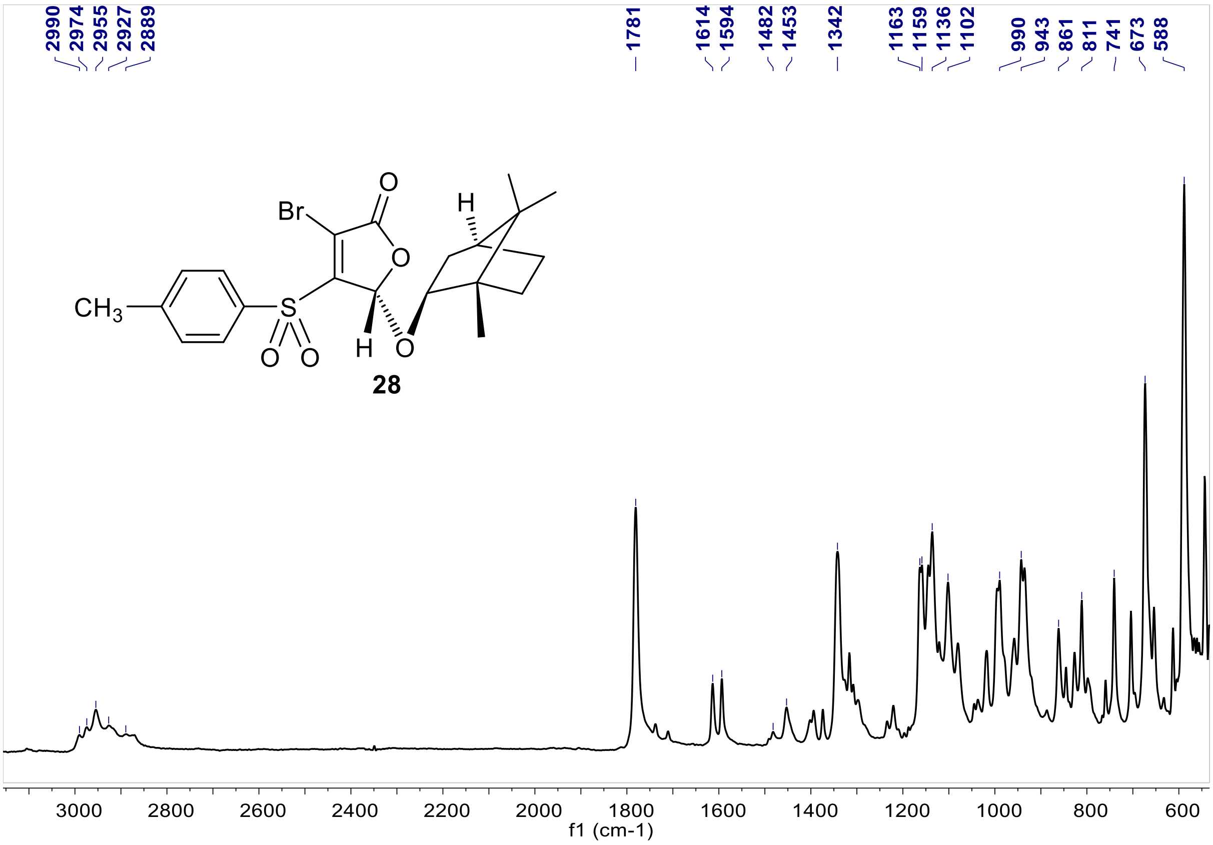
2990
2974
2955
2927
2889
1781
1614
1594
1482
1453
1342
1163
1159
1136
1102
990
943
861
811
741
673
588
Br
O
O
H
CH3
S
O
O
H
O
28
3000
2800
2600
2400
2200
2000
1800
1600
1400
1200
1000
800
600
f1 (cm-1)

i)

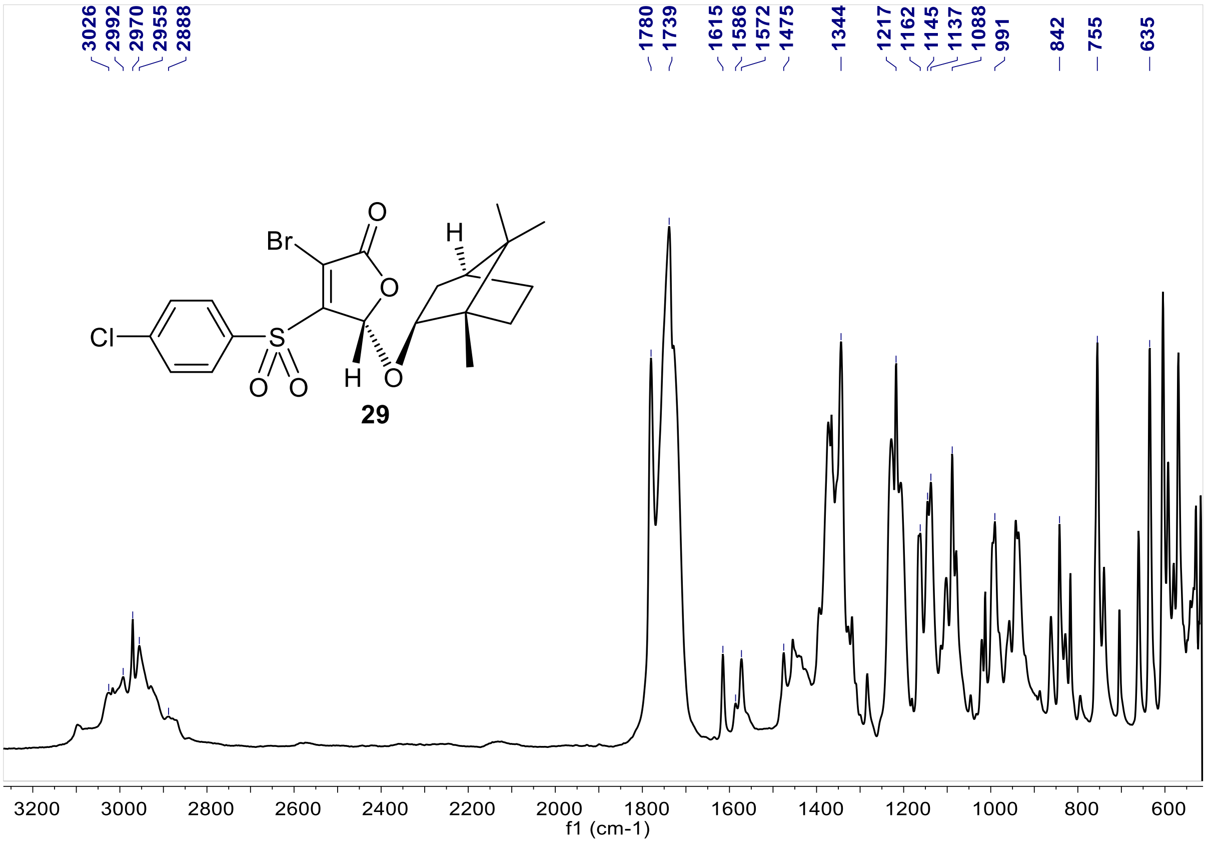

j)

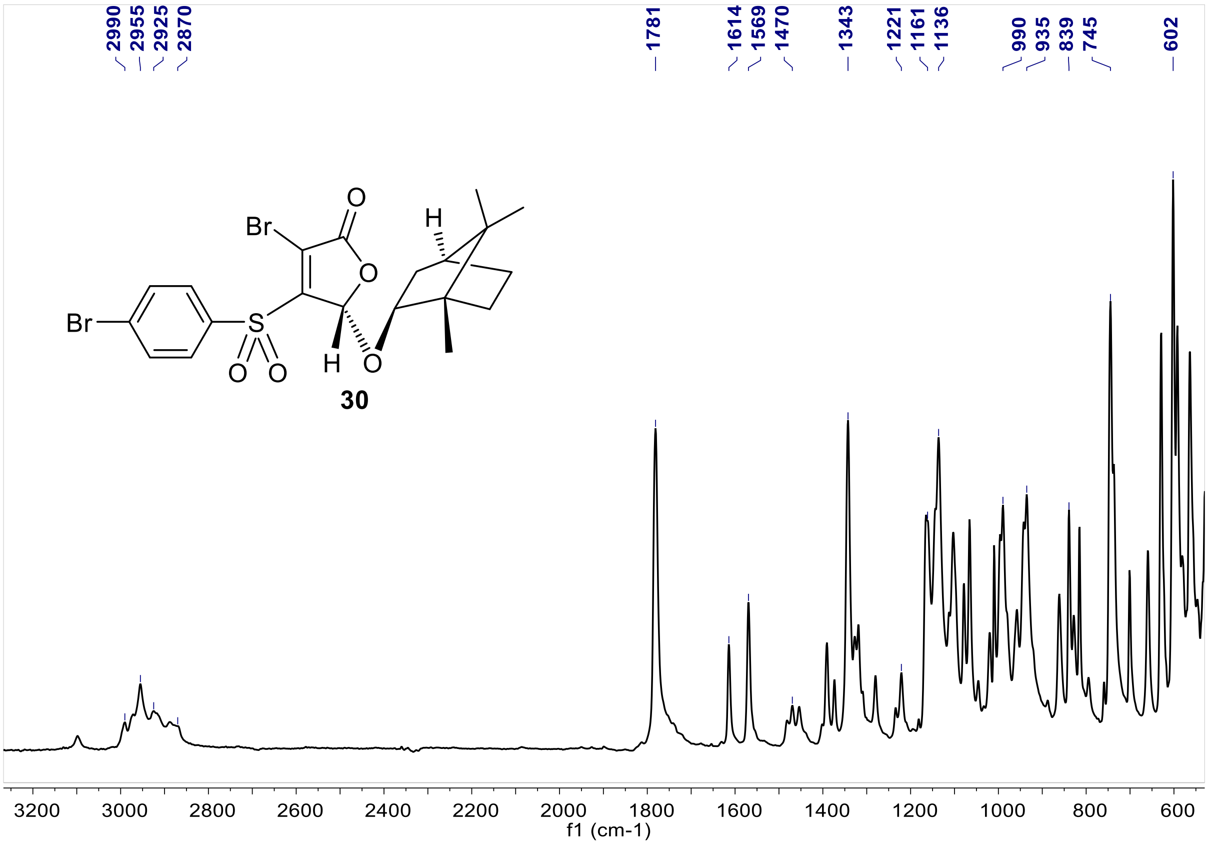

Figure S22. IR spectra of sulfones **20**–**30**.

Mass spectra of thioethers

a)

Mass Spectrum SmartFormula Report

Analysis Info

Acquisition Date 2018/04/19 15:25:52

Analysis Name D:\Data\User

Method lcms_esi_pos_low.m

Operator BDAL@DE

Sample Name HcC1

Instrument micrOTOF II 8213750.10448

Comment

Acquisition Parameter

Source Type	ESI	Ion Polarity	Positive	Set Nebulizer	3.0 Bar
Focus	Not active			Set Dry Heater	200 • ‹C
Scan Begin	50 m/z	Set Capillary	4500 V	Set Dry Gas	10.0 l/min
Scan End	2000 m/z	Set End Plate Offset	-500 V	Set Divert Valve	Waste

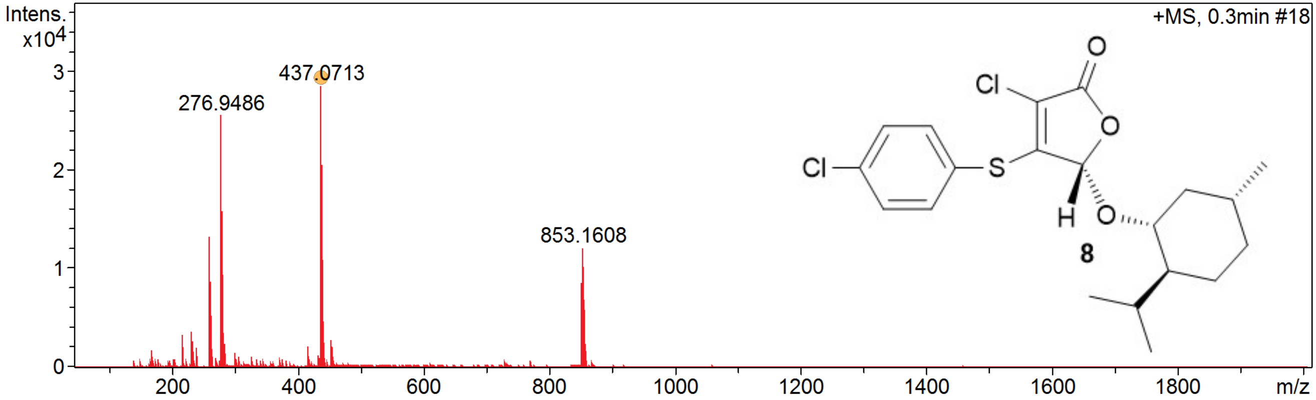

Meas. m/z	#	Ion Formula	m/z	err [ppm]	mSigma	# mSigma	Score	rdb	e• P Conf	N-Rule
437.0713	1	C20H24Cl2NaO3S	437.0715	0.6	5.4	1	100.00	7.5	even	ok

b)

Mass Spectrum SmartFormula Report

Analysis Info

Analysis Name	D:\Data\User	Acquisition Date	2018/03/03 15:22:04
Method	lcms_esi_pos_low.m	Operator	BDAL@DE
Sample Name	ZR-C1	Instrument	micrOTOF II 8213750.10448
Comment			

Acquisition Parameter

Source Type	ESI	Ion Polarity	Positive	Set Nebulizer	3.0 Bar
Focus	Not active			Set Dry Heater	200 • ‹C
Scan Begin	50 m/z	Set Capillary	4500 V	Set Dry Gas	10.0 l/min
Scan End	2000 m/z	Set End Plate Offset	-500 V	Set Divert Valve	Waste

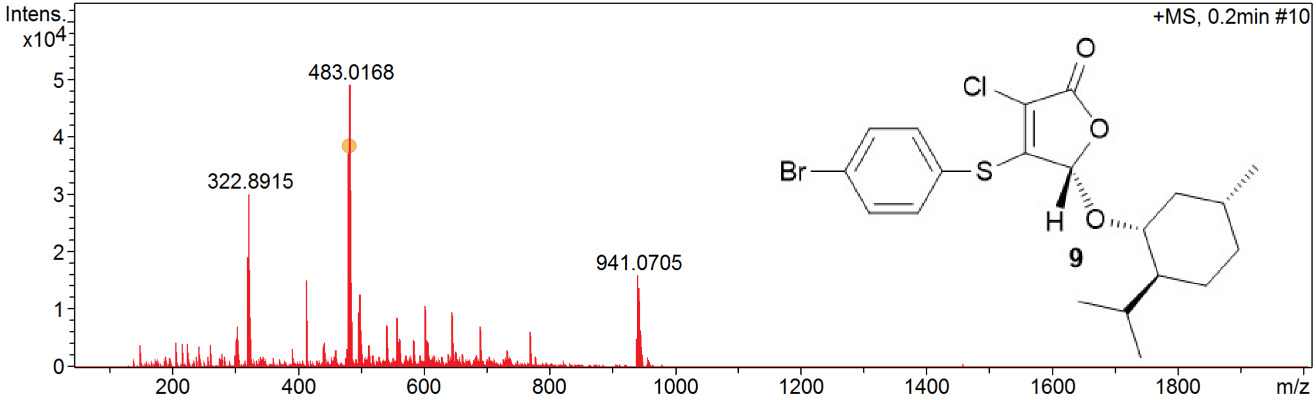

9

Meas. m/z	#	Ion Formula	m/z	err [ppm]	mSigma	# mSigma	Score	rdb	e• P Conf	N-Rule
481.0208	1	C20H24BrClNaO3S	481.0210	0.4	17.9	1	100.00	7.5	even	ok

c)

Mass Spectrum SmartFormula Report

Analysis Info

Analysis Name	D:\Data\User	Acquisition Date	2018/03/13 14:35:50
Method	lcms_esi_pos_low.m	Operator	BDAL@DE
Sample Name	ZR-K2	Instrument	micrOTOF II 8213750.10448
Comment			

Acquisition Parameter

Source Type	ESI	Ion Polarity	Positive	Set Nebulizer	3.0 Bar
Focus	Not active			Set Dry Heater	200 • ‹C
Scan Begin	50 m/z	Set Capillary	4500 V	Set Dry Gas	10.0 l/min
Scan End	2000 m/z	Set End Plate Offset	-500 V	Set Divert Valve	Waste

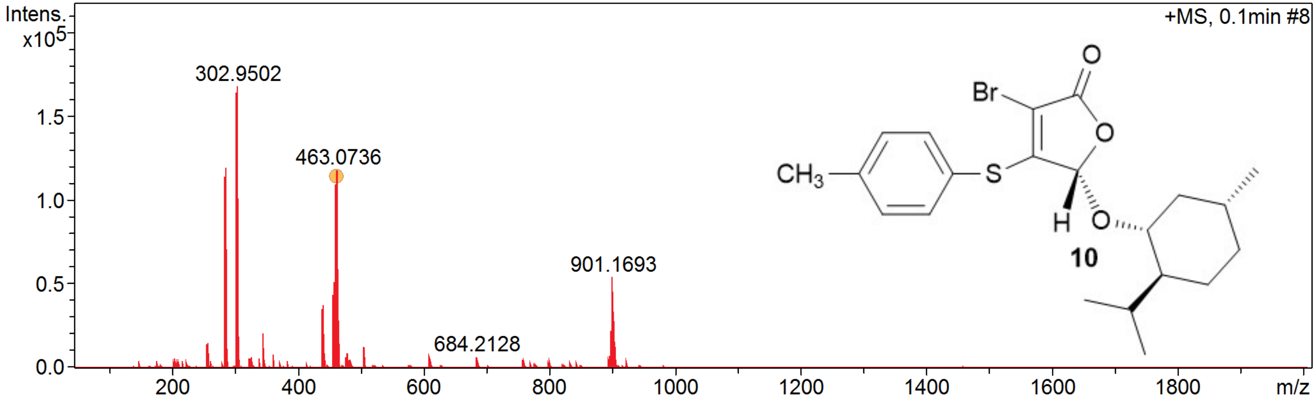

Meas. m/z	#	Ion Formula	m/z	err [ppm]	mSigma	# mSigma	Score	rdb	e• P Conf	N-Rule
461.0762	1	C21H27BrNaO3S	461.0756	-1.3	9.5	1	100.00	7.5	even	ok

d)

Mass Spectrum SmartFormula Report

Analysis Info

Analysis Name D:\Data\User
Method lcms_esi_pos_low.m
Sample Name ZR-D1
Comment

Acquisition Date 2018/03/03 15:22:04

Operator BDAL@DE
Instrument micrOTOF II 8213750.10448

Acquisition Parameter

Source Type	ESI	Ion Polarity	Positive	Set Nebulizer	3.0 Bar
Focus	Not active			Set Dry Heater	200 • ‹C
Scan Begin	50 m/z	Set Capillary	4500 V	Set Dry Gas	10.0 l/min
Scan End	2000 m/z	Set End Plate Offset	-500 V	Set Divert Valve	Waste

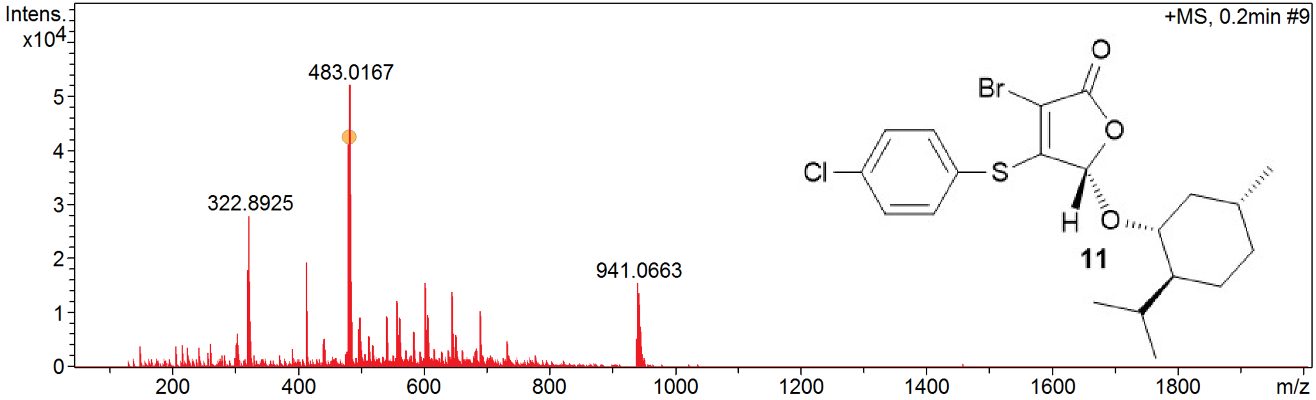

Meas. m/z	#	Ion Formula	m/z	err [ppm]	mSigma	# mSigma	Score	rdb	e• P Conf	N-Rule
481.0215	1	C20H24BrClNaO3S	481.0210	-1.0	22.4	1	100.00	7.5	even	ok

e)

Mass Spectrum SmartFormula Report

Analysis Info

Analysis Name D:\Data\User

Method lcms_esi_pos_low.m

Sample Name R6

Comment

Acquisition Date 2018/06/16 12:16:27

Operator BDAL@DE

Instrument micrOTOF II 8213750.10448

Acquisition Parameter

Source Type	ESI	Ion Polarity	Positive	Set Nebulizer	3.0 Bar
Focus	Not active			Set Dry Heater	200 • ‹C
Scan Begin	50 m/z	Set Capillary	4500 V	Set Dry Gas	10.0 l/min
Scan End	2000 m/z	Set End Plate Offset	-500 V	Set Divert Valve	Waste

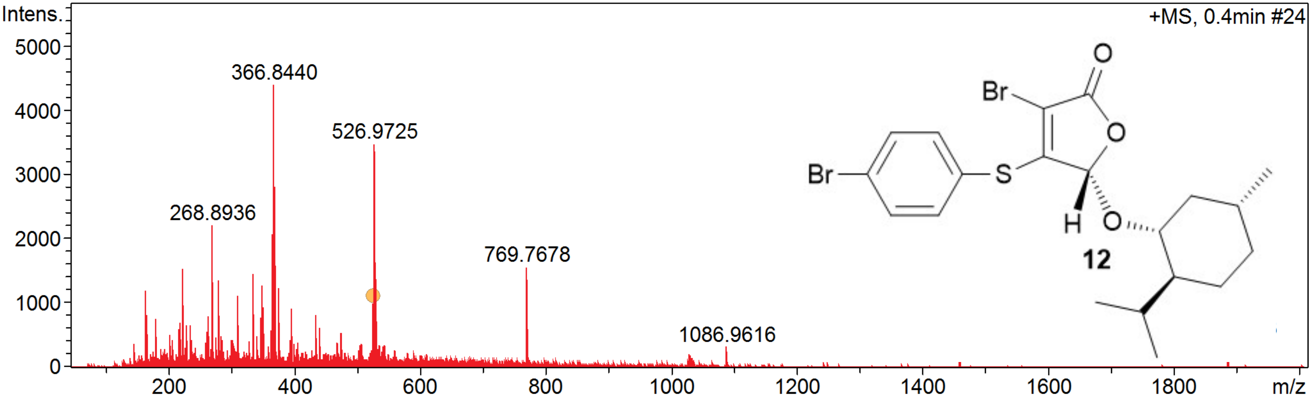

12

Meas. m/z	#	Ion Formula	m/z	err [ppm]	mSigma	# mSigma	Score	rdb	e• P Conf	N-Rule
524.9710	1	C20H24Br2NaO3S	524.9705	-0.9	96.0	1	100.00	7.5	even	ok

f)

Mass Spectrum SmartFormula Report

Analysis Info

Analysis Name	D:\Data\User	Acquisition Date	2018/03/22 12:35:47
Method	lcms_esi_pos_low.m	Operator	BDAL@DE
Sample Name	Rg-J3	Instrument	micrOTOF II 8213750.10448
Comment			

Acquisition Parameter

Source Type	ESI	Ion Polarity	Positive	Set Nebulizer	3.0 Bar
Focus	Not active			Set Dry Heater	200 • ‹C
Scan Begin	50 m/z	Set Capillary	4500 V	Set Dry Gas	10.0 l/min
Scan End	2000 m/z	Set End Plate Offset	-500 V	Set Divert Valve	Waste

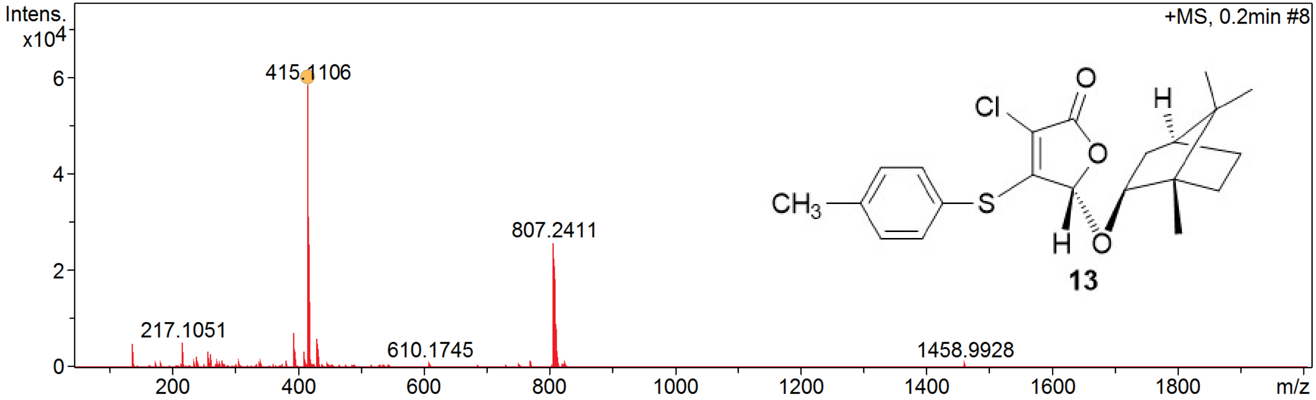

Meas. m/z	#	Ion Formula	m/z	err [ppm]	mSigma	# mSigma	Score	rdb	e• P Conf	N-Rule
415.1106	1	C21H25ClNaO3S	415.1105	-0.1	18.8	1	100.00	8.5	even	ok

h)

Mass Spectrum SmartFormula Report

Analysis Info

Analysis Name	D:\Data\User	Acquisition Date	2018/03/22 11:18:43
Method	lcms_esi_pos_low.m	Operator	BDAL@DE
Sample Name	Rg-B2	Instrument	micrOTOF II 8213750.10448
Comment			

Acquisition Parameter

Source Type	ESI	Ion Polarity	Positive	Set Nebulizer	3.0 Bar
Focus	Not active			Set Dry Heater	200 • ‹C
Scan Begin	50 m/z	Set Capillary	4500 V	Set Dry Gas	10.0 l/min
Scan End	2000 m/z	Set End Plate Offset	-500 V	Set Divert Valve	Waste

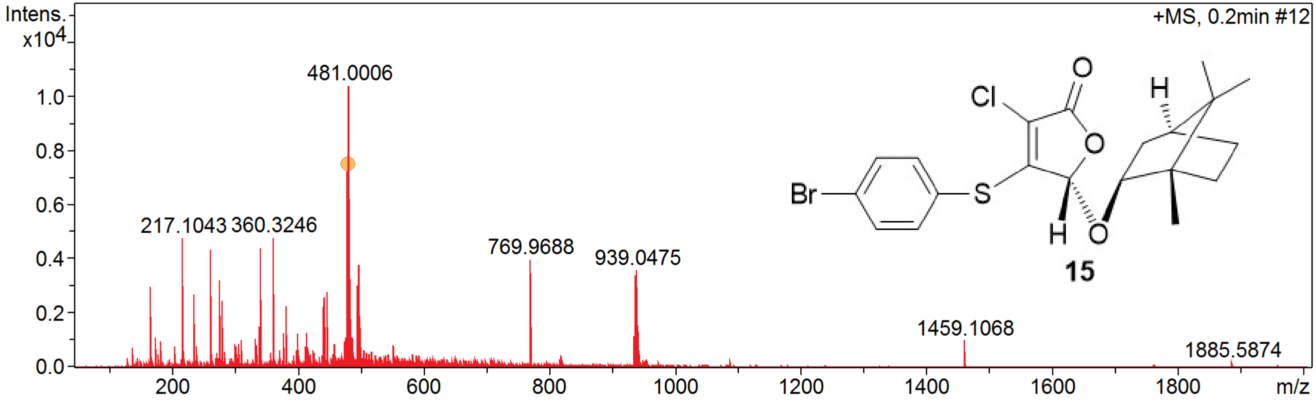

15

Meas. m/z	#	Ion Formula	m/z	err [ppm]	mSigma	# mSigma	Score	rdb	e•	P Conf	N-Rule
479.0062	1	C20H22BrClNaO3S	479.0054	-1.6	16.6	1	100.00	8.5	even		ok

i)

Mass Spectrum SmartFormula Report

Analysis Info

Analysis Name	D:\Data\User	Acquisition Date	2018/03/27 9:51:04
Method	lcms_esi_pos_low.m	Operator	BDAL@DE
Sample Name	Rg-S3	Instrument	micrOTOF II 8213750.10448
Comment			

Acquisition Parameter

Source Type	ESI	Ion Polarity	Positive	Set Nebulizer	3.0 Bar
Focus	Not active			Set Dry Heater	200 • ‹C
Scan Begin	50 m/z	Set Capillary	4500 V	Set Dry Gas	10.0 l/min
Scan End	2000 m/z	Set End Plate Offset	-500 V	Set Divert Valve	Waste

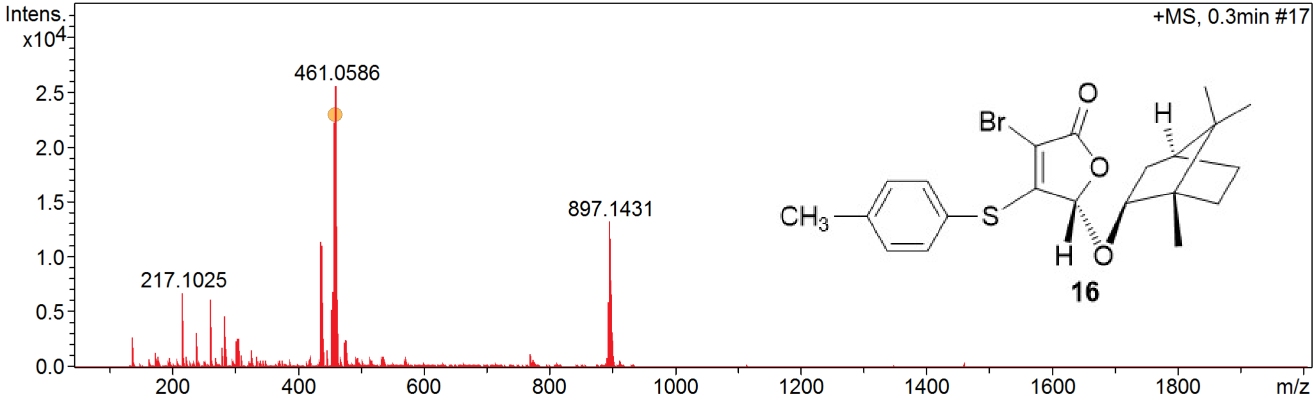

Meas. m/z	#	Ion Formula	m/z	err [ppm]	mSigma	# mSigma	Score	rdb	e• P Conf	N-Rule
459.0599	1	C21H25BrNaO3S	459.0600	0.2	34.8	1	100.00	8.5	even	ok

j)

Mass Spectrum SmartFormula Report

Analysis Info

Acquisition Date 2018/03/23 15:12:45

Analysis Name D:\Data\User

Method lcms_esi_pos_low.m

Sample Name Faz-L1

Comment

Operator BDAL@DE

Instrument micrOTOF II 8213750.10448

Acquisition Parameter

Source Type	ESI	Ion Polarity	Positive	Set Nebulizer	3.0 Bar
Focus	Not active			Set Dry Heater	200 • ‹C
Scan Begin	50 m/z	Set Capillary	4500 V	Set Dry Gas	10.0 l/min
Scan End	2000 m/z	Set End Plate Offset	-500 V	Set Divert Valve	Waste

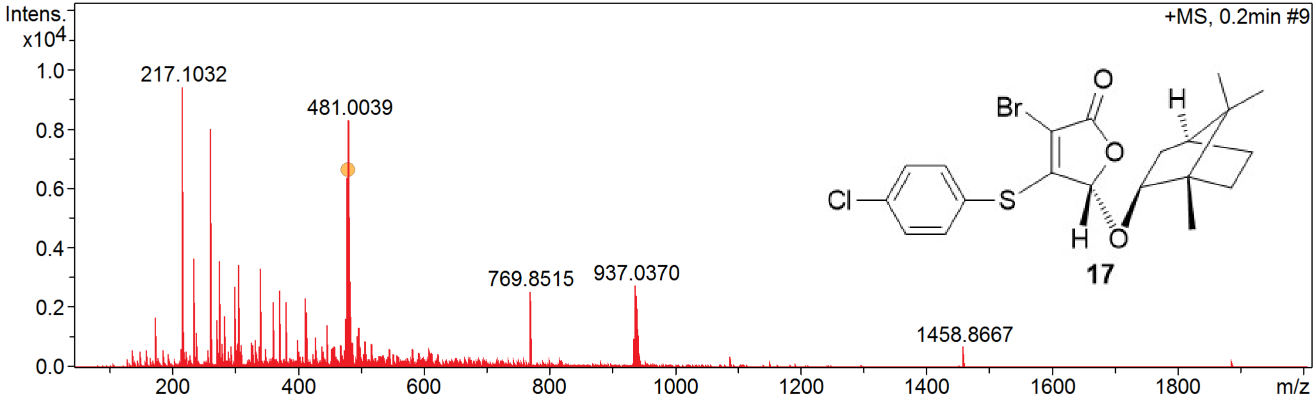

17

Meas. m/z	#	Ion Formula	m/z	err [ppm]	mSigma	# mSigma	Score	rdb	e•	P Conf	N-Rule
479.0056	1	C20H22BrClNaO3S	479.0054	-0.4	35.4	1	100.00	8.5	even		ok

k)

Mass Spectrum SmartFormula Report

Analysis Info

Analysis Name	D:\Data\User	Acquisition Date	2018/03/26 11:45:19
Method	lcms_esi_pos_low.m	Operator	BDAL@DE
Sample Name	Faz-T1	Instrument	micrOTOF II 8213750.10448
Comment			

Acquisition Parameter

Source Type	ESI	Ion Polarity	Positive	Set Nebulizer	3.0 Bar
Focus	Not active			Set Dry Heater	200 • ‹C
Scan Begin	50 m/z	Set Capillary	4500 V	Set Dry Gas	10.0 l/min
Scan End	2000 m/z	Set End Plate Offset	-500 V	Set Divert Valve	Waste

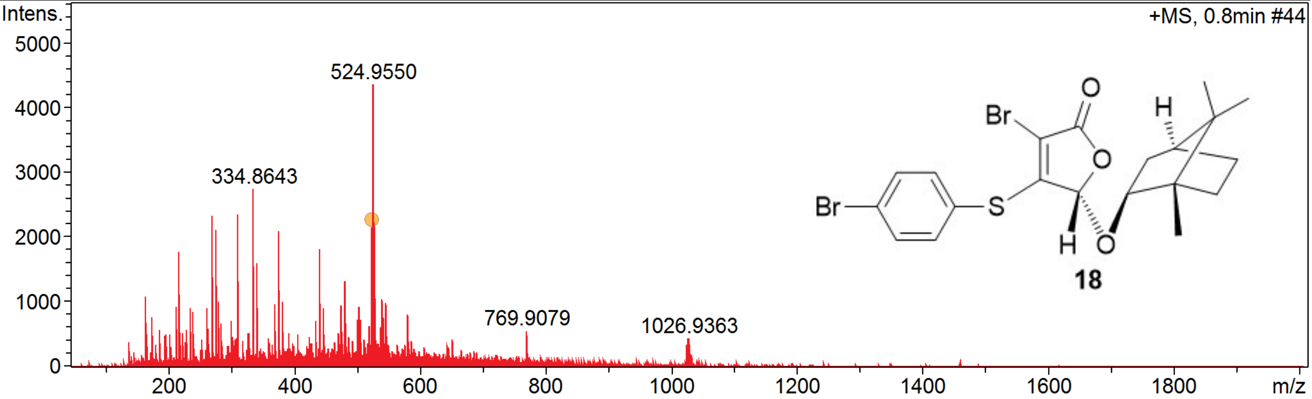

18

Meas. m/z	#	Ion Formula	m/z	err [ppm]	mSigma	# mSigma	Score	rdb	e•	P Conf	N-Rule
522.9556	1	C20H22Br2NaO3S	522.9549	-1.4	20.1	1	100.00	8.5	even		ok

Figure S23. Mass spectra of thioethers **8–18**.

Mass spectra of sulfones

a)

Mass Spectrum SmartFormula Report

Analysis Info

Analysis Name	D:\Data\User	Acquisition Date	2018/06/16 12:05:09
Method	lcms_esi_pos_low.m	Operator	BDAL@DE
Sample Name	R10	Instrument	micrOTOF II 8213750.10448
Comment			

Acquisition Parameter

Source Type	ESI	Ion Polarity	Positive	Set Nebulizer	3.0 Bar
Focus	Not active			Set Dry Heater	200 • ‹C
Scan Begin	50 m/z	Set Capillary	4500 V	Set Dry Gas	10.0 l/min
Scan End	2000 m/z	Set End Plate Offset	-500 V	Set Divert Valve	Waste

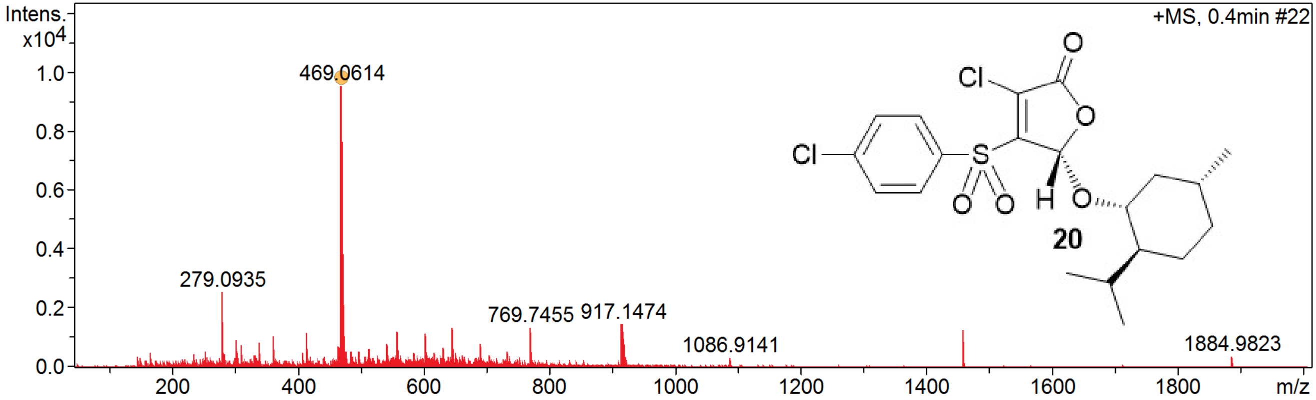

Meas. m/z	#	Ion Formula	m/z	err [ppm]	mSigma	# mSigma	Score	rdb	e•	P Conf	N-Rule
469.0614	1	C20H24Cl2NaO5S	469.0614	0.0	51.2	1	100.00	7.5	even		ok

b)

Mass Spectrum SmartFormula Report

Analysis Info

Analysis Name	D:\Data\User	Acquisition Date	2018/03/05 14:13:22
Method	lcms_esi_pos_low.m	Operator	BDAL@DE
Sample Name	ZR-E2	Instrument	micrOTOF II 8213750.10448
Comment			

Acquisition Parameter

Source Type	ESI	Ion Polarity	Positive	Set Nebulizer	3.0 Bar
Focus	Not active			Set Dry Heater	200 • ‹C
Scan Begin	50 m/z	Set Capillary	4500 V	Set Dry Gas	10.0 l/min
Scan End	2000 m/z	Set End Plate Offset	-500 V	Set Divert Valve	Waste

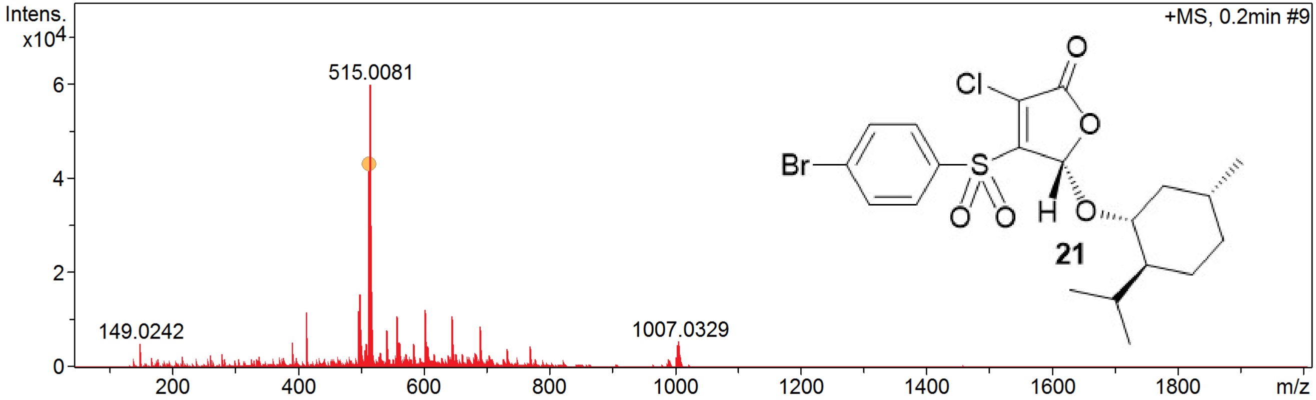

Meas. m/z	#	Ion Formula	m/z	err [ppm]	mSigma	# mSigma	Score	rdb	e•	P Conf	N-Rule
513.0110	1	C20H24BrClNaO5S	513.0109	-0.3	21.2	1	100.00	7.5	even		ok

c)

Mass Spectrum SmartFormula Report

Analysis Info

Analysis Name	D:\Data\User	Acquisition Date	2018/03/13 14:51:30
Method	lcms_esi_pos_low.m	Operator	BDAL@DE
Sample Name	ZR-I1	Instrument	micrOTOF II 8213750.10448
Comment			

Acquisition Parameter

Source Type	ESI	Ion Polarity	Positive	Set Nebulizer	3.0 Bar
Focus	Not active			Set Dry Heater	200 • ‹C
Scan Begin	50 m/z	Set Capillary	4500 V	Set Dry Gas	10.0 l/min
Scan End	2000 m/z	Set End Plate Offset	-500 V	Set Divert Valve	Waste

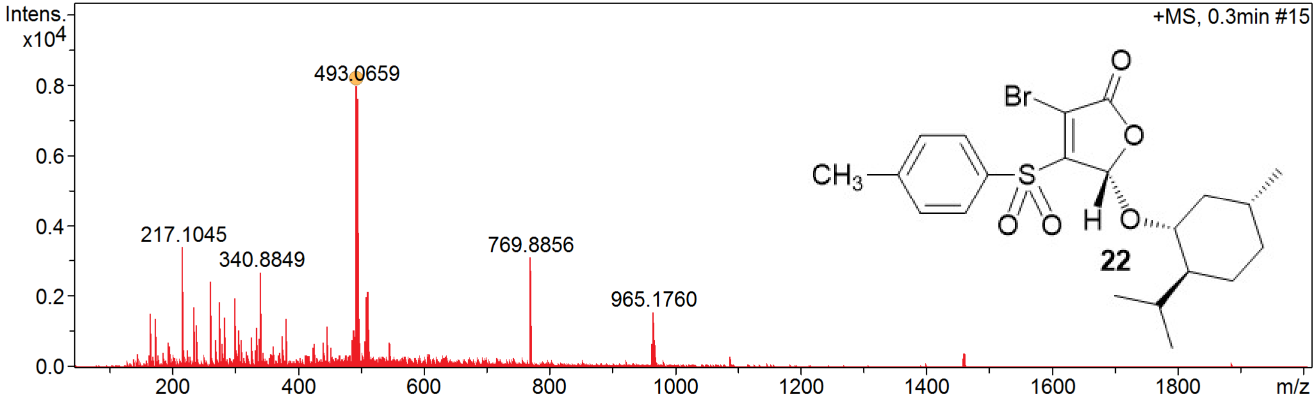

Meas. m/z	#	Ion Formula	m/z	err [ppm]	mSigma	# mSigma	Score	rdb	e•	P Conf	N-Rule
493.0659	1	C21H27BrNaO5S	493.0655	-0.8	35.2	1	100.00	7.5	even		ok

d)

Mass Spectrum SmartFormula Report

Analysis Info

Analysis Name	D:\Data\User	Acquisition Date	2018/03/05 14:13:22
Method	lcms_esi_pos_low.m	Operator	BDAL@DE
Sample Name	ZR-F1	Instrument	micrOTOF II 8213750.10448
Comment			

Acquisition Parameter

Source Type	ESI	Ion Polarity	Positive	Set Nebulizer	3.0 Bar
Focus	Not active			Set Dry Heater	200 • ‹C
Scan Begin	50 m/z	Set Capillary	4500 V	Set Dry Gas	10.0 l/min
Scan End	2000 m/z	Set End Plate Offset	-500 V	Set Divert Valve	Waste

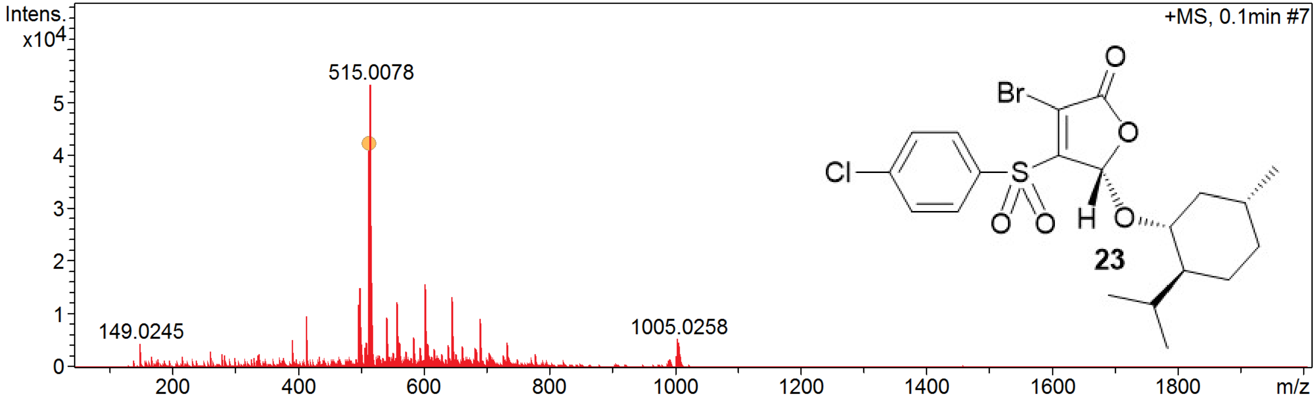

Meas. m/z	#	Ion Formula	m/z	err [ppm]	mSigma	# mSigma	Score	rdb	e•	P Conf	N-Rule
513.0106	1	C20H24BrClNaO5S	513.0109	0.5	17.3	1	100.00	7.5	even		ok

e)

Mass Spectrum SmartFormula Report

Analysis Info

Analysis Name	D:\Data\User	Acquisition Date	2018/03/13 14:47:13
Method	lcms_esi_pos_low.m	Operator	BDAL@DE
Sample Name	ZR-A1	Instrument	micrOTOF II 8213750.10448
Comment			

Acquisition Parameter

Source Type	ESI	Ion Polarity	Positive	Set Nebulizer	3.0 Bar
Focus	Not active			Set Dry Heater	200 • ‹C
Scan Begin	50 m/z	Set Capillary	4500 V	Set Dry Gas	10.0 l/min
Scan End	2000 m/z	Set End Plate Offset	-500 V	Set Divert Valve	Waste

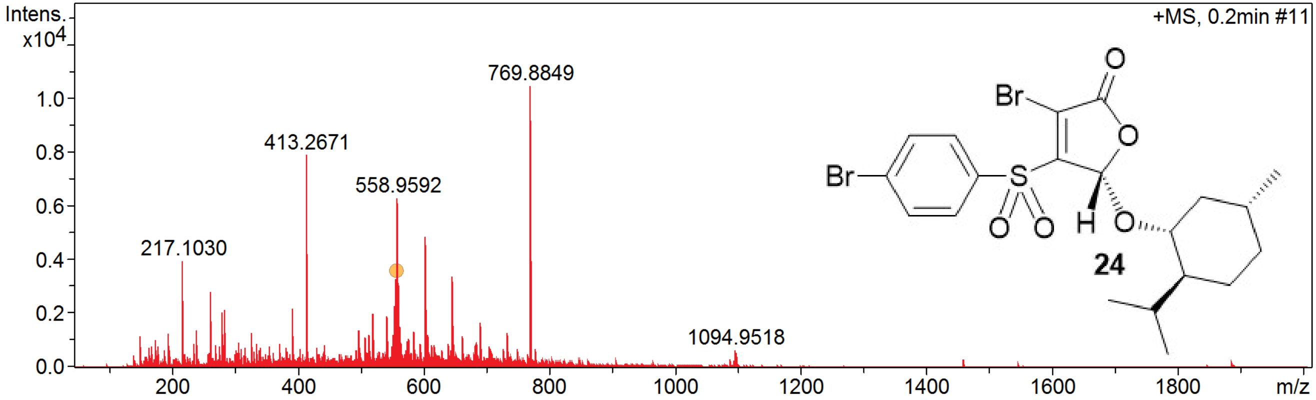

Meas. m/z	#	Ion Formula	m/z	err [ppm]	mSigma	# mSigma	Score	rdb	e• P Conf	N-Rule
556.9610	1	C20H24Br2NaO5S	556.9603	-1.1	31.7	1	100.00	7.5	even	ok

f)

Mass Spectrum SmartFormula Report

Analysis Info

Analysis Name	D:\Data\User Data\Regina\SR1_1-1_01_701.d	Acquisition Date	3/30/2017 4:18:23 PM
Method	lcms_esi_pos_low.m	Operator	BDAL@DE
Sample Name	SR1	Instrument	micrOTOF II 8213750.10448
Comment			

Acquisition Parameter

Source Type	ESI	Ion Polarity	Positive	Set Nebulizer	3.0 Bar
Focus	Not active			Set Dry Heater	200 °C
Scan Begin	50 m/z	Set Capillary	4500 V	Set Dry Gas	10.0 l/min
Scan End	2000 m/z	Set End Plate Offset	-500 V	Set Divert Valve	Waste

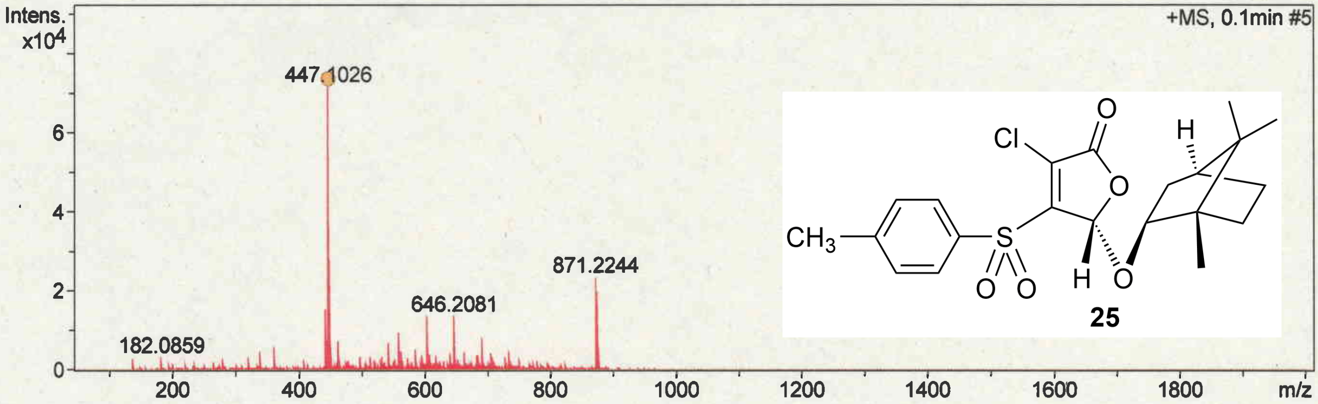

Meas. m/z	#	Ion Formula	m/z	err [ppm]	mSigma	# mSigma	Score	rdb	e⁻ Conf	N-Rule
447.1026	1	C21H25ClNaO5S	447.1003	-5.1	13.0	1	100.00	8.5	even	ok

h)

Mass Spectrum SmartFormula Report

Analysis Info

Analysis Name	D:\Data\User	Acquisition Date	2018/03/22 12:31:02
Method	lcms_esi_pos_low.m	Operator	BDAL@DE
Sample Name	Rg-Z1	Instrument	micrOTOF II 8213750.10448
Comment			

Acquisition Parameter

Source Type	ESI	Ion Polarity	Positive	Set Nebulizer	3.0 Bar
Focus	Not active			Set Dry Heater	200 • ‹C
Scan Begin	50 m/z	Set Capillary	4500 V	Set Dry Gas	10.0 l/min
Scan End	2000 m/z	Set End Plate Offset	-500 V	Set Divert Valve	Waste

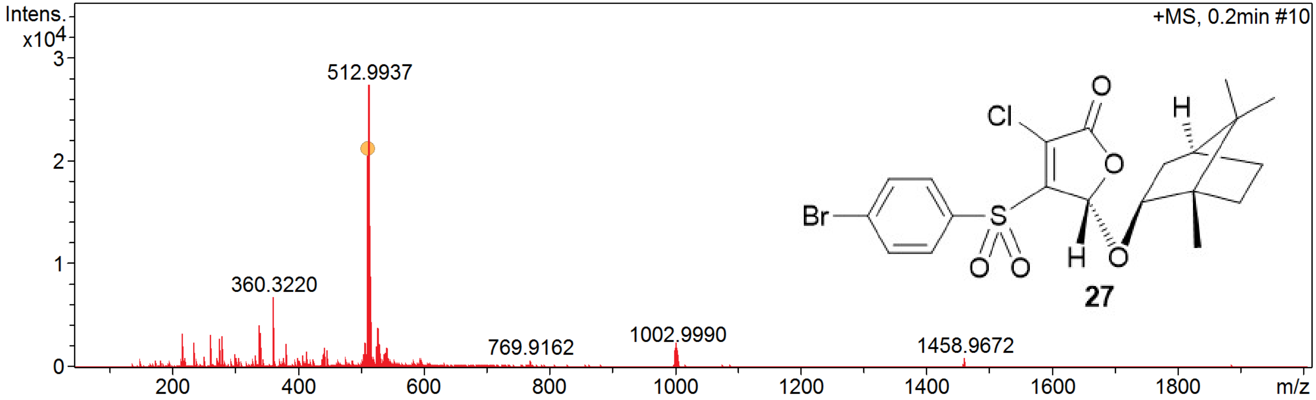

27

Meas. m/z	#	Ion Formula	m/z	err [ppm]	mSigma	# mSigma	Score	rdb	e•	P Conf	N-Rule
510.9956	1	C20H22BrClNaO5S	510.9952	-0.9	10.4	1	100.00	8.5	even		ok

i)

Mass Spectrum SmartFormula Report

Analysis Info

Analysis Name D:\Data\User

Method lcms_esi_pos_low.m

Sample Name Rg-G1

Comment

Acquisition Date 2018/03/23 15:08:58

Operator BDAL@DE

Instrument micrOTOF II 8213750.10448

Acquisition Parameter

Source Type	ESI	Ion Polarity	Positive	Set Nebulizer	3.0 Bar
Focus	Not active			Set Dry Heater	200 • ‹C
Scan Begin	50 m/z	Set Capillary	4500 V	Set Dry Gas	10.0 l/min
Scan End	2000 m/z	Set End Plate Offset	-500 V	Set Divert Valve	Waste

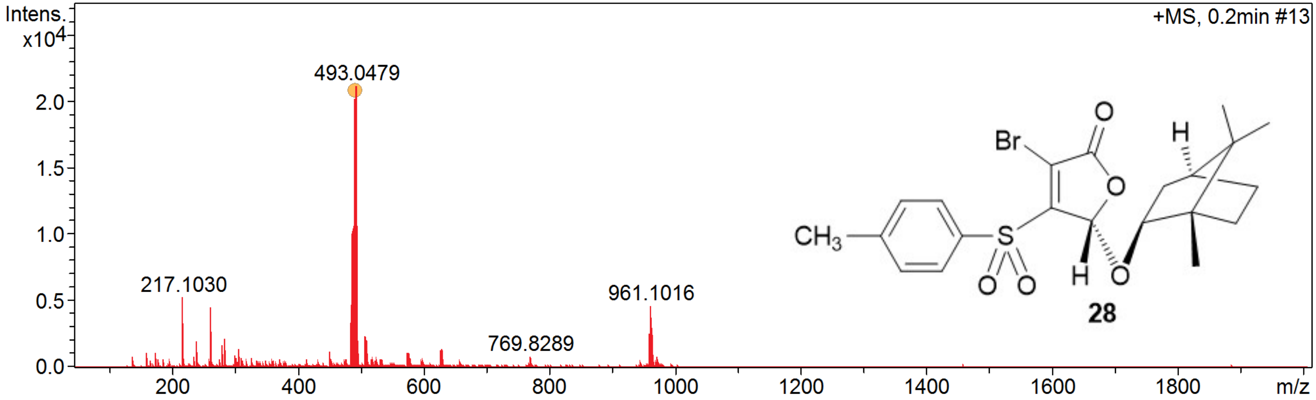

Meas. m/z	#	Ion Formula	m/z	err [ppm]	mSigma	# mSigma	Score	rdb	e•	P Conf	N-Rule
491.0498	1	C21H25BrNaO5S	491.0498	0.1	12.0	1	100.00	8.5	even		ok

j)

Mass Spectrum SmartFormula Report

Analysis Info

Analysis Name	D:\Data\User	Acquisition Date	2018/03/23 15:24:02
Method	lcms_esi_pos_low.m	Operator	BDAL@DE
Sample Name	Faz-X6	Instrument	micrOTOF II 8213750.10448
Comment			

Acquisition Parameter

Source Type	ESI	Ion Polarity	Positive	Set Nebulizer	3.0 Bar
Focus	Not active			Set Dry Heater	200 • ‹C
Scan Begin	50 m/z	Set Capillary	4500 V	Set Dry Gas	10.0 l/min
Scan End	2000 m/z	Set End Plate Offset	-500 V	Set Divert Valve	Waste

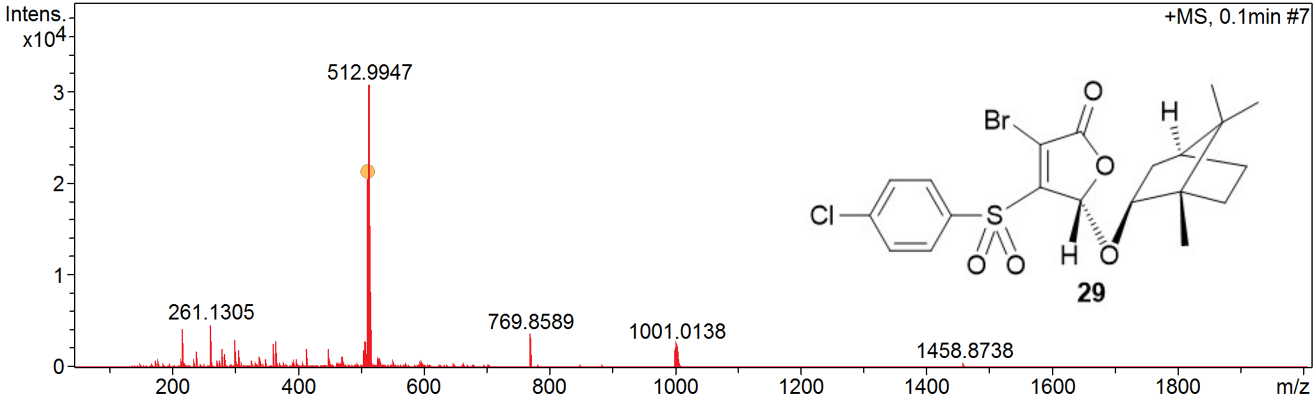

Meas. m/z	#	Ion Formula	m/z	err [ppm]	mSigma	# mSigma	Score	rdb	e•	P Conf	N-Rule
510.9950	1	C20H22BrClNaO5S	510.9952	0.4	30.8	1	100.00	8.5	even		ok

k)

Mass Spectrum SmartFormula Report

Analysis Info

Analysis Name	D:\Data\User Data\Regina\SR6_1-6_01_706.d	Acquisition Date	3/30/2017 4:37:27 PM
Method	lcms_esi_pos_low.m	Operator	BDAL@DE
Sample Name	SR6	Instrument	micrOTOF II 8213750.10448
Comment			

Acquisition Parameter

Source Type	ESI	Ion Polarity	Positive	Set Nebulizer	3.0 Bar
Focus	Not active			Set Dry Heater	200 °C
Scan Begin	50 m/z	Set Capillary	4500 V	Set Dry Gas	10.0 l/min
Scan End	2000 m/z	Set End Plate Offset	-500 V	Set Divert Valve	Waste

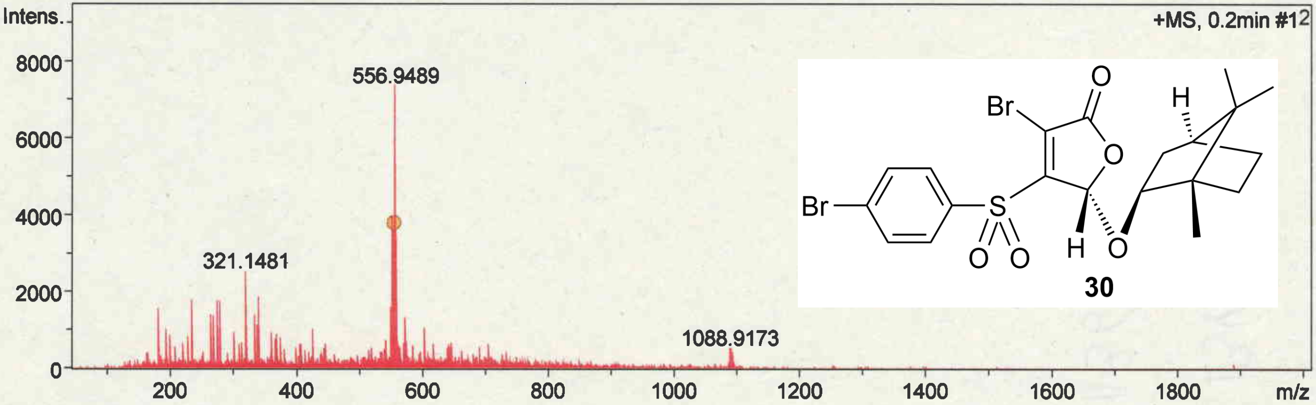

Meas. m/z	#	Ion Formula	m/z	err [ppm]	mSigma	# mSigma	Score	rdb	e⁻ Conf	N-Rule
554.9487	1	C20H22Br2NaO5S	554.9447	-7.2	23.6	1	100.00	8.5	even	ok

Figure S24. Mass spectra of sulfones **20–30**.

Single crystal X-ray analysis

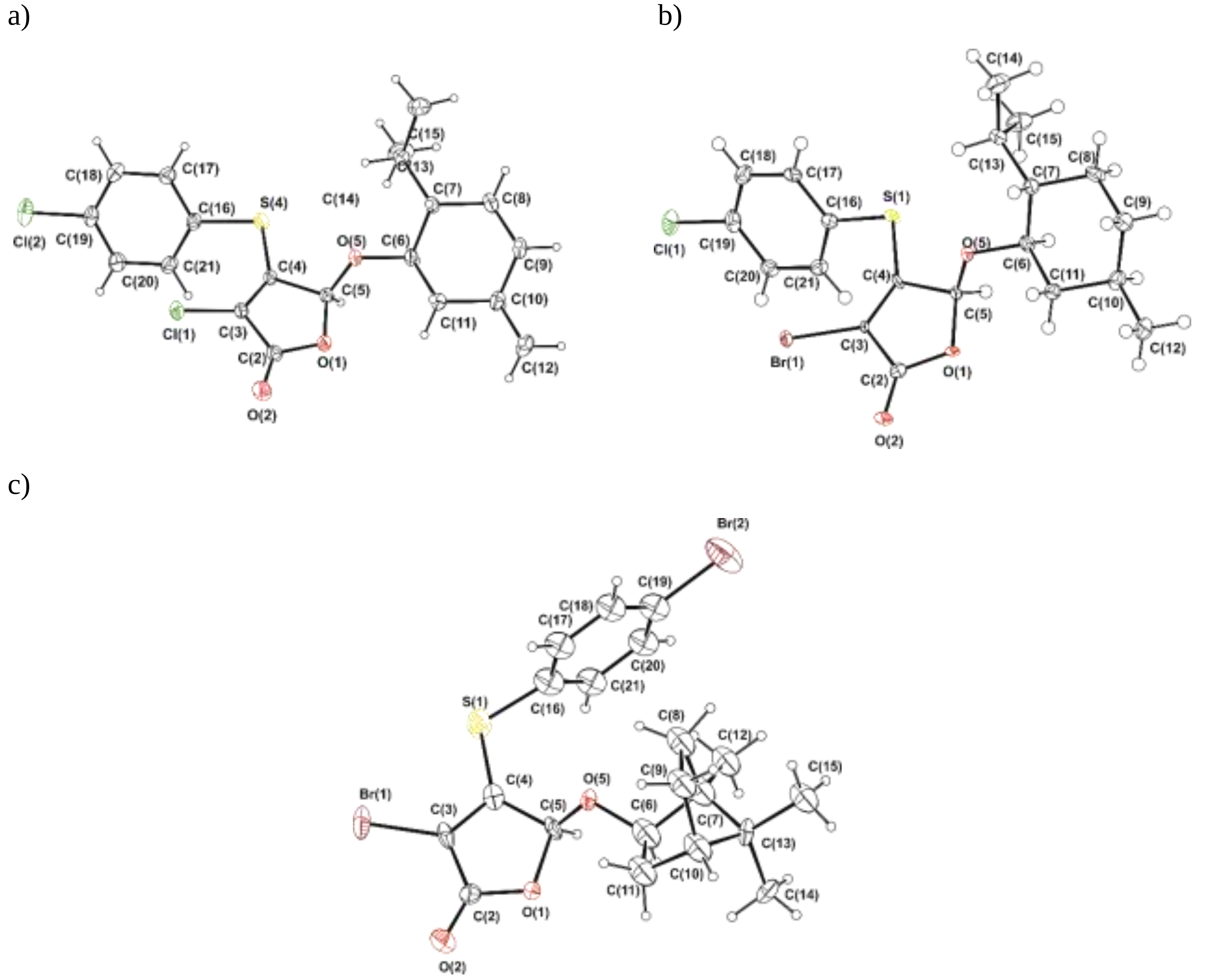

Figure S25. Molecular structure of thioethers **8** (a), **11** (b) and **18** (c) in the crystal.

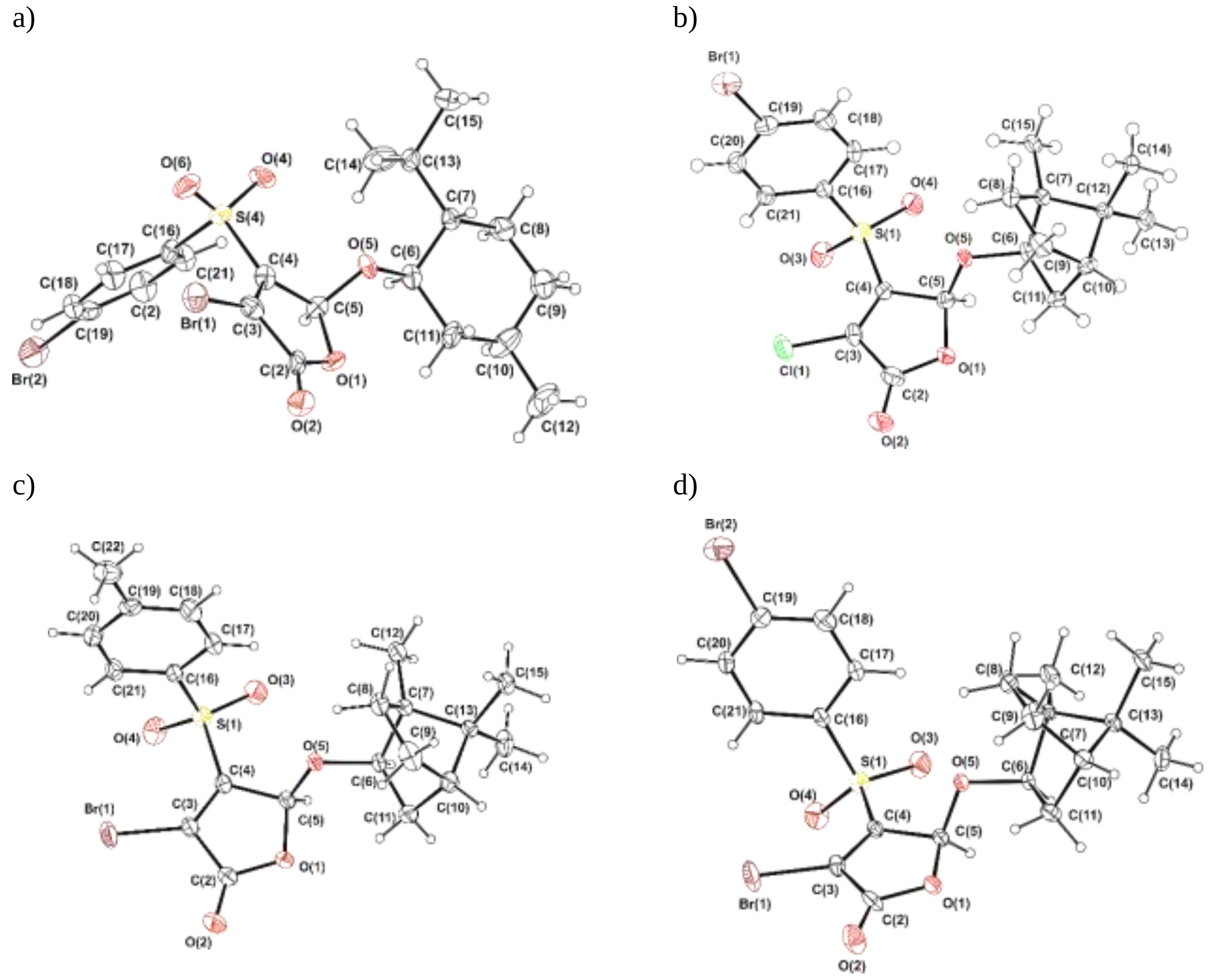

Figure S26. Molecular structure of sulfones **24** (a), **27** (b), **28** (c) and **30** (d) in the crystal.

Table S1. Crystal data, data collection and structure refinement details for crystals **5a**, **7**, **8**, **11**, **16**, **18**, **19**, **24**, **26–28** and **30**

Compound	**5a**	**7**	**8**	**11**	**16**	**18**
Empirical formula	$C_{14}H_{18}Cl_2O_3$	$C_{21}H_{27}ClO_3S$	$C_{20}H_{24}Cl_2O_3S$	$C_{20}H_{24}BrClO_3S$	$C_{21}H_{25}BrO_3S$	$C_{20}H_{22}Br_2O_3S$
Formula weight	305.18	394.93	415.35	459.81	437.37	502.24
Crystal system	orthorhombic	orthorhombic	monoclinic	monoclinic	trigonal	monoclinic
Space group	$P2_12_12_1$	$P2_12_12_1$	$P2_1$	$P2_1$	$P3_2$	$P2_1$
a/Å	7.384(4)	10.274(5)	6.637(3)	6.7968(4)	15.742(7)	7.2279(19)
b/Å	7.920(4)	10.768(5)	8.087(3)	8.0082(4)	15.742(7)	29.569(8)
c/Å	25.943(13)	19.382(9)	19.288(8)	18.6589(11)	7.496(14)	10.475(3)
α	90	90	90	90	90	90
β	90	90	92.015(5)	91.688(4)	90	109.608(7)
γ	90	90	90	90	120	90
Volume, Å^3	1517.2(14)	2144.3(17)	1034.6(7)	1015.17(10)	1609(4)	2108.9(10)
Z and Z’	4 and 1	4 and 1	2 and 1	2 and 1	3 and 1	4 and 2
Calculated density, g cm^{-3}	1.336	1.223	1.333	1.504	1.354	1.582
Absorption coefficient, mm^{-1}	0.429	0.292	0.431	2.276	2.030	3.959
F(000)	640	840	436	472	678	1008
Θ range for data collection	2.7° to 28.0°	2.1° to 28.0°	2.1° to 28.0°	3.7° to 28.0°	3.0° to 27.0°	3.6, 27.0
Index ranges	$-9 \le h \le 9$, $-10 \le k \le 4$, $-34 \le l \le 34$	$-13 \le h \le 13$, $-14 \le k \le 14$, $-25 \le l \le 24$	$-8 \le h \le 8$, $-10 \le k \le 10$, $-25 \le l \le 25$	$-8 \le h \le 8$, $-10 \le k \le 10$, $-24 \le l \le 24$	$-20 \le h \le 20$, $-19 \le k \le 20$, $-5 \le l \le 9$	$-4 \le h \le 9$, $-37 \le k \le 36$, $-13 \le l \le 13$
Reflections collected	13916	19674	9419	17063	13251	17907
Independent reflections [R(int)]	3643 [0.115]	5159 [0.079]	4844, 0.044	4796, 0.057	3245 [0.2383]	8928 [0.1070]
Observed Data [$I > 2\sigma(I)$]	2000	2606	3774	3975	1067	3638
Data / parameters	3643/212	5159/239	4844, 238	4796, 235	3245/178	8928/343
Goodness-of-fit on F^2	0.95	0.96	0.94	1.05	0.94	0.96
Final R indices [$I > 2\sigma(I)$]	R_1 = 0.0572, wR_2 = 0.0957	R_1 = 0.0512, wR_2 = 0.1019	R_1 = 0.0417 wR_2 = 0.0833	R_1 = 0.0483 wR_2 = 0.1046	R_1 = 0.0790 wR_2 = 0.1677	R_1 = 0.0840 wR_2 = 0.2011
R indices (all data)	R_1 = 0.1263, wR_2 = 0.1197	R_1 = 0.1207, wR_2 = 0.1265	R_1 = 0.0558 wR_2 = 0.0894	R_1 = 0.0658 wR_2 = 0.1115	R_1 = 0.2570 wR_2 = 0.2500	R_1 = 0.2144 wR_2 = 0.2646
Flack parameter	-0.06(7)	0.05(8)	-0.07(5)	-0.002(6)	0.05(4)	0.011(12)
Largest diff. peak and hole, e Å^{-3}	–0.21 and 0.21	-0.166 and 0.037	-0.227 and 0.186	-0.599 and 1.811	-0.339 and 0.327	-0.98 and 0.87

Table S1 continued. Crystal data, data collection and structure refinement details for crystals **5a**, **7**, **8**, **11**, **16**, **18**, **19**, **24**, **26–28** and **30**

Compound	**19**	**24**	**26**	**27**	**28**	**30**
Empirical formula	$C_{21}H_{27}ClO_5S$	$C_{20}H_{24}Br_2O_5S$	$C_{20}H_{22}Cl_2O_5S$	$C_{20}H_{22}BrClO_5S$	$C_{21}H_{25}BrO_5S$	$C_{20}H_{22}Br_2O_5S$
Formula weight	426.93	536.27	445.34	489.79	469.37	534.24
Crystal system	orthorhombic	orthorhombic	orthorhombic	orthorhombic	orthorhombic	orthorhombic
Space group	$P2_12_12_1$	$P2_12_12_1$	$P2_12_12_1$	$P2_12_12_1$	$P2_12_12_1$	$P2_12_12_1$
a/Å	6.371(3)	9.652(7)	10.154(3)	10.0530(13)	10.2462(19)	10.094(10)
b/Å	17.650(7)	9.994(7)	11.250(3)	11.2524(11)	11.346(2)	11.199(10)
c/Å	19.166(8)	23.147(15)	18.301(6)	18.191(2)	18.312(4)	18.418(17)
α	90	90	90	90	90	90
β	90	90	90	90	90	90
γ	90	90	90	90	90	90
Volume, Å^3	2155.2(16)	2233(3)	2090.6(11)	2057.8(4)	2128.8(7)	2082(3)
Z and Z'	4 and 1	4 and 1	4 and 1	4 and 1	4 and 1	4 and 1
Calculated density, g cm^{-3}	1.316	1.595	1.415	1.581	1.464	1.704
Absorption coefficient, mm^{-1}	0.303	3.751	0.439	2.258	2.058	4.023
F(000)	904	1080	928	1000	968	1072
Θ range for data collection	1.6° to 26.0°	3.9° to 27.0°	4.3, 27.0	2.7° to 27.0°	3.8, 28.0	3.8, 27.0
Index ranges	$-7 \le h \le 12$, $-21 \le k \le 21$, $-23 \le l \le 23$	$-7 \le h \le 12$, $-12 \le k \le 11$, $-29 \le l \le 29$	$-12 \le h \le 6$, $-14 \le k \le 14$, $-23 \le l \le 23$	$-12 \le h \le 12$, $-14 \le k \le 14$, $-23 \le l \le 22$	$-13 \le h \le 7$, $-14 \le k \le 14$, $-24 \le l \le 24$	$-9 \le h \le 12$, $-13 \le k \le 14$, $-22 \le l \le 23$
Reflections collected	20910	16527	17858	25768	19651	17894
Independent reflections [R(int)]	4089, 0.167	4851, 0.248	4542 [0.056]	4467, 0.105	5115 [0.082]	4543 [0.146]
Observed Data [$I > 2\sigma(I)$]	2062	1748	3494	3309	2844	2237
Data / parameters	4089, 257	4851, 256	4542/253	4467, 256	5115/257	4543/256
Goodness-of-fit on F^2	1.05	0.89	1.03	0.99	1.01	1.02
Final R indices [$I > 2\sigma(I)$]	$R_1 = 0.0976$ $wR_2 = 0.0853$	$R_1 = 0.0723$ $wR_2 = 0.1155$	$R_1 = 0.0523$ $wR_2 = 0.1054$	$R_1 = 0.0455$ $wR_2 = 0.0776$	$R_1 = 0.0511$ $wR_2 = 0.1000$	$R_1 = 0.0582$ $wR_2 = 0.1128$
R indices (all data)	$R_1 = 0.2105$ $wR_2 = 0.1048$	$R_1 = 0.2427$ $wR_2 = 0.1666$	$R_1 = 0.0735$ $wR_2 = 0.1152$	$R_1 = 0.0759$ $wR_2 = 0.0879$	$R_1 = 0.1225$ $wR_2 = 0.1233$	$R_1 = 0.1563$ $wR_2 = 0.1462$
Flack parameter	-0.04(6)	0.02(3)	-0.09(4)	-0.005(9)	-0.007(6)	-0.019(13)
Largest diff. peak and hole, e Å^{-3}	-0.273 and 0.235	-0.523 and 0.549	-0.25 and 0.30	-0.463 and 0.423	-0.31 and 0.58	-0.65 and 0.43
